# Aromatic interactions with heterocycles in water†

**DOI:** 10.1039/d3sc03824f

**Published:** 2023-09-29

**Authors:** Gloria Tobajas-Curiel, Qingqing Sun, Jeremy K. M. Sanders, Pablo Ballester, Christopher A. Hunter

**Affiliations:** a Yusuf Hamied Department of Chemistry, University of Cambridge Cambridge CB2 1EW UK herchelsmith.orgchem@ch.cam.ac.uk; b Institute of Chemical Research of Catalonia (ICIQ), Barcelona Institute of Science and Technology (BIST) Av. Països Catalans, 16 43007 Tarragona Spain pballester@iciq.es; c Yangzhou University, School of Chemistry and Chemical Engineering Yangzhou 225002 Jiangsu China sunqingqing@snnu.edu.cn; d ICREA Passeig Lluís Companys 23 08010 Barcelona Spain

## Abstract

Conformationally well-defined supramolecular complexes that can be studied in different solvents provide a platform for separating and quantifying free energy contributions due to functional group interactions and desolvation. Here 1:1 complexes formed between four different calix[4]pyrrole receptors and eleven different pyridine *N*-oxide guests have been used to dissect the factors that govern aromatic interactions with heterocycles in water and in chloroform solution. ^1^H NMR spectroscopy shows that the three-dimensional structures of the complexes are fixed by four H-bonding interactions between the pyrrole donors at the bottom of the receptor and the *N*-oxide acceptor on the guest, locking the geometrical arrangement of interacting functional groups in the binding pocket at the other end of the receptor. An aromatic heterocycle on the guest makes two stacking interactions and two edge-to-face interactions with the side walls of the receptor. Chemical double mutant cycles were used to measure the free energy contribution of these four aromatic interactions to the overall stability of the complex. In chloroform, the aromatic interactions measured with pyridine, pyrimidine, furan, thiophene and thiazole are similar to the interactions with a phenyl group, but the effect of introducing a heteroatom depends on where it sits with respect to the aromatic side-walls of the cavity. A nitrogen lone pair directed into a π-face of the side-walls of the binding site leads to repulsive interactions of up to 8 kJ mol^−1^. In water, the heterocycle aromatic interactions are all significantly more favourable (by up to 12 kJ mol^−1^). For the non-polar heterocycles, furan and thiophene, the increase in interaction energy correlates directly with hydrophobicity, as measured by the free energy of transfer of the heterocycle from *n*-hexadecane into water (Δ*G*°(water–hex)). For the heterocycles with polar nitrogen H-bond acceptors, water can access cracks in the walls of the receptor binding site to solvate the edges of the heterocycles without significantly affecting the geometry of the aromatic interactions, and these nitrogen–water H-bonds stabilise the complexes by about 15 kJ mol^−1^. The results highlight the complexity of the solvation processes that govern molecular recognition in water.

## Abstract 2

Measurements using chemical double mutant cycles show that non-covalent interactions between heterocyclic and aromatic rings are more favourable in water than chloroform, provided polar sites are solvated by H-bonded water molecules in the complex.

## Graphical abstract

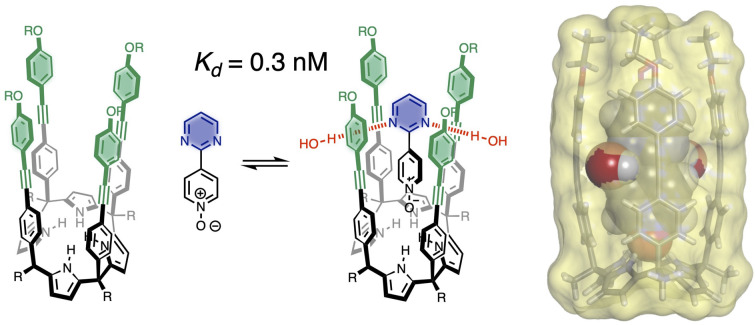

## Introduction

Aromatic heterocycles are key components in medicinal chemists' armoury and prevalent in approved drugs because the amphipathic nature allows for both polar H-bonding interactions and non-polar aromatic interactions with protein binding sites.^1–3^ Disentangling the relative importance of polar and non-polar interactions and desolvation effects from all of the other properties that govern the stability of protein–ligand complexes is difficult in such complicated systems.^4^ The relative simplicity of supramolecular complexes makes them ideally suited to systematic studies of the properties of non-covalent interactions, the relationship between chemical structure and binding affinity and the role of desolvation.^5^ Here, we use the well-defined complexes formed by super-aryl-extended calix[4]pyrroles and pyridine *N*-oxides^6–8^ to quantify aromatic interactions with a variety of different heterocycles both in water and in chloroform. The results show that remarkable binding affinities can be obtained in water (nanomolar dissociation constants) if the non-polar surfaces of the heterocycles are desolvated, but H-bonds with the solvent are maintained at the polar sites.

Compared with efforts to quantify aromatic interactions between hydrocarbon π-systems,^9–11^ there have been relatively few experimental studies on aromatic interactions with heterocycles. Dougherty found that the electron poor heterocycles quinoline and isoquinoline have a higher affinity for an aromatic cyclophane in water than the electron rich heterocycle indole, and attributed the difference to differences in the electrostatic interactions with the π-electron density of the host.^12^ Gellman investigated intramolecular interactions between covalently linked aromatic hydrocarbons and heterocycles in water and found that stacking interactions involving heterocycles were consistently more favourable than the interaction between two hydrocarbons. Again, the result was attributed to electrostatic interactions associated with the distribution of partial positive and negative charges across the π-face of the heterocycle.^13,14^ More recent experiments using molecular torsion balances show that aromatic stacking interactions involving heterocycles are also more favourable than interactions between aromatic hydrocarbons in organic solvents.^15,16^

Chemical double mutant cycles (DMC) have been used to measure the edge-to-face interactions between pyridine, furan, thiophene and pyrrole and the face of an aromatic hydrocarbon in chloroform.^17,18^ The results were rationalised based on electrostatic interactions that depend on the polarity of the protons on the edge of the heterocycle and the π-electron density on the face of the aromatic hydrocarbon. Attractive interactions of pyridine, furan and thiophene with the face of an aromatic hydrocarbon were also observed using molecular torsion balances in chloroform.^19^ The conformational properties of cyclophanes have been used to investigate repulsive interactions of the lone pairs of pyridine, furan and thiophene with the π-faces of aromatic hydrocarbons in organic solvents.^20,21^

**Fig. 1:**
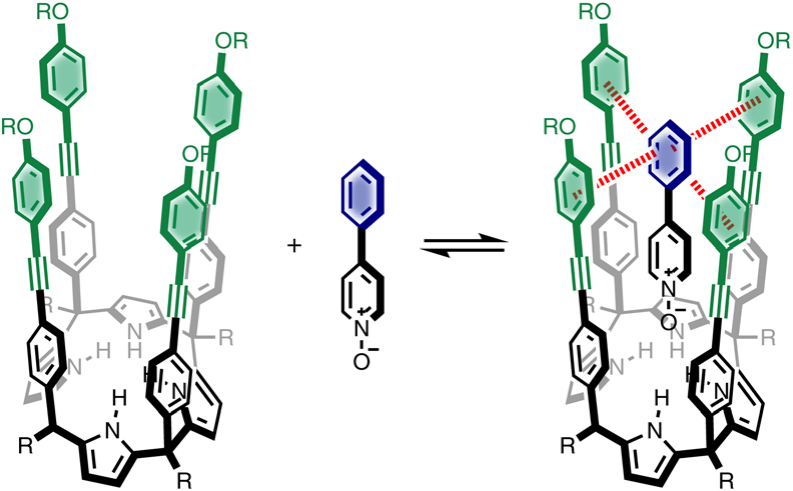
Complex formed by a super-aryl-extended calix[4]pyrrole and 4-phenylpyridine *N*-oxide, highlighting aromatic interactions between the blue ring of the guest and the four green rings of the host.

We recently described a supramolecular system for measuring substituent effects on aromatic interactions in water,^22^ and here we show that the approach can be extended to quantify aromatic interactions with a wide range of different heterocycles. Fig. 1 shows the structure of the complex formed by a super-aryl-extended calix[4]pyrrole and a pyridine *N*-oxide guest. The location of the guest in the binding pocket is fixed by H-bonding interactions between the pyrrole NH protons and the *N*-oxide group which also locks the host in the cone conformation. These interactions place the blue aromatic ring of the guest in a pocket created by the four green aromatic rings of the host, resulting in the two edge-to-face and two aromatic stacking interactions indicated in Fig. 1. The free energy contributions of these aromatic interactions to the stability of the host-guest complex were measured using chemical double mutant cycles (DMC) for a variety of different substituted phenyl groups on the guest. In addition, the solubility of the receptor can be modulated without any impact on the binding pocket by changing the nature of the peripheral R groups in Fig. 1. Thus, it was possible to directly compare the results of DMC experiments carried out in water with precisely the same measurements made in chloroform.

**Fig. 2:**
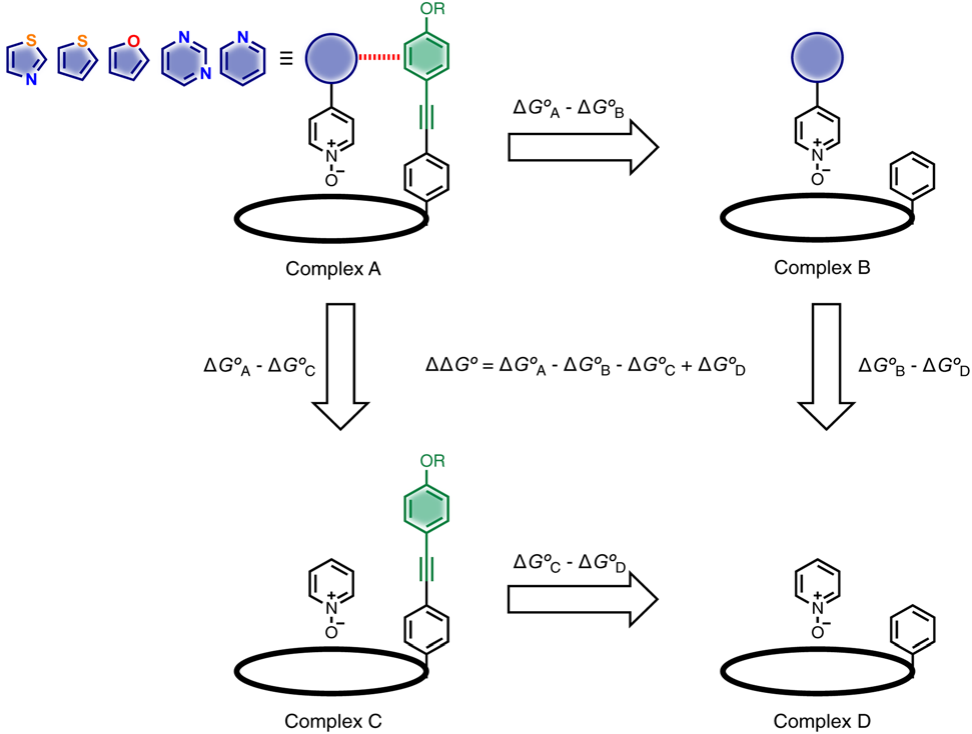
Chemical double mutant cycle for evaluating the free energy contribution (ΔΔ*G*°) due to aromatic interactions with the blue ring of the guest to the overall stability of complex A. Only one of the four calix[4]pyrrole side-arms is shown for clarity, but the DMC measures the sum of interactions with four green aromatic rings on the host.

Here, the complex shown in Fig. 1 is adapted in order to make quantitative measurements of non-covalent interactions between heterocycles and aromatic rings in water and in chloroform solution. The approach is illustrated in Fig. 2. If the blue aromatic ring of the guest in Fig. 1 is replaced by a heterocycle, then the interaction of the heterocycle with the four green aromatic rings of the host can be measured using the DMC shown in Fig. 2. Five different heterocycles were investigated, and the site of attachment to the pyridine *N*-oxide was varied in order to modulate the orientation of the heterocycle with respect to the aromatic side-walls of the binding pocket (see below). Complex A in Fig. 2 only shows one host-guest aromatic interaction for simplicity, but the DMC measures the sum of all four interactions that the blue heterocycle on the guest makes with the four green aromatic rings of the host. In principle, it might be possible to estimate the contribution of the aromatic interactions to the stability of complex A by simply comparing with complex C, which lacks the blue aromatic ring. However, as we have shown previously, the blue aromatic ring changes the H-bond acceptor properties of the pyridine *N*-oxide,^22^ so the difference between the free energies of formation of complexes A and C contains a contribution from the change in H-bond strength as well as the aromatic interactions. Comparison of complexes B and D of the DMC provides a measurement of the free energy contribution due the change in H-bond strength in the absence of the aromatic interactions of interest. Thus eqn (1) is used to obtain a measurement of the free energy contribution of the four aromatic interactions between the blue and green rings in complex A without any complications due to changes in H-bond strength.1
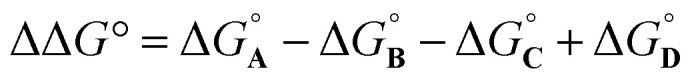


## Results

**Fig. 3:**
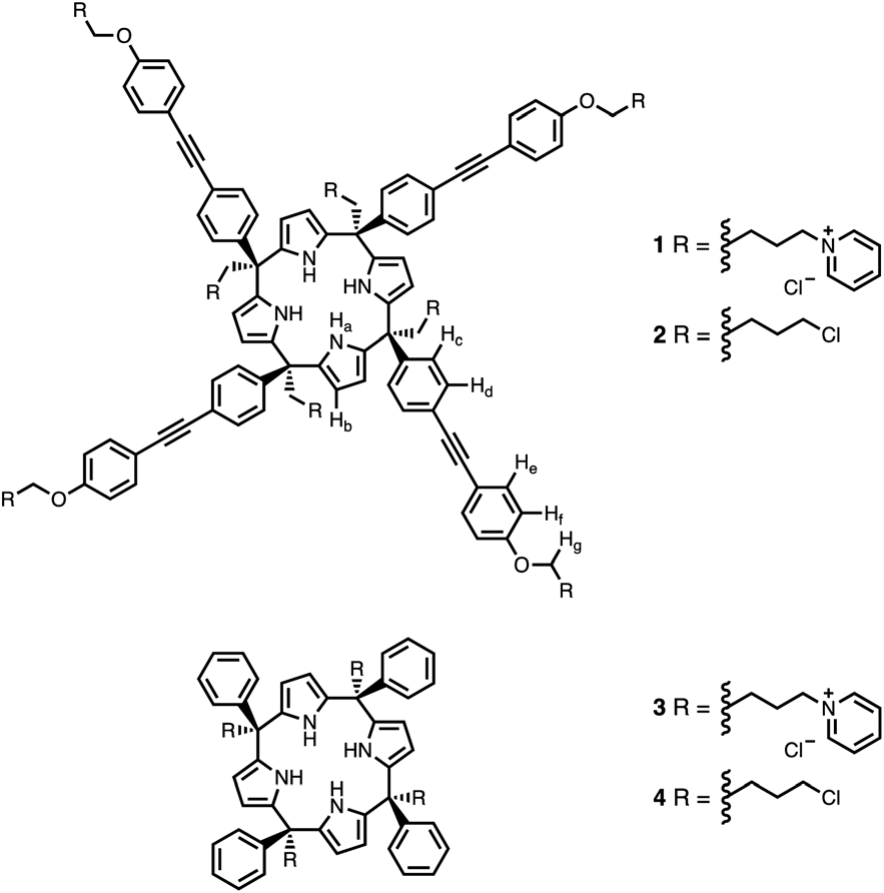
Chemical structures of calix[4]pyrroles 1–4 with the ^1^H NMR proton labelling scheme for 1 and 2.

**Scheme 1:**
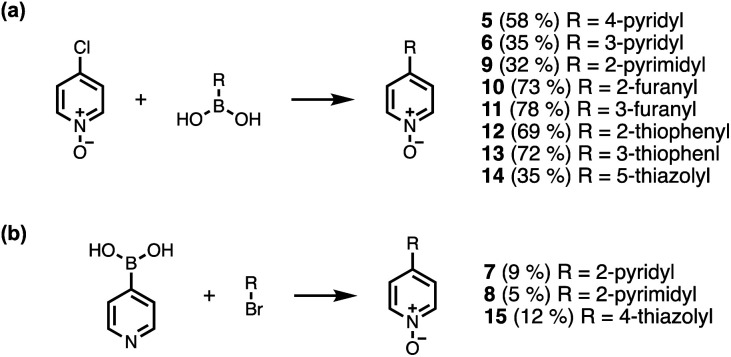
Synthesis of pyridine *N*-oxides. Reagents and conditions: (a) Pd(PPh_3_)_4_, Na_2_CO_3_, dioxane/H_2_O, 80 °C, 16 h. (b) (1) Pd(PPh_3_)_4_, Na_2_CO_3_, dioxane/H_2_O, 80 °C, 16 h; (2) mCPBA, DCM, rt, 16 h.

**Fig. 4:**
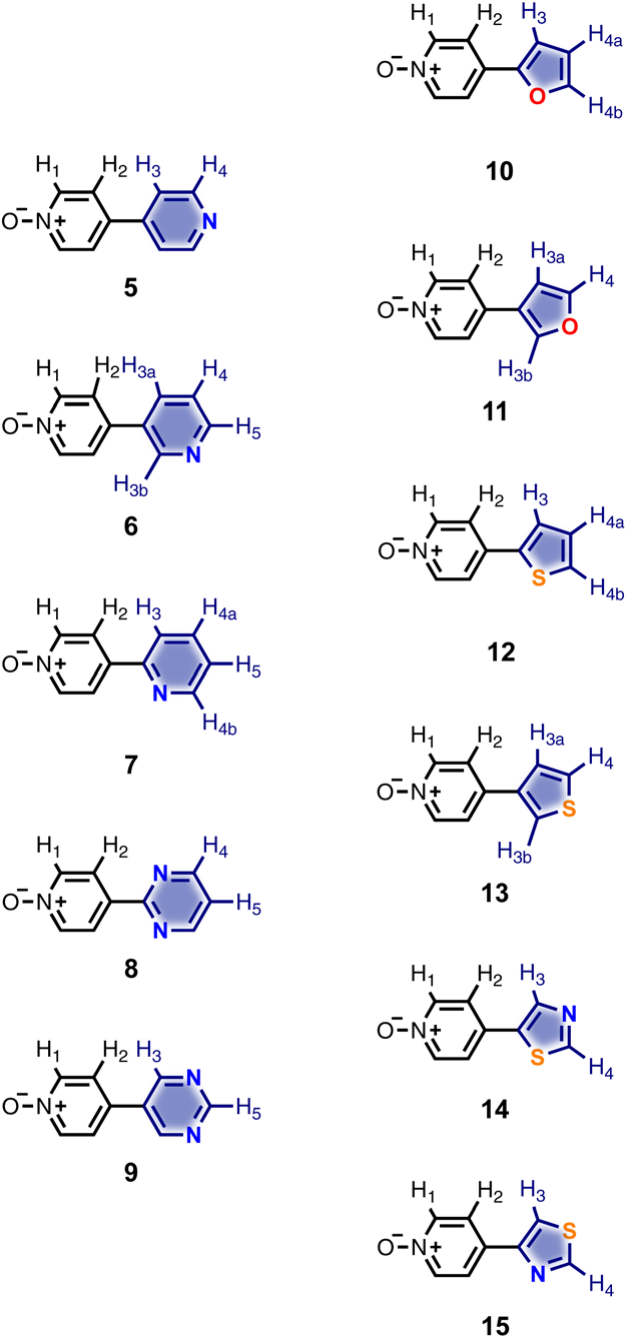
Chemical structures of pyridine *N*-oxide guests 5–15 showing the ^1^H NMR proton labelling scheme. An unconventional numbering is used in order to compare protons that sit in similar positions in the receptor cavity.

Calix[4]pyrrole receptors 1–4 shown in Fig. 3 were synthesised according to previously reported procedures.^7,23,24^ A series of pyridine *N*-oxide guests equipped with heterocyclic groups were prepared by Suzuki coupling as illustrated in Scheme 1. Fig. 4 shows the structures of the eleven guests and the proton labelling scheme used below to discuss the ^1^H NMR spectra.

**Fig. 5:**
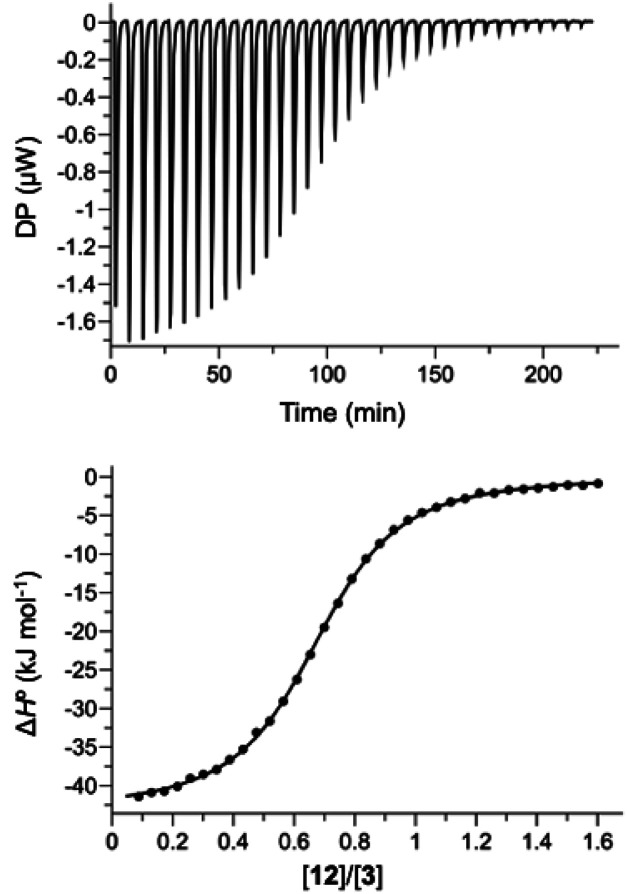
ITC data for titration of 12 (0.30 mM) into 3 (0.04 mM) in water at 298 K. The raw data for each injection is shown (differential power, DP), along with the least-squares-fit of the enthalpy change per mole of guest (Δ*H*°) to a 1:1 binding isotherm.

Hosts 1 and 3 were used to investigate binding of the heterocyclic pyridine *N*-oxide guests in water, and hosts 2 and 4 were used to carry out the same experiments in chloroform. The thermodynamic properties of complexes B, C and D of the DMC shown in Fig. 2 were determined using isothermal titration calorimetry (ITC). Fig. 5 shows typical titration data. The smooth sigmoidal transition from free to bound states in Fig. 5 means that the data can be fitted to a 1:1 binding isotherm to obtain both the association constant (*K*) and enthalpy change for formation of the 1:1 complex (Δ*H*°). The corresponding free energy change (Δ*G*° = −*RT* ln *K*) and entropy change (*T*Δ*S*° = Δ*H*° + *RT* ln *K*) associated with formation of the 1:1 complex were calculated directly from these measurements, and the results are reported in Tables S1 and S2.†

The stabilities of complex A of the DMC are too high to be measured by ITC, so ^1^H NMR competition experiments were used to measure the association constants for these complexes. We have shown previously that for complexes where the association constant is in a range that allows measurement using both methods, the results are in good agreement, which means that ITC and NMR measurements of association constant can be used interchangeably in the DMC.^22,25^ The ^1^H NMR competition experiment requires a starting reference complex for which the association constant has been reliably measured by a different technique. The complexes formed by phenylpyridine *N*-oxide with receptor 1 in water and with receptor 2 in chloroform were used as reference points for the experiments described here.^7,22^

**Fig. 6:**
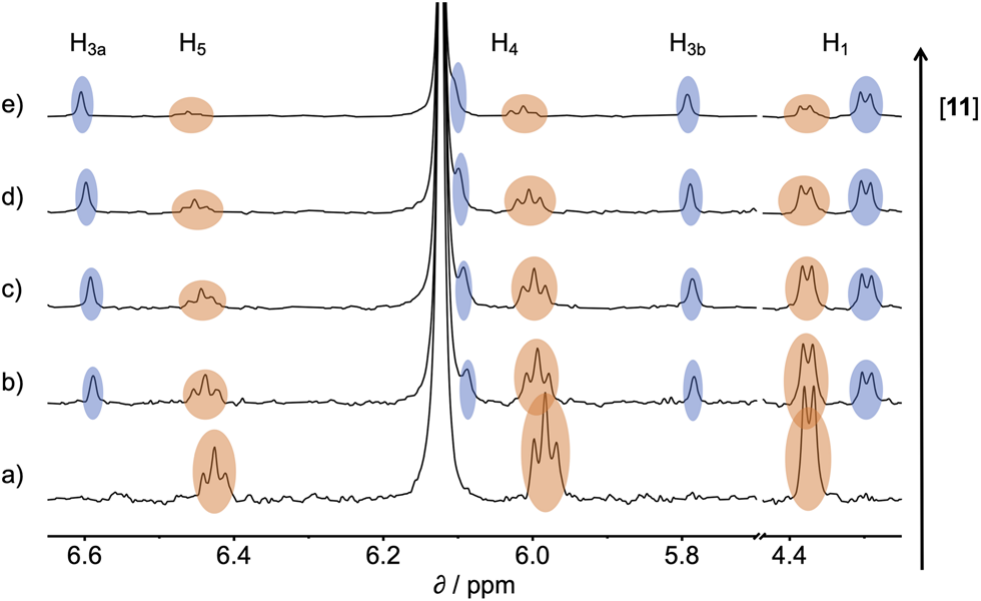
Partial 500 MHz ^1^H NMR for titration of 11 into a mixture of 1 and PPNO in D_2_O at 298 K. (a) 1: 0.38 mM; PPNO: 0.44 mM; 11: 0 mM; (b) 1: 0.31 mM; PPNO: 0.36 mM; 11: 0.60 mM; (c) 1: 0.27 mM; PPNO: 0.32 mM; 11: 0.89 mM; (d) 1: 0.22 mM; PPNO: 0.26 mM; 11: 1.31 mM; (e) 1: 0.16 mM; PPNO: 0.19 mM; 11: 1.81 mM. Signals due to the 1·11 complex are highlighted in blue, and signals due to the 1·PPNO complex are highlighted in orange. See Fig. 4 for the proton labelling scheme.

^1^H NMR spectra were recorded for mixtures of each of guests 5–15 and either 1 in deuterium oxide or 2 in deuterochloroform. In all cases, the signals due to the free and bound species were in slow exchange on the ^1^H NMR timescale. Fig. 6 shows typical data from a ^1^H NMR titration. Addition of phenylpyridine *N*-oxide (PPNO) to 1 lead to quantitative formation of the 1:1 complex. When small amounts of the second guest 11 were added to this mixture, three sets of signals were observed, corresponding to the two different host·guest complexes, 1·PPNO and 1·11, and free PPNO, all in slow exchange. As increasing concentrations of 11 were added, the signals due to the 1·PPNO complex (highlighted in orange in Fig. 6) decreased in intensity, and the intensities of the signals due to free PPNO and the 1·11 complex (highlighted in blue) increased. These observations show that 11 displaces PPNO from the receptor and that both the 1·PPNO and 1·11 complexes are present in equilibrium. The integrals of the ^1^H NMR signals (*I*) can be used to directly measure the concentrations of all species present and hence determine the equilibrium constant for guest exchange (eqn (2)).2
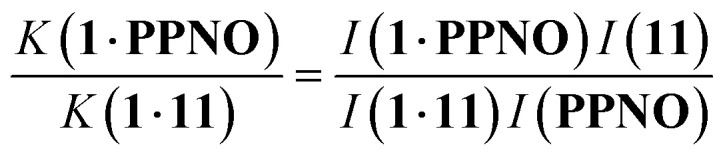


In this case, one of the two association constants *K*(1·PPNO) is known, so the association constant for the 1·11 complex can be determined by averaging the values obtained from eqn (2) for each spectrum in the titration experiment. The association constants (*K*) measured using pairwise NMR competition experiments are reported in Tables S1 and S2,† along with the corresponding free energy changes (Δ*G*°).

**Fig. 7:**
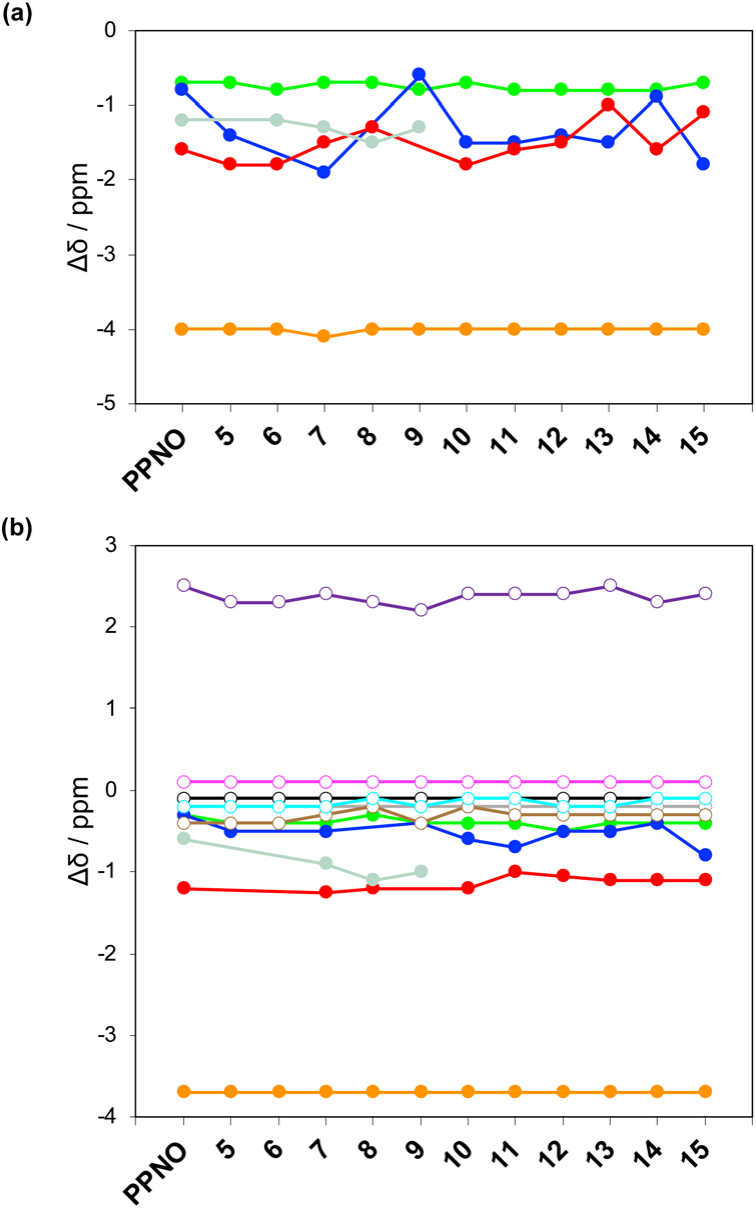
Limiting complexation-induced changes in ^1^H NMR chemical shift (Δ*δ* in ppm) for complex A of the DMC. (a) Guest signals in deuterium oxide. (b) Guest (filled circles) and host (open circles) signals in deuterochloroform. Guest signals (filled circles): H_1_ (orange), H_2_ (green), H_3_ (blue), H_4_ (red), H_5_ (pale blue). Host signals (open circles): H_a_ (purple), H_b_ (magenta), H_c_ (black), H_d_ (grey), H_e_ (brown), H_f_ (cyan).

The ^1^H NMR spectra of the complexes provide a sensitive probe of the geometries of the aromatic interactions in these systems, because there are large complexation-induced changes in chemical shift (Δ*δ*) associated with the ring currents of the aromatic rings. Tables S3 and S4† report the Δ*δ* values measured for the complexes formed by the super-aryl-extended calix[4]pyrroles 1 and 2 and the eleven different heterocyclic guests in deuterium oxide and deuterochloroform. Fig. 7 compares the chemical shift changes measured for the heterocyclic complexes with the results for the PPNO complex.

**Fig. 8:**
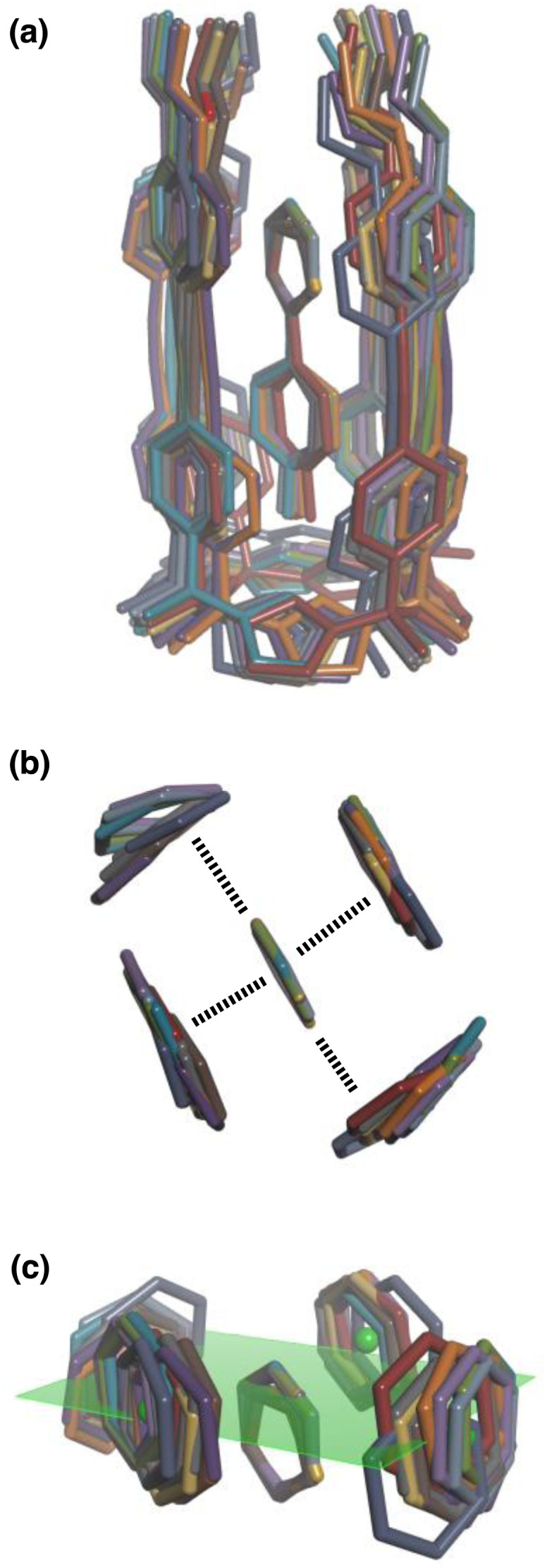
Overlay of the three-dimensional structures of complex A of the DMC with the 11 different guests using Discovery Studio Visualizer v21.1.0.2098, Dassault Sistèmes Biovia Corp. https://discover.3ds.com/discovery-studio-visualizer-download. (a) Complete structure of the complex. (b) Top view of the geometry of the two stacking and two edge-to-face interactions measured by the DMC. (c) Side view of the geometry of the two stacking and two edge-to-face interactions measured by the DMC. The green dots are the centroids of the aromatic rings on the walls of the cavity, and the plane through these points is shown in green. The centres of the guest aromatic rings lie slightly below this plane. The energy minimized structures of the complexes were obtained at the RI-BP6-D3BJ-def2-TZVP level of theory as implemented in TURBOMOLE v7.0 2015, a development of University of Karlsruhe and Forschungszentrum Karlsruhe GmbH, 1989–2007, TURBOMOLE GmbH, since 2007; available from http://www.turbomole.com.^26–35^

The Δ*δ* values for the guest are remarkably consistent from one complex to another in both water and chloroform. The large negative values of Δ*δ* measured for the protons on the heterocyclic rings (−1 to −2 ppm) confirm that these groups lie over the faces of the green aromatic rings at the top of the host binding pocket, as illustrated in Fig. 1 and 2. Fig. 8(b) shows that Δ*δ* values measured for the host do not vary significantly for different guests in deuterochloroform. In deuterium oxide, the ^1^H NMR signals of the free host were broad, so it was not possible to determine complexation-induced changes in chemical shift. However, the spectra of the complexes were sharp and well-resolved, and the chemical shifts of the signals due to the bound host are practically identical for all complexes in deuterium oxide (see Table S5†). The NMR data suggest that structures of the complexes do not vary significantly from one guest to another and that the geometrical arrangement of aromatic rings that interact with the heterocycles is similar in all of the complexes.

This conclusion is supported by Density Functional Theory (DFT) calculations. Fig. 8(a) shows a side view of an overlay of the minimum energy structures of complex A calculated for each of the 11 guests, and Fig. 8(b) shows a top view of the two edge-to-face and two stacking interactions that are measured by the DMC. The calix[4]pyrrole framework locks the guests into a very well-defined binding pocket, and there is no significant change in the geometry of the aromatic interactions when the heterocycle is varied.

## Discussion

**Fig. 9:**
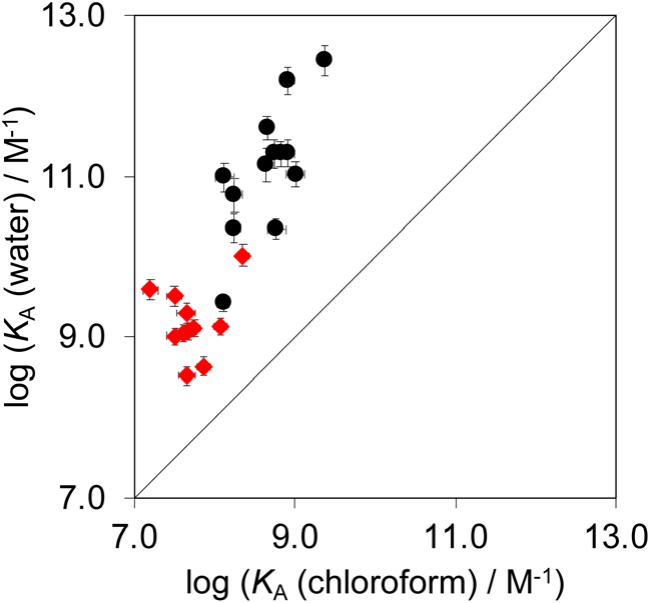
Association constants for formation of complex A of the DMC measured in water compared with the corresponding values measured in chloroform at 298 K. The red points are the heterocycle measurements described here, and the black points are the phenyl derivatives reported previously.^22^ The line corresponds to *y* = *x*.

Complex A which features the aromatic interactions with the heterocyclic ring on the guest is orders of magnitude more stable than complexes B, C and D of the DMC in all cases. The red datapoints in Fig. 9 compare the association constants for formation complex A in water with the corresponding values measured in chloroform. Data for the previously reported measurements on the complexes formed with phenylpyridine *N*-oxide derivatives are included for comparison (black points). The heterocyclic guests bind with an affinity that is roughly two orders of magnitude lower than the phenyl guests in water and one order of magnitude lower in chloroform. However, in all cases the association constants measured in water are significantly higher than the values measured in chloroform. These results indicate that the aromatic interactions made with the heterocyclic rings are favourable in both water and chloroform and lead to a significant stabilisation of complex A, but aromatic interactions with heterocycles are less favourable than interactions with aromatic hydrocarbons. In addition, the solvation/desolvation processes that occur in water lead to a significant stabilisation of aromatic interactions with the heterocyclic rings relative to the organic solvent.

**Fig. 10:**
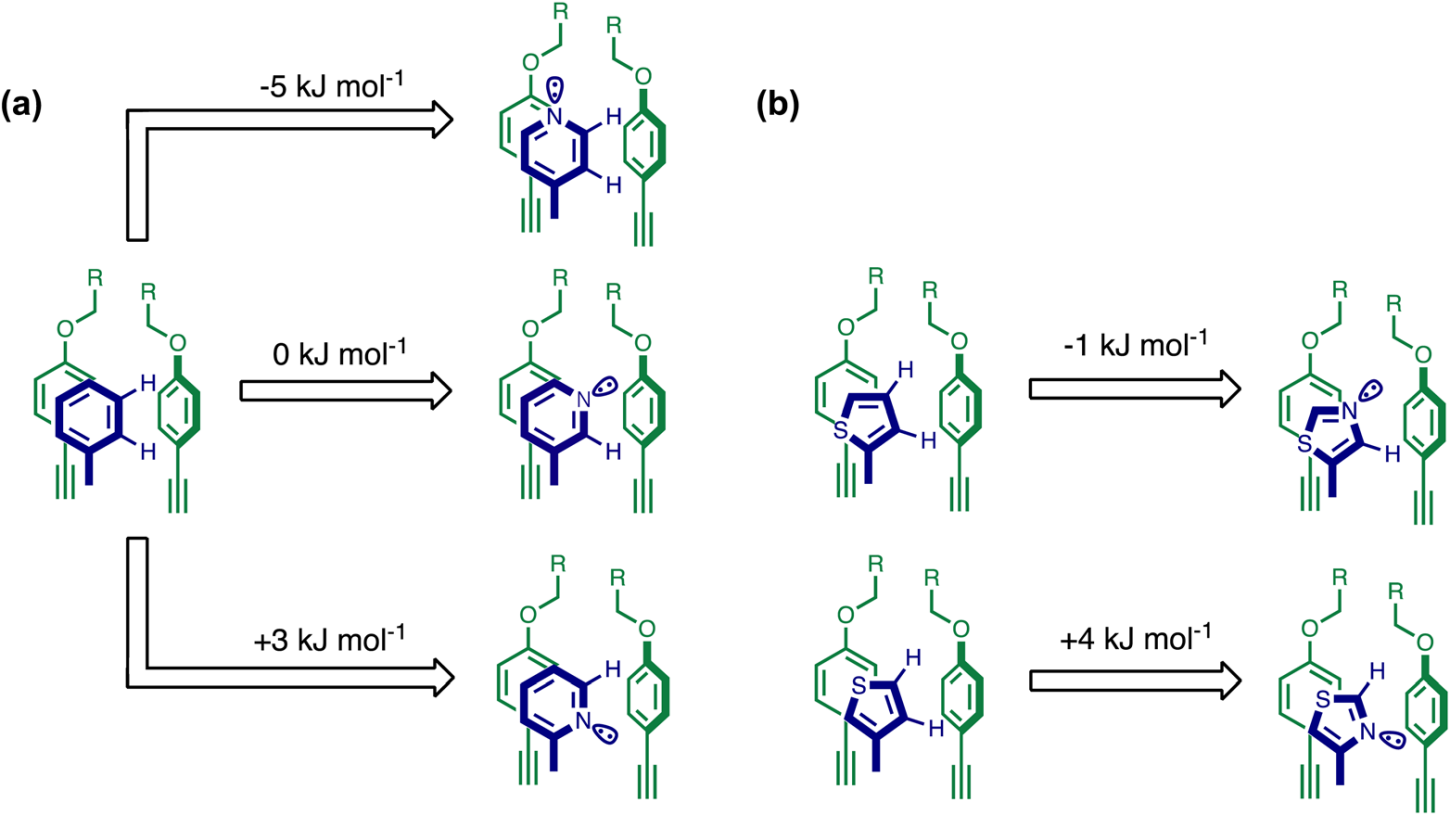
The effect of heterocyclic nitrogen atoms on the free energy contribution due to aromatic interactions in complex A (ΔΔ*G*°). The change in ΔΔ*G*° associated with introducing (a) a pyridine nitrogen compared with a phenyl group and (b) a thiazole nitrogen compared with a thiophene group. Only one stacking and one edge-to-face interaction are shown for clarity, but the free energy differences are measured for the sum of four aromatic interactions.

Using the data in Tables S1 and S2† and eqn (1), the total free energy contribution (ΔΔ*G*°/kJ mol^−1^) due to the four heterocycle–aromatic interactions in complex A was determined in water and in chloroform. The results are reported in Table 1. All interactions are attractive. The values of ΔΔ*G*° in water range from −9 kJ mol^−1^ to −18 kJ mol^−1^ and are more significantly attractive than the corresponding values measured in chloroform (−2 kJ mol^−1^ to −11 kJ mol^−1^). The measurements made in chloroform provide insights into the effect of heteroatoms on aromatic interactions without the complications of solvophobic effects. Fig. 10(a) illustrates the effect of introducing a pyridine nitrogen atom on the aromatic interactions made with the walls of the binding site in the receptor. The value of ΔΔ*G*° measured when 4-phenylpyridine *N*-oxide (PPNO) was used as the guest serves as a reference point for comparison with the results obtained for other heterocycles. Introduction of a nitrogen in the 4-position on the guest (5) makes the aromatic interactions more favourable by −5 kJ mol^−1^. Fig. 10(a) shows that the pyridine nitrogen lone pair points out of the binding site and does not make any contacts with the aromatic side-walls of the receptor. The CH groups on the pyridine ring are more positive than the CH groups on the phenyl group of PPNO, and the improved electrostatic interactions with the π-electron density on the faces of the receptor side-walls are presumably responsible for the more favourable aromatic interactions in this system.

**Table 1:** Free energy contributions (ΔΔ*G*°/kJ mol^−1^) to the stability of complex A of the DMC due to the four heterocycle–aromatic interactions

Guest	R	Solvent	
		Water	Chloroform
PPNO	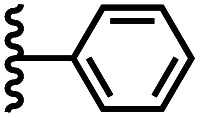	−13.0 ± 0.8	−6.3 ± 0.8
5	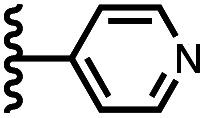	−17.8 ± 0.7	−10.9 ± 1.0
6	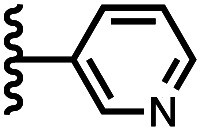	−12.8 ± 0.8	−6.1 ± 1.5
7	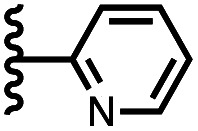	−13.8 ± 0.8	−3.0 ± 0.6
8	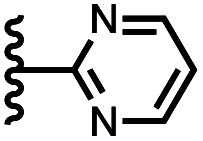	−14.6 ± 0.8	−2.1 ± 0.8
9	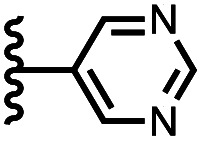	−12.3 ± 0.8	−6.6 ± 1.0
10	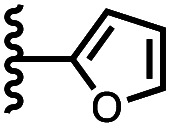	−8.7 ± 1.3	−5.6 ± 0.8
11	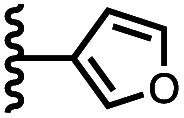	−9.4 ± 0.6	−6.1 ± 0.8
12	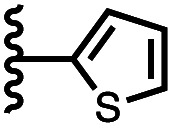	−11.8 ± 1.1	−5.7 ± 1.8
13	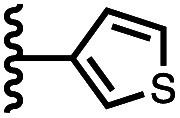	−12.2 ± 0.8	−8.3 ± 1.3
14	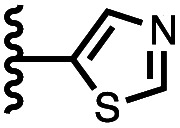	−12.4 ± 1.4	−6.3 ± 0.7
15	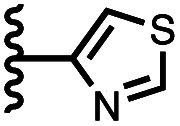	−11.7 ± 0.8	−4.8 ± 0.8

When the pyridine nitrogen is moved from the 4-position to the 3-position on the guest (6), the nitrogen lone pair points towards the π-face of the receptor side-walls. The aromatic interaction measured for this system is the same as observed for PPNO, which means that the additional stability conferred by the more polar pyridine CH groups is compensated by the repulsive interaction between the pyridine lone pair of the guest and the π-electron density on the side-walls of the receptor. When the pyridine nitrogen is moved to the 2-position on the guest (7), the aromatic interactions are 3 kJ mol^−1^ less favourable than observed for PPNO. This result suggests that the geometry of this complex forces the pyridine lone pair to make a more repulsive interaction with the π-face of the receptor. A similar analysis can be carried out for five-membered heterocyclic guests by comparing the results for thiazole guests 14 and 15, which contain a pyridine-type nitrogen with a lone pair, and the thiophene guests 12 and 13, which do not (Fig. 10(b)). The results are very similar: a nitrogen lone-pair that points towards a π-face of the receptor at the top of the cavity makes less repulsive interactions than a lone pair that points towards a π-face at the lower end of the cavity.

In contrast, introduction of oxygen or sulphur in the furan and thiophene guests has almost no effect on the aromatic interactions, when compared with value of ΔΔ*G*° measured for the phenyl group in 4-phenylpyridine *N*-oxide (PPNO). The presence of the heteroatoms affects the polarity of the CH groups and the π-electron density on the face of the heterocycles, but any effects on the edge-to-face and stacking interactions with the receptor appear to cancel out. Neither oxygen nor sulphur are strong H-bond acceptors, so the lone pair repulsion observed for nitrogen heterocycles is not observed for these systems.

**Fig. 11:**
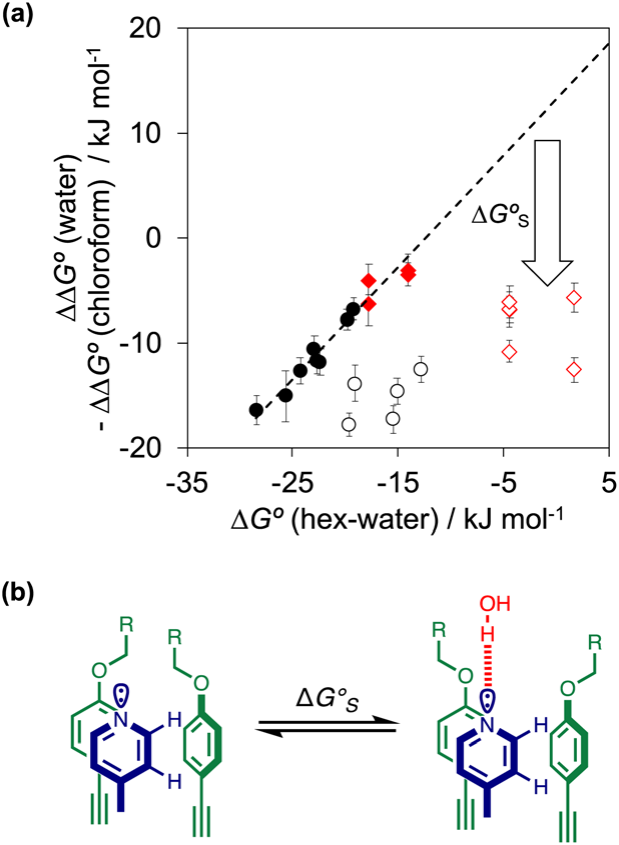
(a) The difference between the water and chloroform DMC measurements of the free energy contributions due to aromatic interactions in complex A of the DMC (ΔΔ*G*°) compared with the free energy of transfer of the corresponding benzene derivative or heterocycle from water into *n*-hexadecane (Δ*G*°(hex–water)). Non-polar phenyl derivatives are plotted as filled black points, polar phenyl derivatives as open black points, furan and thiophene as filled red points, and pyridine, pyrimidine and thiazole as open red points. The line of best fit shown for non-polar phenyl derivatives is *y* = 1.1*x* + 13.7 kJ mol^−1^ (*R*^2^ = 0.97). (b) The additional free energy contribution 
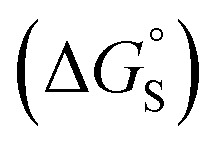
 is due to resolvation of the polar nitrogen atoms by water in the complex.

As we have shown previously, the aromatic interactions made by the phenyl group of PPNO are more favourable in water than in chloroform due to the hydrophobic effect.^22^ The hydrophobic contribution to the observed binding affinity in water can be estimated using experimental measurements of the free energy of transfer of the corresponding benzene derivatives from water into *n*-hexadecane (Δ*G*°(water–hex)). Fig. 11(a) shows that the difference between the values of ΔΔ*G*° measured in water and in chloroform for a range of phenylpyridine *N*-oxide guests correlates linearly with Δ*G*°(water–hex) (filled black points). The slope of this correlation is one, which confirms that the difference between the two solvents is due to desolvation of the non-polar phenyl derivatives in water. Fig. 11(a) also shows the data for the heterocylic guests. For the furan (10 and 11) and thiophene (12 and 13) guests, the results fall on the same trendline as the non-polar phenyl derivatives (filled red points). These heterocycles are relatively non-polar, and the more favourable aromatic interactions observed in water can therefore be understood simply in terms of the hydrophobic effect, which is quantified by Δ*G*°(water–hex).

Different behaviour was observed for the nitrogen heterocycles, which are plotted as open red points in Fig. 11(a). According to the trendline in Fig. 11(a), these heterocycles should make less favourable aromatic interactions in water than in chloroform, but the data show that the opposite is true. The value of Δ*G*°(water–hex) includes an energy penalty for desolvation of the polar nitrogen atoms that make strong H-bonding interactions with water. If these nitrogen atoms are in fact solvated by water in the complex formed with the super-aryl-extended calix[4]pyrrole, then the measured values of ΔΔ*G*° will be more favourable by an amount Δ*G*°_S_, which we define as the resolvation energy (Fig. 11(b)). Similar behaviour was observed previously for polar phenyl derivatives, which are plotted as open black points in Fig. 11(a). The non-polar alkoxy substituents on the upper rim of the binding pocket either fold over non-polar groups to maximise the desolvation of the guest or rotate away from the pocket to allow water to solvate polar groups on the guest, as illustrated in Fig. 11(b). The corresponding resolvation energies for the polar heterocyclic guests are reported in Table 2.

**Table 2:** Stabilisation of complex A in water due to resolvation of polar groups

Guest	R	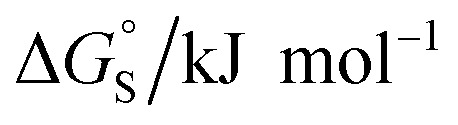
5	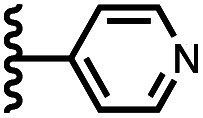	−15
6	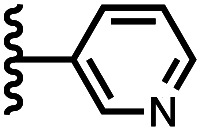	−15
7	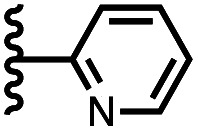	−19
8	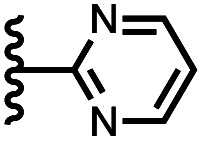	−28
9	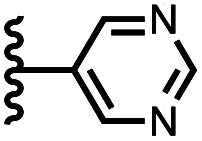	−21
14	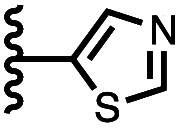	−15
15	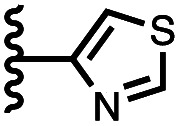	−15

The resolvation energies are −15 kJ mol^−1^ for most of the heterocycles, regardless of the location of the nitrogen H-bond acceptor. This result suggests that the binding pocket has sufficient flexibility to allow water to access one edge of the heterocycle, no matter which direction the nitrogen lone pair points, directly into solvent at the top of the cavity (5), towards the side at the top of the cavity (6 and 14), or towards the side at the bottom of the cavity (7 and 15). As might be expected, the resolvation energies are significantly more favourable for the pyrimidines, which have two nitrogen atoms that can both make H-bonds to the solvent.

**Fig. 12:**
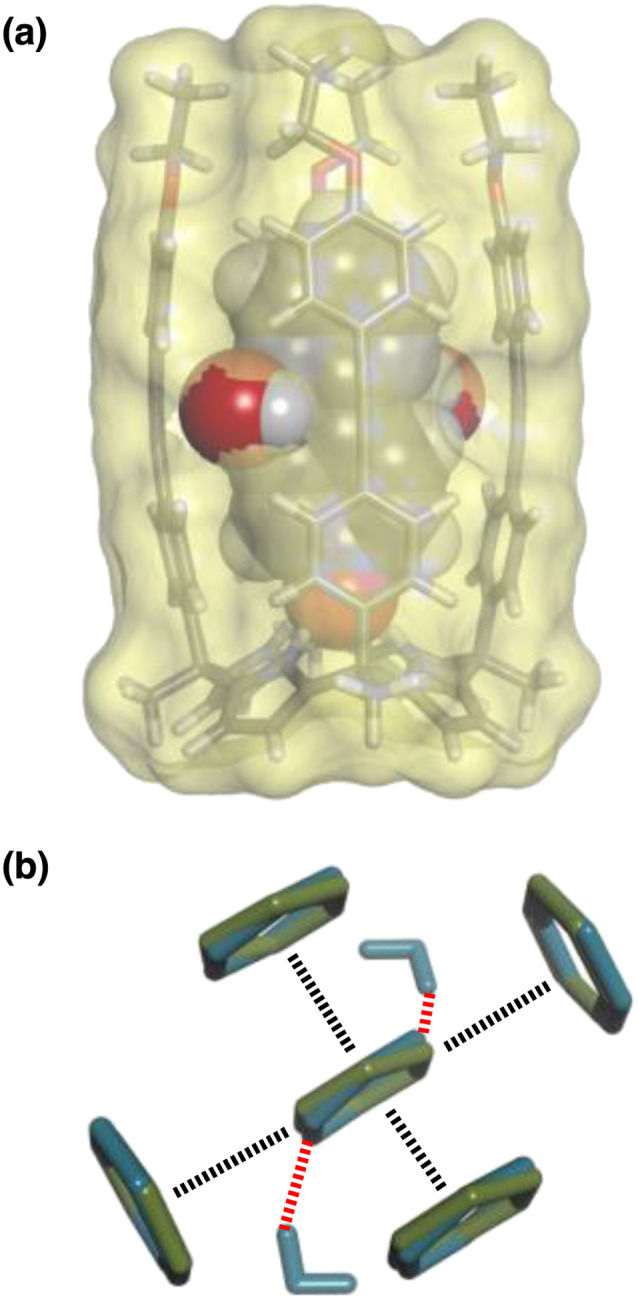
(a) Three-dimensional structures of the 1·8 complex optimised with two molecules of water using Discovery Studio Visualizer v21.1.0.2098, Dassault Sistèmes Biovia Corp. https://discover.3ds.com/discovery-studio-visualizer-download. (b) Top view of an overlay of the four aromatic interactions in the 1·8 complex in the presence (blue) and absence (green) of two water molecules. The energy minimized structures of the complexes were obtained at the RI-BP6-D3BJ-def2-TZVP level of theory as implemented in TURBOMOLE v7.0 2015, a development of University of Karlsruhe and Forschungszentrum Karlsruhe GmbH, 1989–2007, TURBOMOLE GmbH, since 2007; available from http://www.turbomole.com.^26–35^

The accessibility of guests bound in the receptor to water was investigated using DFT calculations including explicit solvent. Fig. 12(a) shows the structure of the complex formed with pyrimidine 8 and two water molecules. The two pyrimidine nitrogen atoms are involved in H-bonding interactions with the two water molecules, which appear to be able to access the binding pocket without significant distortion of the receptor. Fig. 12(b) compares the geometry of the aromatic interactions between the pyrimidine ring and the side walls of the receptor in the presence and absence of the water molecules. The two structures are practically identical, which confirms that resolvation is the most likely explanation for the results in Fig. 11(a), because there is sufficient space at the edges of the binding pocket for water to solvate the polar nitrogen atoms in the host·guest complexes.

## Conclusions

The 1:1 complex formed by a super-aryl-extended calix[4]pyrrole and a pyridine *N*-oxide guest represents an excellent platform for quantitative measurements of non-covalent interactions between different functional groups in different solvents. The three-dimensional structures of the complexes are fixed by four H-bonding interactions between the pyrrole donors at the bottom of the receptor and the *N*-oxide acceptor on the guest, locking the geometrical arrangement of interacting functional groups in the binding pocket at the other end of the receptor. In the systems described here, an aromatic heterocycle on the guest makes two stacking interactions and two edge-to-face interactions with the side walls of the receptor. Chemical double mutant cycles were used to measure the free energy contribution of these four aromatic interactions to overall stability of the complex for 11 different heterocycles. The solubility of the receptor can be controlled using substituents that are remote from the binding site, allowing measurement of the same non-covalent interactions in water and in chloroform.

The three-dimensional structures of the complexes were characterised using NMR spectroscopy. Complexation-induced changes in ^1^H NMR chemical shift show that the structure of the complex does not change as the nature of the heterocycle on the guest is varied, and these results are corroborated by DFT calculations. The dissociation constants for formation of the complexes were all in the nanomolar range, as determined by ITC and NMR competition experiments, and these data form the basis of the DMC measurements of the free energy contributions due to the aromatic interactions.

In chloroform, the effects of introducing a pyridine nitrogen depend on the geometry with respect to the aromatic side-walls of the cavity. When the nitrogen lone pairs points into the solvent, the free energy changes associated with the aromatic interactions are 5 kJ mol^−1^ more favourable than the corresponding aromatic hydrocarbon interaction, reflecting the increase in the polarity of the hydrogen atoms that interact with the π-faces of the binding site. When the nitrogen lone pair points towards a π-face of the binding site, repulsive interactions worth 5–8 kJ mol^−1^ are introduced. Similar behaviour was observed for interactions with pyrimidine and thiazole heterocycles. However, aromatic interactions with furan and thiophene were very similar in magnitude to those observed for phenyl, regardless of the orientation of the lone pairs, which are much less polar than a nitrogen lone pair.

The heterocycle aromatic interactions are all significantly more favourable in water than in chloroform (by up to 12 kJ mol^−1^). For the non-polar heterocycles, furan and thiophene, the increase in interaction energy correlates directly with hydrophobicity, as measured by the free energy of transfer of the heterocycle from *n*-hexadecane into water (Δ*G*°(water–hex)). For the heterocycles with polar nitrogen H-bond acceptors, the aromatic interactions are significantly more favourable than expected based on the values of Δ*G*°(water–hex). Molecular models indicate that water can access cracks in the walls of receptor binding site to solvate the edges of the heterocycles without significantly affecting the geometry of the aromatic interactions. Thus, the polar heterocycles enjoy the best of both worlds: the non-polar parts of the heterocycle are removed from the solvent and make aromatic interactions with the receptor, which leads to a stabilisation due to hydrophobic desolvation; the polar parts of the heterocycle are able to maintain H-bonding interactions with water, so that there is no desolvation penalty when they enter the receptor binding site.

These experiments reveal the factors that are important for understanding the contribution of amphiphilic heterocycles to binding affinity in more complicated systems. Partial desolvation can allow non-polar parts of the heterocycle maximise hydrophobic contributions to binding affinity, while polar sites maintain interactions with the solvent. The result for the systems described here is that all types of aromatic interaction are significantly enhanced in water compared with chloroform due to the hydrophobic effect, no matter how polar the heterocycle is.

## Data availability

All supporting data is provided in the ESI.†

## Author contributions

The manuscript was written through contributions of all authors.

## Conflicts of interest

There are no conflicts to declare.

## Supplementary Material

SC-014-D3SC03824F-s001
