# Supplementary material for: Aromatic interactions with heterocycles in water
Source: Chem Sci. 2023 Sep 29;14(40):11131–40. doi: 10.1039/d3sc03824f (PMC10583712; doi:10.1039/d3sc03824f)
Supplement: SC-014-D3SC03824F-s001 [file SC-014-D3SC03824F-s001.pdf]

Electronic Supplementary Material (ESI) for Chemical Science.
This journal is © The Royal Society of Chemistry 2023

Aromatic Interactions with Heterocycles in Water

Gloria Tobajas-Curiel,[1] Qingqing Sun,[2,3] Jeremy K. M. Sanders,[1] Pablo Ballester,[2,4,*] Christopher A. Hunter[1,*]

[1]*Yusuf Hamied Department of Chemistry, University of Cambridge, Cambridge CB2 1EW, U.K. Email: herchelsmith.orgchem@ch.cam.ac.uk*

[2] *Institute of Chemical Research of Catalonia (ICIQ), Barcelona Institute of Science and Technology (BIST), Av. Països Catalans, 16, 43007, Tarragona, Spain. Email: pballester@iciq.es*

[3]*Yangzhou University, School of Chemistry and Chemical Engineering, Yangzhou, 225002 Jiangsu (China). Email: sunqingqing@snnu.edu.cn.*

[4]*ICREA, Passeig Lluís Companys 23, 08010, Barcelona, Spain.*

Supporting Information

1. General methods 3

2. Chemistry procedures 4

   2.1. General experimental procedure for the synthesis of **5**, **6**, **9-14**: 4

   2.2. General experimental procedure for the synthesis of **16**, **17** and **18**: 4

   2.3. General experimental procedure for the synthesis of **7**, **8** and **15**: 4

   2.4. Physical data of **5-18**: 5

3. Isothermal titration calorimetry (ITC) experiments 33

   3.1. Octapyridinium-super-aryl-extended calix[4]pyrrole **1** 33

   3.2. Tetrapyridinium-aryl-extended calix[4]pyrrole **3** 34

   3.3. Octachloro-super-aryl-extended calix[4]pyrrole **2** 40

   3.4. Tetrachloro-aryl-extended calix[4]pyrrole **4** 41

4. Pair-wise ^{1}H NMR competitive titrations 47

4.1. Octapyridinium-super-aryl-extended calix[4]pyrrole **1** 47

4.2. Octachloro-super-aryl-extended calix[4]pyrrole **2** 53

5. Analysis of binding 59

6. Analysis of structures 61

7. References 64

1. General methods

All the reagents and materials used in the synthesis of the compounds described below were obtained from commercial sources and used without prior purification. Compounds **1-4** were prepared as reported in literature.[1–3] Thin layer chromatography was carried out using Silica gel 60F on glass plates. Flash chromatography was carried out on an automated system (Combiflash Companion, Combiflash Rf+ or Combiflash Rf Lumen) using prepacked cartridges of silica (25 μm or 50μm PuriFlash® Columns). ^{1}H and ^{13}C NMR spectra were recorded on Bruker 400 MHz DPX400, 400 MHz AVIII400, 500 MHz DCH cryoprobe or 500 MHz TCI Cryoprobe spectrometer at 298.0 ± 0.1 K unless specifically stated otherwise. Residual solvent was used as an internal standard for referencing. In chloroform-*d*, ^{1}H spectra were referenced to δ 7.26 ppm and ^{13}C spectra to δ 77.06 ppm for the solvent signal. In dimethyl sulfoxide-d_6, ^{1}H spectra were referenced to δ 2.50 ppm and ^{13}C spectra to δ 39.52 ppm. In deuterium oxide, ^{1}H spectra were referenced to δ 4.79 ppm. All chemical shifts are quoted in ppm on the δ scale and the coupling constants expressed in Hz. Signal splitting patterns are described as follows: s (singlet), br s (broad singlet), d (doublet), t (triplet), q (quartet), m (multiplet). FT-IR spectra were recorded on a PerkinElmer Spectrum One FT-IR spectrometer equipped with an ATR cell. The LCMS analysis of samples was performed using a Waters Acquity H-Class UPLC coupled with a single quadrupole Waters SQD2 or a Waters Xevo G2-S bench top QTOF machine. Melting points were measured on a Mettler Toledo MP90 melting point apparatus. ITC titrations were carried out on a Malvern MicroCal VP-ITC MicroCalorimeter.

2. Chemistry procedures

2.1. General experimental procedure for the synthesis of 5, 6, 9-14:

To a solution of 4-chloro pyridine *N*-oxide (1 equivalent) in dioxane (4 mL) and under nitrogen, the corresponding boronic acid or boronic acid pinacol ester (2 equivalents), palladium tetrakis triphenylphospine (0.05 equivalents) and aqueous solution of sodium carbonate (2 M, 4 equivalents) were added. The mixture was stirred at 80 °C for 16 h. Ethyl acetate (20 mL) and NaOH 1M (5 mL) were then added. The organic and aqueous layers were separated and the aqueous layer was extracted with ethyl acetate (3 x 20 mL). The combined organic layers were dried over anhydrous $MgSO_4$, and the solvents evaporated in vacuo. The crude product was purified by flash column chromatography on silica gel (methanol in dichloromethane 0 to 15 % in 20 minutes for **5**, **6**, **9** and **14**, and 0 to 5 % in 20 minutes for **10-13**) to yield the corresponding pyridine *N*-oxide derivatives **5**, **6**, **9-13**.

2.2. General experimental procedure for the synthesis of 16, 17 and 18:

To a solution of pyridine 4-boronic acid (1.5 equivalents) in dioxane (4 mL) and under nitrogen, the corresponding bromo-derivative (1 equivalent), palladium tetrakis triphenylphospine (0.05 equivalents) and aqueous solution of sodium carbonate (2 M, 4 equivalents) were added. The mixture was stirred at 80 °C for 16 h. Ethyl acetate (20 mL) and NaOH 1M (5 mL) were then added. The organic and aqueous layers were separated and the aqueous layer was extracted with ethyl acetate (3 x 20 mL). The combined organic layers were dried over anhydrous $MgSO_4$, and the solvents evaporated in vacuo. The crude product was purified by flash column chromatography on silica gel (methanol in dichloromethane 0 to 5 % in 20 minutes) to yield the corresponding pyridine derivatives **16**, **17** and **18**.

2.3. General experimental procedure for the synthesis of 7, 8 and 15:

A solution of the corresponding intermediates **16**, **17** or **18** (1 equivalent) in anhydrous dichloromethane (1.5 mL) was cooled to 0 ºC in an ice-water bath. mCPBA (1.1 equivalents) was added at 0º C and the mixture was allowed to warm to room temperature and stirred for 16 h. The crude of the reaction was purified by flash column chromatography on silica gel (methanol in dichloromethane 0 to 10 % in 20 minutes) to yield the corresponding pyridine *N*-oxide derivatives **7**, **8** and **15**.

2.4. Physical data of 5-18:

[4,4'-Bipyridine] 1-oxide (5)

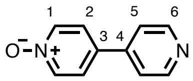

White solid (62 mg, 0.36 mmol, 58 %)

Mpt: 176.5 – 178.4 °C

^{1}H NMR (400 MHz, DMSO-d$_6$): δ_H 8.68 (d, J = 6.3 Hz, 2H, H-6), 8.34 (d, J = 7.3 Hz, 2H, H-1), 7.93 (d, J = 7.3 Hz, 2H, H-2), 7.81 (d, J = 6.2 Hz, 2H, H-5)

^{13}C NMR (101 MHz, DMSO-d$_6$): δ_C 150.5 (C-6), 142.6 (C-4), 139.3 (C-1), 132.8 (C-3), 124.1 (C-2), 120.4 (C-5).

HRMS (ES+): calculated for $C_{10}H_8N_2O$ 173.0709 [M+H$^+$], found 173.0717 [M+H$^+$].

FT-IR (ATR): ν_{max} 3043, 1592, 1474, 1409, 1263, 1232, 1187, 1025, 814, 733, 574 cm^{-1}.

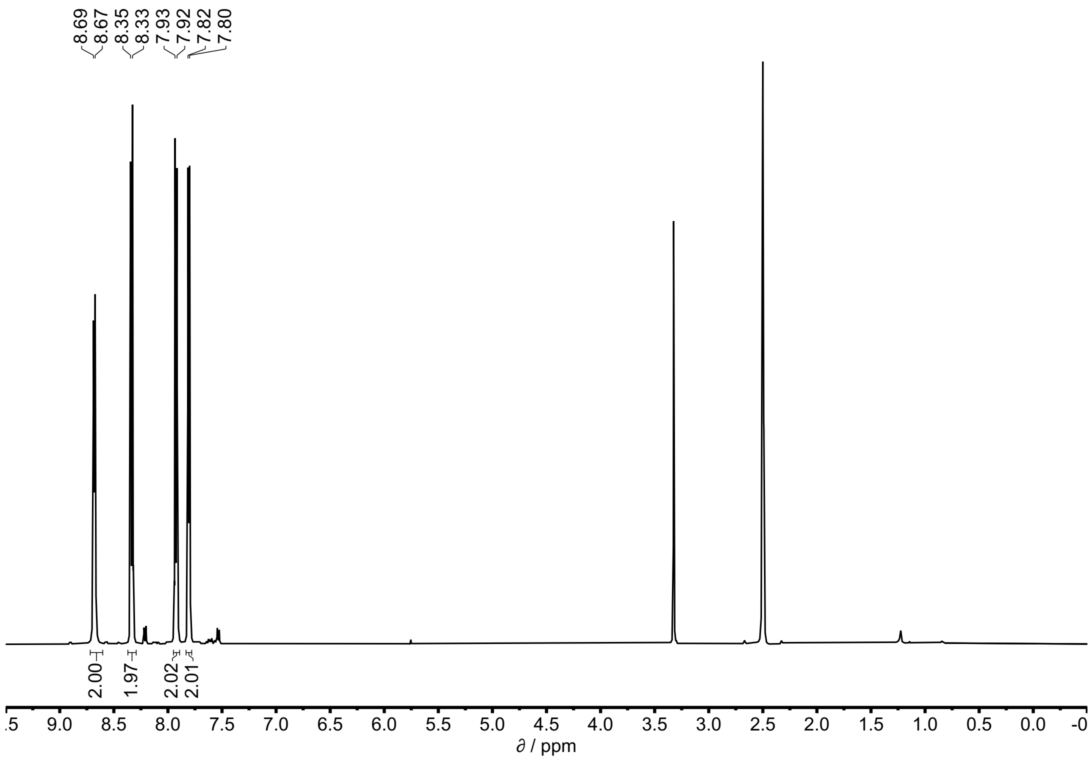

Figure S1. *¹H NMR spectrum (400 MHz, DMSO-d₆) of compound* ***5****.*

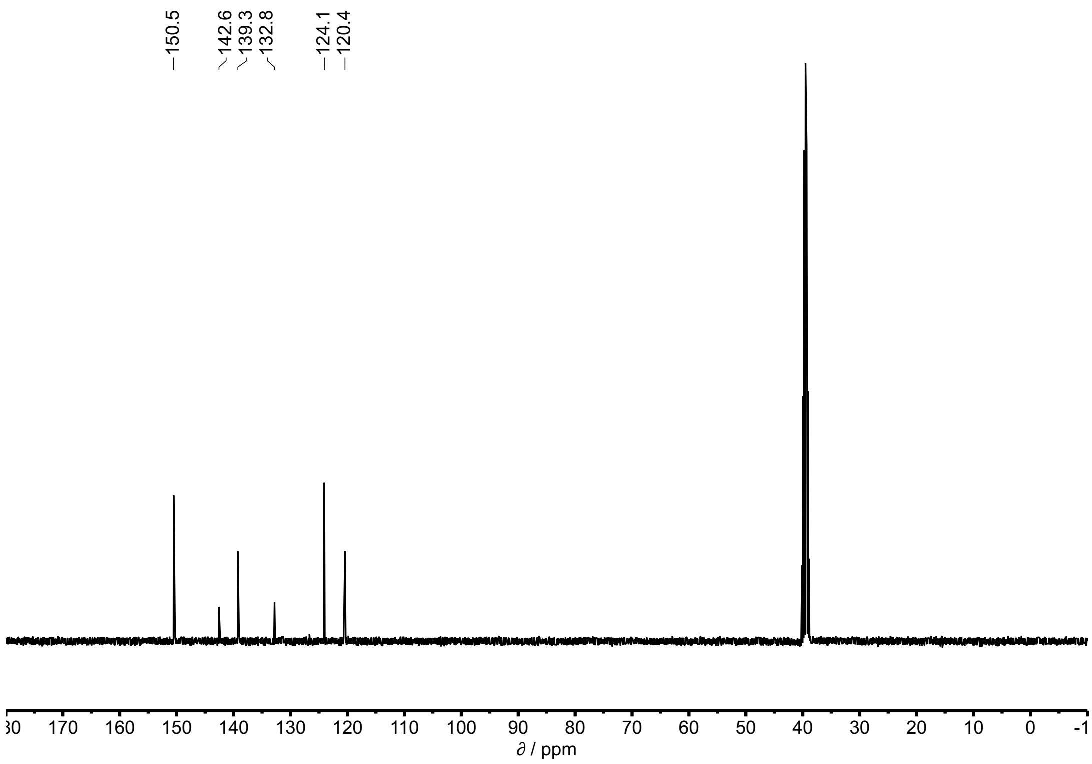

Figure S2. *¹³C NMR spectrum (101 MHz, DMSO-d₆) of compound* ***5****.*

[3,4'-Bipyridine] 1-oxide (6)

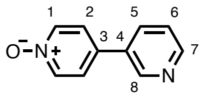

White solid (28 mg, 0.16 mmol, 45 %)

Mpt: 131.3 – 133.3 °C

^{1}H NMR (400 MHz, DMSO-d$_6$): δ_H 9.01 (d, *J* = 1.7 Hz, 1H, H-8), 8.62 (dd, *J* = 4.8, 1.6 Hz, 1H, H-7), 8.31 (d, *J* = 7.2 Hz, 2H, H-1), 8.23 – 8.16 (m, 1H, H-5), 7.86 (d, *J* = 7.3 Hz, 2H, H-2), 7.57 – 7.45 (m, 1H, H-6).

^{13}C NMR (101 MHz, DMSO-d$_6$): δ_C 149.5 (C-7), 147.3 (C-8), 139.1 (C-1), 133.7 (C-5), 133.1 (C-3), 131.4 (C-4), 124.0 (C-2, C-6).

HRMS (ES+): calculated for $C_{10}H_8N_2O$ 173.0709 [M+H$^+$], found 173.0714 [M+H$^+$].

FT-IR (ATR): ν_{max} 3030, 1473, 1452, 1403, 1252, 1182, 848, 815, 798, 704, 625, 584 cm^{-1}.

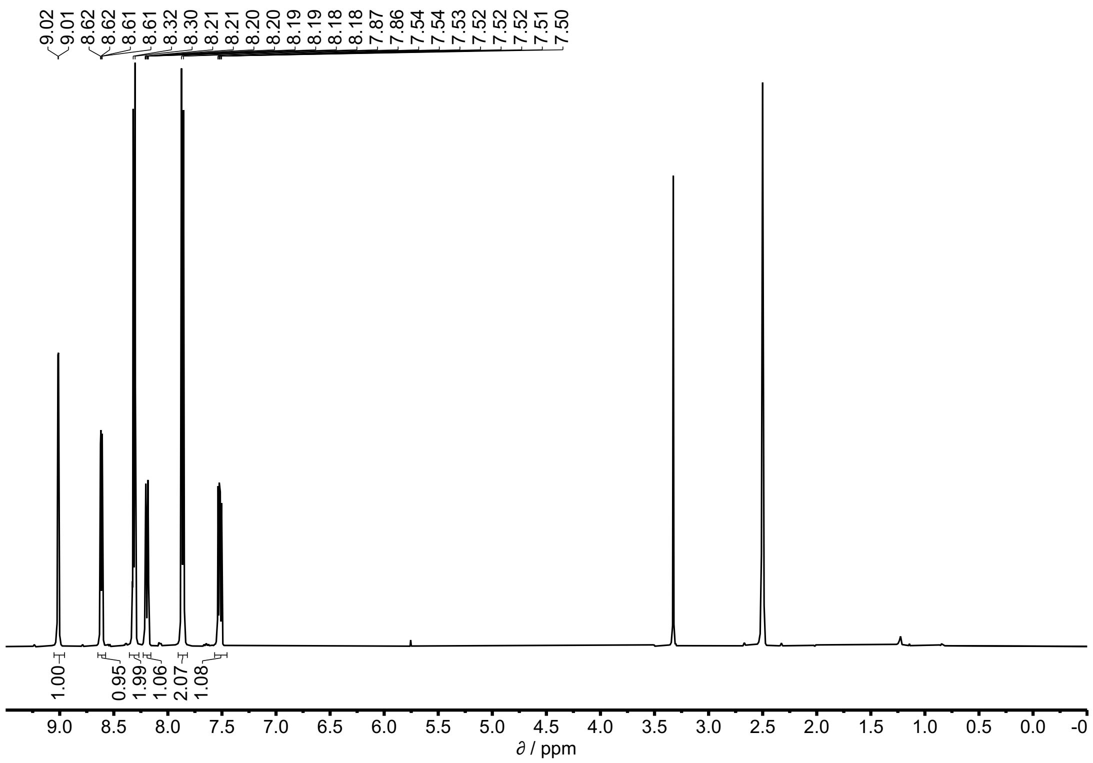

Figure S3. *^{1}H NMR spectrum (400 MHz, DMSO-d$_6$) of compound **6**.*

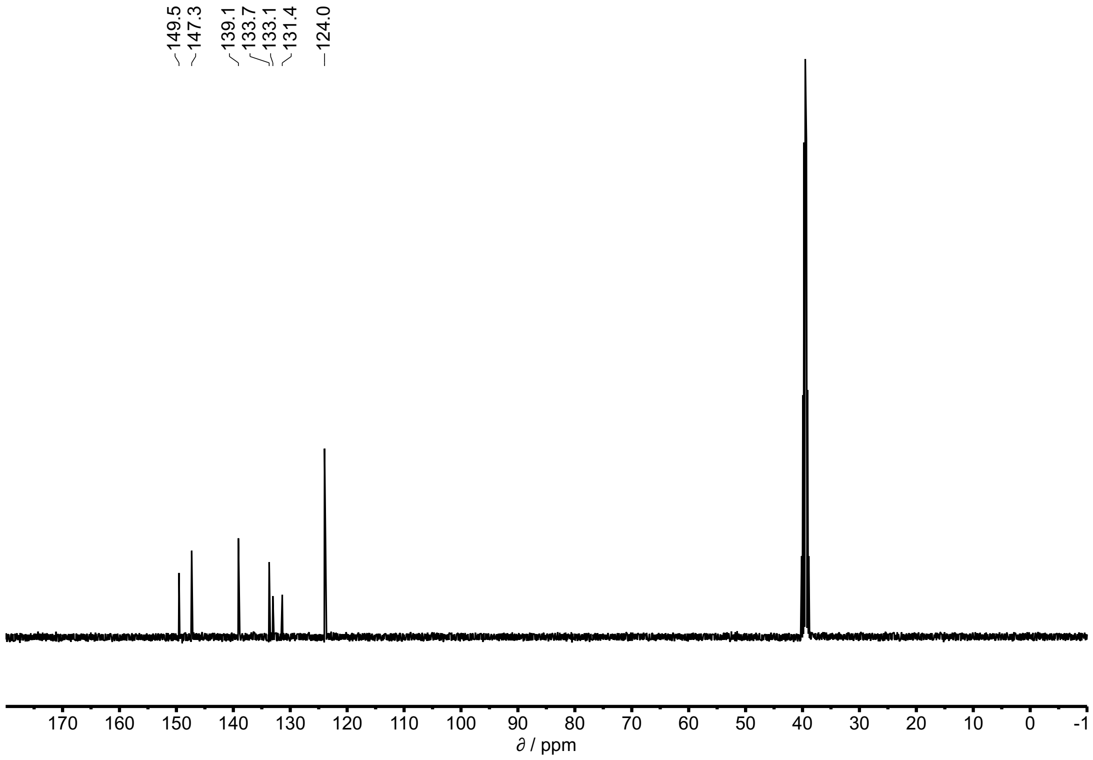

Figure S4. *^{13}C NMR spectrum (101 MHz, DMSO-d$_6$) of compound **6**.*

[2,4'-Bipyridine] 1'-oxide (7)

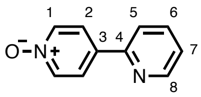

Light brown solid (10 mg, 0.06 mmol, 36 %).

Mpt: 116.2 – 118.2 °C

^{1}H NMR (400 MHz, CDCl$_3$): δ_H 8.73 (d, *J* = 4.4 Hz, 1H, H-8), 8.35 (d, *J* = 7.3 Hz, 2H, H-1), 8.02 (d, *J* = 7.2 Hz, 2H, H-2), 7.85 (td, *J* = 7.7, 1.8 Hz, 1H, H-6), 7.77 (d, *J* = 8.0 Hz, 1H, H-5), 7.38 – 7.33 (m, 1H, H-7).

^{13}C NMR (101 MHz, CDCl$_3$): δ_C 152.2 (C-4), 150.2 (C-8), 139.6 (C-1), 137.6 (C-6), 124.0 (C-7), 123.7 (C-2), 120.5 (C-5).

HRMS (ES+): calculated for $C_{10}H_8N_2O$ 173.0709 [M+H$^+$], found 173.0713 [M+H$^+$].

FT-IR (ATR): ν_{max} 3062, 2918, 1589, 1468, 1445, 1426, 1247, 1175, 1030, 850, 779, 737, 588 cm^{-1}.

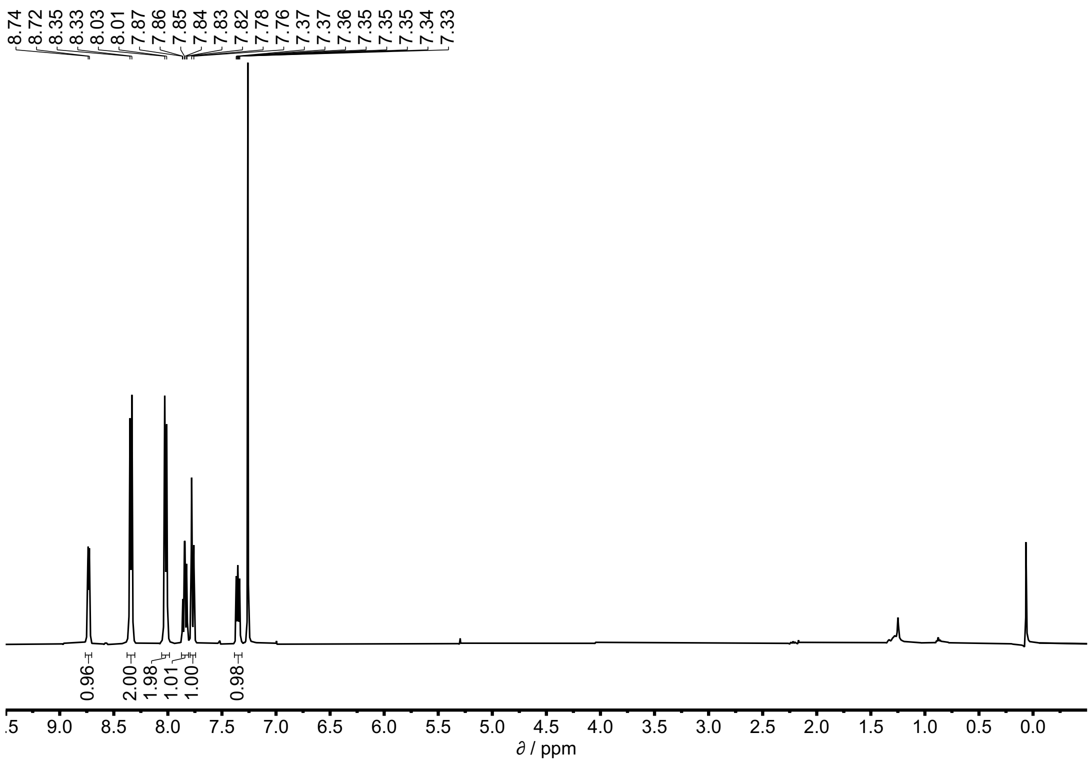

Figure S5. *^{1}H NMR spectrum (400 MHz, $CDCl_3$) of compound* ***7****.*

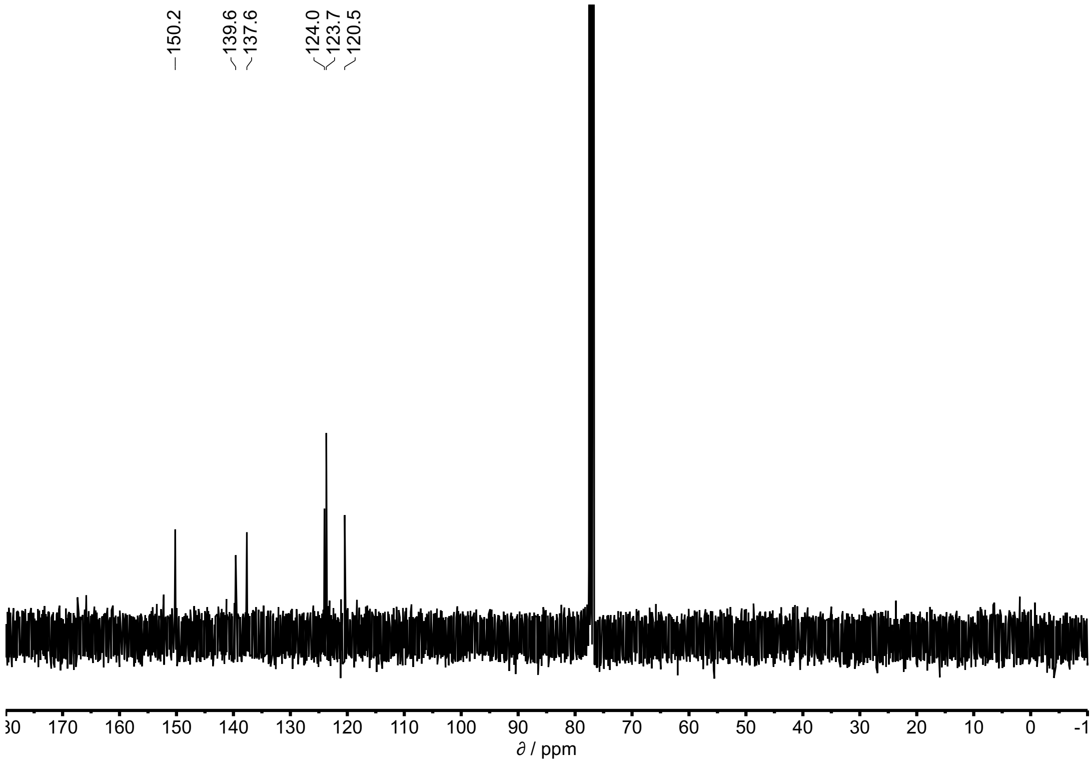

Figure S6. *^{13}C NMR spectrum (101 MHz, $CDCl_3$) of compound* ***7****.*

4-(Pyrimidin-2-yl)pyridine 1-oxide (8)

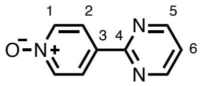

White solid (8 mg, 0.05 mmol, 33 %).

Mpt: 198.2 – 199.4 °C

^{1}H NMR (400 MHz, $CDCl_3$): δ_H 8.83 (d, *J* = 4.8 Hz, 2H, H-5), 8.39 (d, *J* = 7.3 Hz, 2H, H-2), 8.32 (d, *J* = 7.3 Hz, 2H, H-1), 7.30 – 7.26 (m, 1H, H-6)

^{13}C NMR (101 MHz, $CDCl_3$): δ_C 161.1 (C-4), 157.7 (C-5), 139.6 (C-1), 135.9 (C-3), 125.0 (C-2), 120.3 (C-6).

HRMS (ES+): calculated for $C_9H_7N_3O$ 174.0662 [M+H$^+$], found 174.0668 [M+H$^+$].

FT-IR (ATR): ν_{max} 3060, 1739, 1562, 1541, 1490, 1407, 1268, 1171, 814, 797, 607 cm^{-1}.

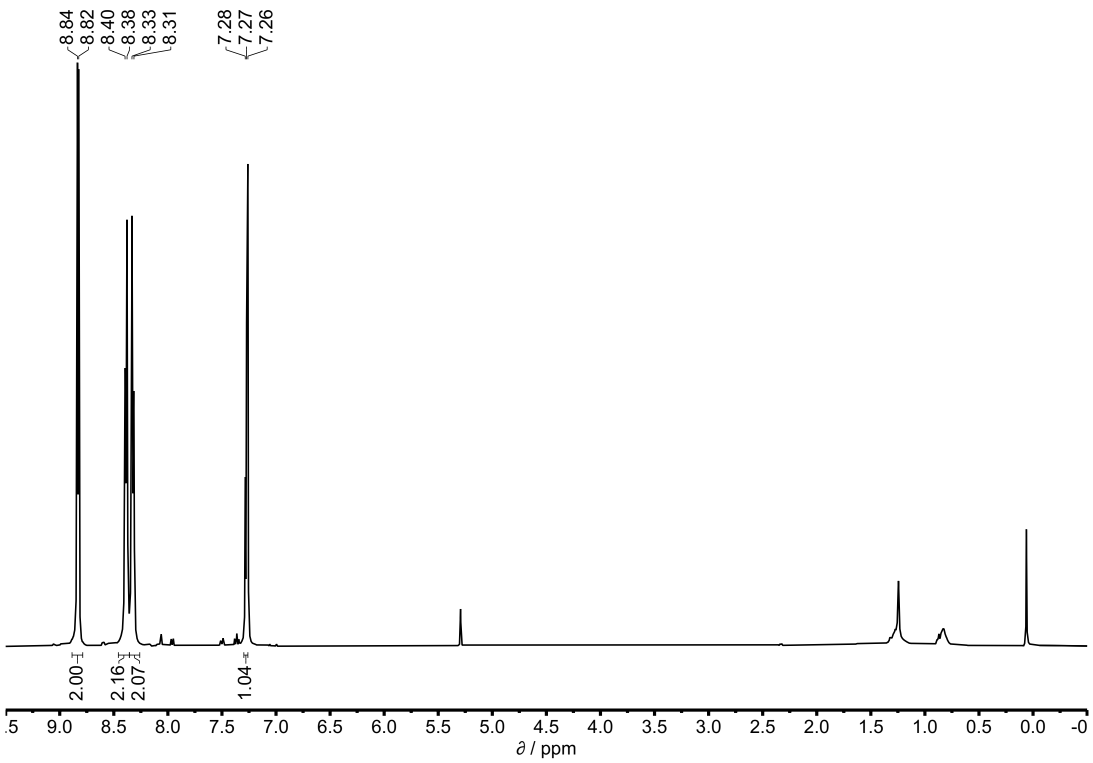

Figure S7. *^{1}H NMR spectrum (400 MHz, $CDCl_3$) of compound **8**.*

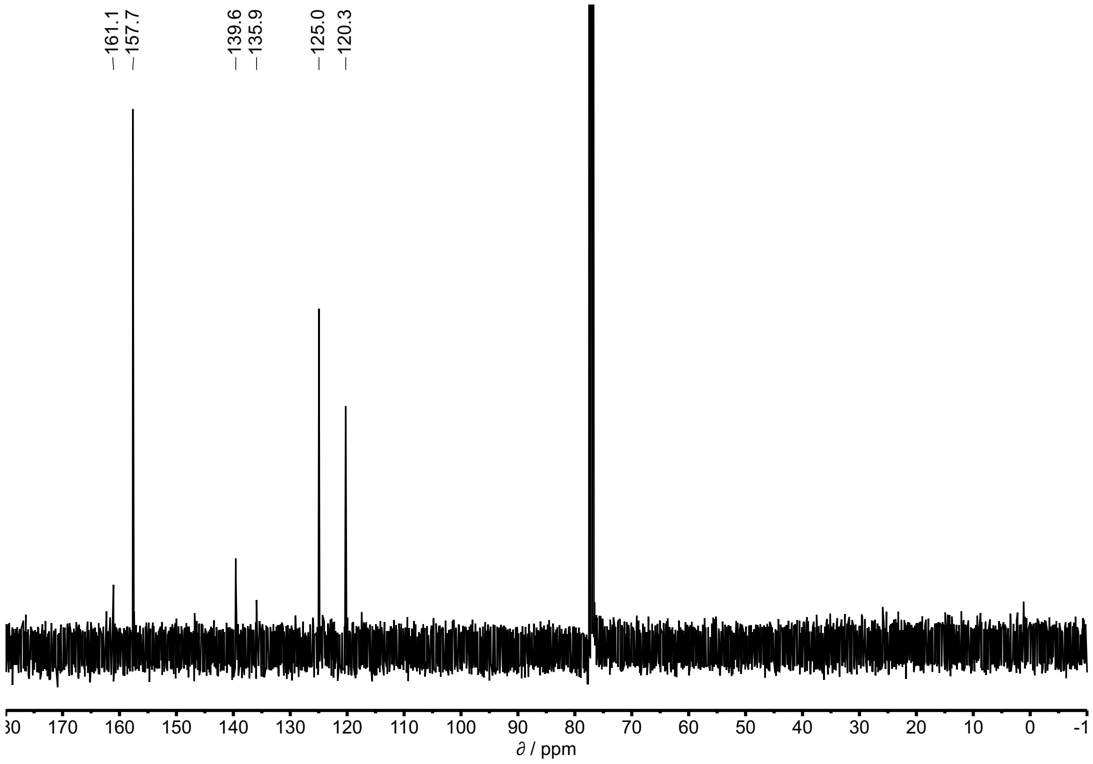

Figure S8. *^{13}C NMR spectrum (101 MHz, $CDCl_3$) of compound **8**.*

4-(Pyrimidin-5-yl)pyridine 1-oxide (9)

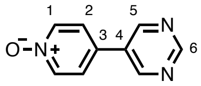

White solid (37 mg, 0.21 mmol, 32 %)

Mpt: 227.9 – 229.1 °C

^{1}H NMR (400 MHz, CDCl$_3$): δ_H 9.28 (s, 1H, H-6), 8.98 (s, 2H, H-5), 8.34 (d, J = 6.7 Hz, 2H, H-1), 7.54 (d, J = 6.8 Hz, 2H, H-2).

^{13}C NMR (101 MHz, CDCl$_3$): δ_C 158.9 (C-6), 154.49 (C-5), 140.3 (C-1), 131.7 (C-3), 130.2 (C-4), 123.9 (C-2).

HRMS (ES+): calculated for $C_9H_7N_3O$ 174.0662 [M+H$^+$], found 174.0654 [M+H$^+$].

FT-IR (ATR): ν_{max} 3046, 1739, 1562, 1495, 1415, 1263, 1189, 1034, 852, 761, 717, 631, 597, 555 cm^{-1}.

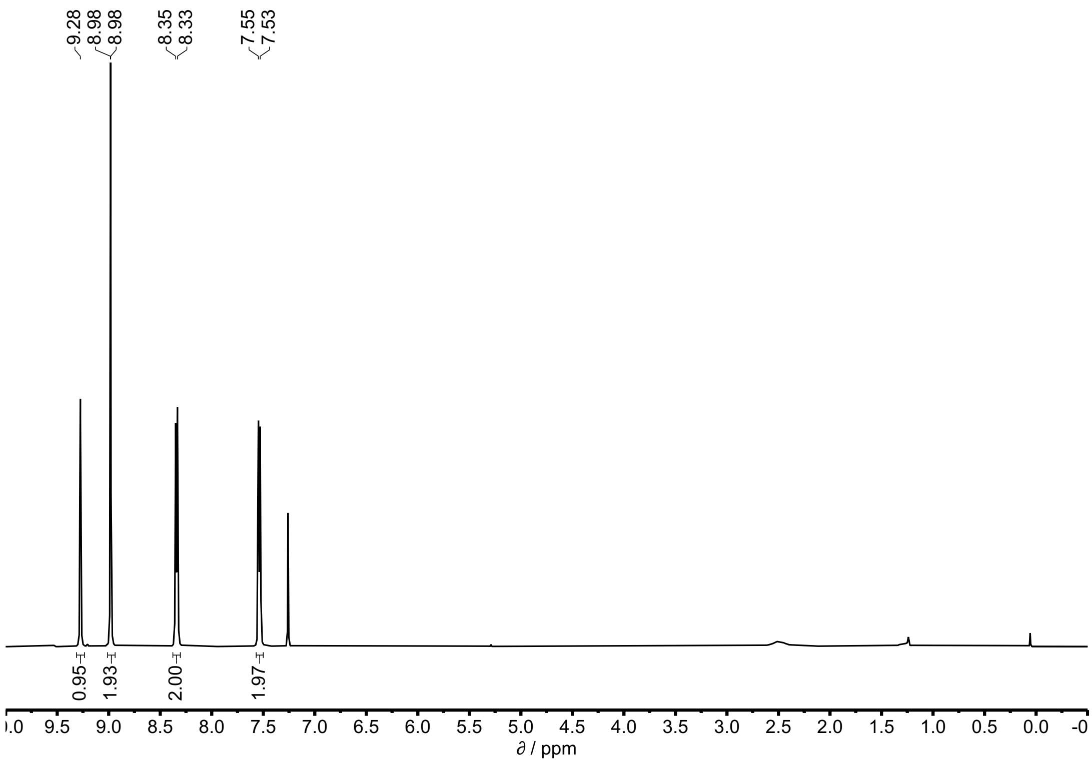

Figure S9. *^{1}H NMR spectrum (400 MHz, CDCl3) of compound* ***9****.*

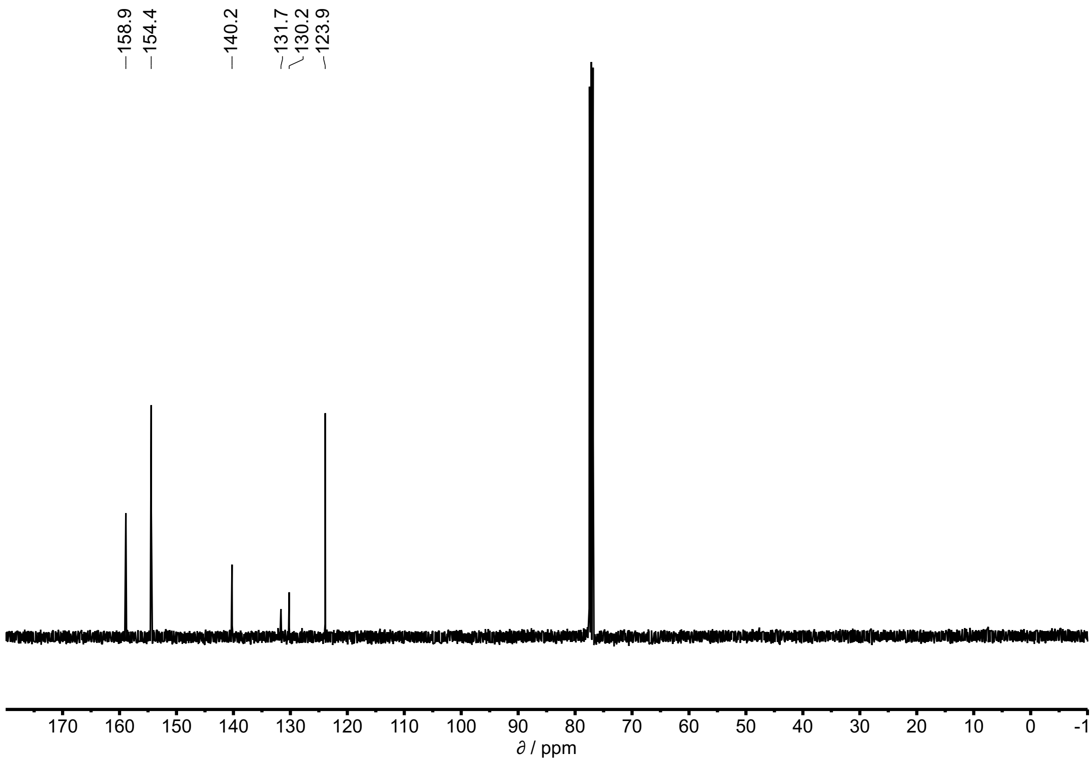

Figure S10. *^{13}C NMR spectrum (101 MHz, CDCl3) of compound* ***9****.*

4-(Furan-2-yl)pyridine 1-oxide (10)

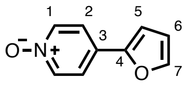

Light brown solid (65 mg, 0.40 mmol, 73 %)

Mpt: 155.9 – 158.9 °C

^{1}H NMR (400 MHz, DMSO-d$_6$): δ_H 8.21 (d, *J* = 7.4 Hz, 2H, H-1), 7.85 (d, *J* = 1.0 Hz, 1H, H-7), 7.67 (d, *J* = 7.4 Hz, 2H, H-2), 7.18 (d, *J* = 4.2 Hz, 1H, H-5), 6.68 – 6.65 (m, 1H, H-6).

^{13}C NMR (101 MHz, DMSO-d$_6$): δ_C 149.8 (C-4), 144.5 (C-7), 139.0 (C-1), 126.3 (C-3), 120.4 (C-2), 112.7 (C-6), 109.0 (C-5).

HRMS (ES+): calculated for $C_9H_7NO_2$ 162.0550 [M+H$^+$], found 162.0553 [M+H$^+$].

FT-IR (ATR): ν_{max} 3103, 3025, 1470, 1445, 1237, 1184, 1019, 8594, 838, 741, 638 cm^{-1}.

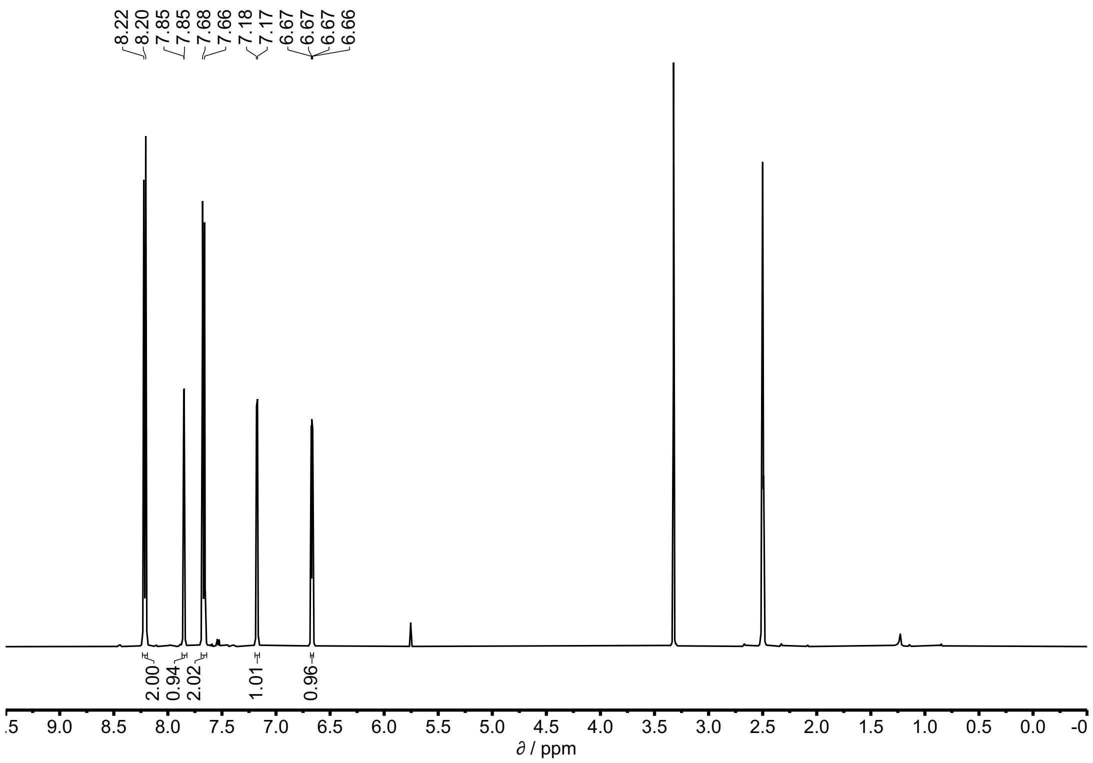

Figure S11. *^{1}H NMR spectrum (400 MHz, DMSO-d6) of compound* ***10****.*

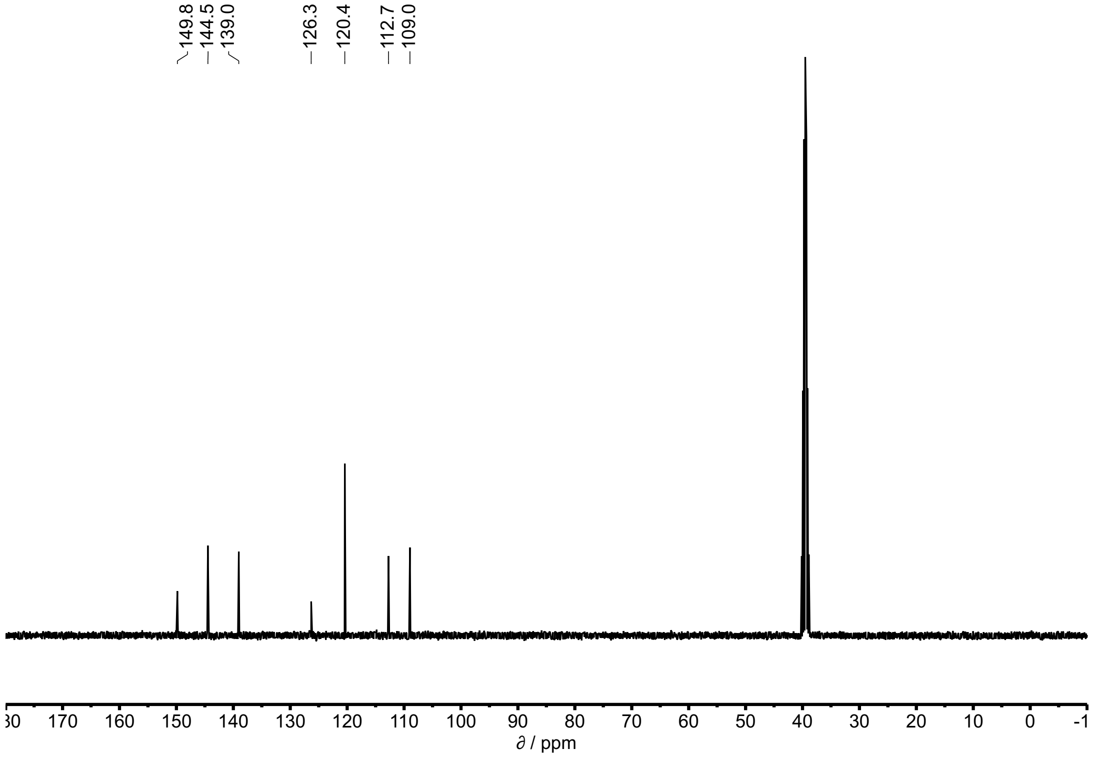

Figure S12. *^{13}C NMR spectrum (101 MHz, DMSO-d6) of compound* ***10****.*

4-(Furan-3-yl)pyridine 1-oxide (11)

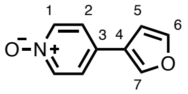

Light pink solid (63 mg, 0.39 mmol, 78 %)

Mpt: 162.9 – 164.9 °C

^{1}H NMR (400 MHz, DMSO-d$_6$): δ_H 8.38 (s, 1H, H-7), 8.21 (d, J = 7.3 Hz, 2H, H-1), 7.80 (t, J = 1.7 Hz, 1H, H-6), 7.66 (d, J = 7.3 Hz, 2H, H-2), 7.06 (dd, J = 1.9, 0.9 Hz, 1H, H-5).

^{13}C NMR (101 MHz, DMSO-d$_6$): δ_C 145.0 (C-6), 141.2 (C-7), 138.8 (C-1), 128.9 (C-3), 122.9 (C-4), 122.7 (C-2), 108.3 (C-5).

HRMS (ES+): calculated for $C_9H_7NO_2$ 162.0550 [M+H$^+$], found 162.0553 [M+H$^+$].

FT-IR (ATR): ν_{max} 31105, 1511, 1477, 1450, 1231, 1163, 1101, 1019, 731, 630, 594 cm^{-1}.

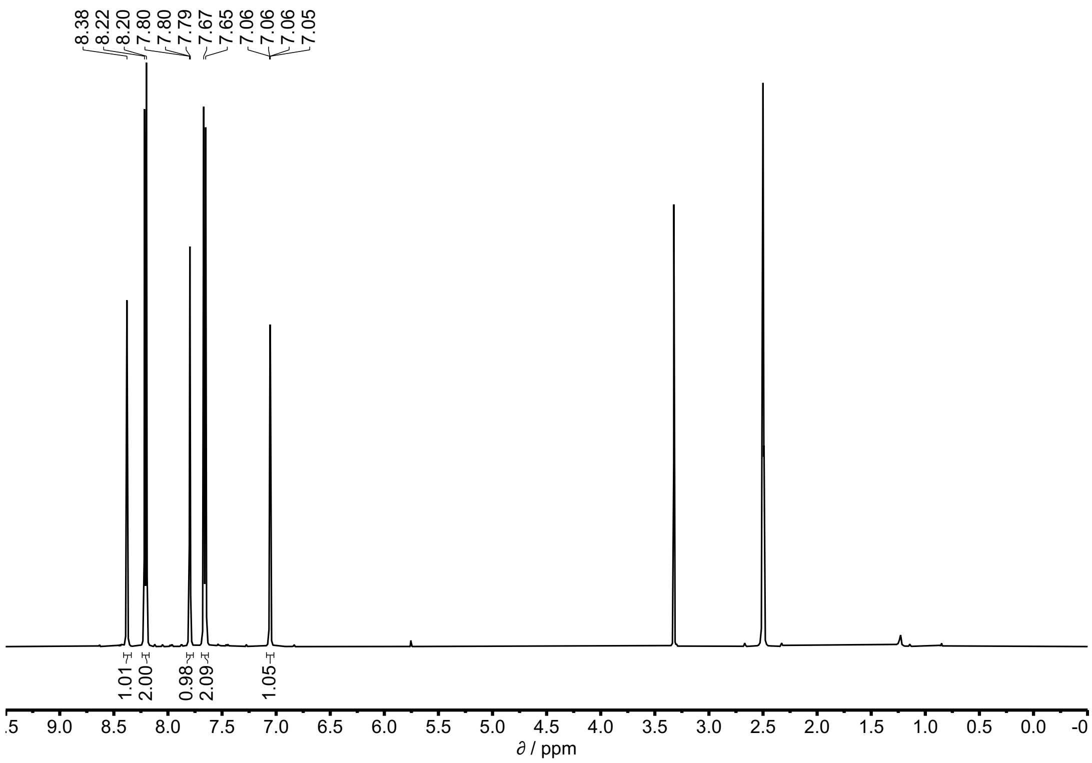

Figure S13. *¹H NMR spectrum (400 MHz, DMSO-d₆) of compound* ***11****.*

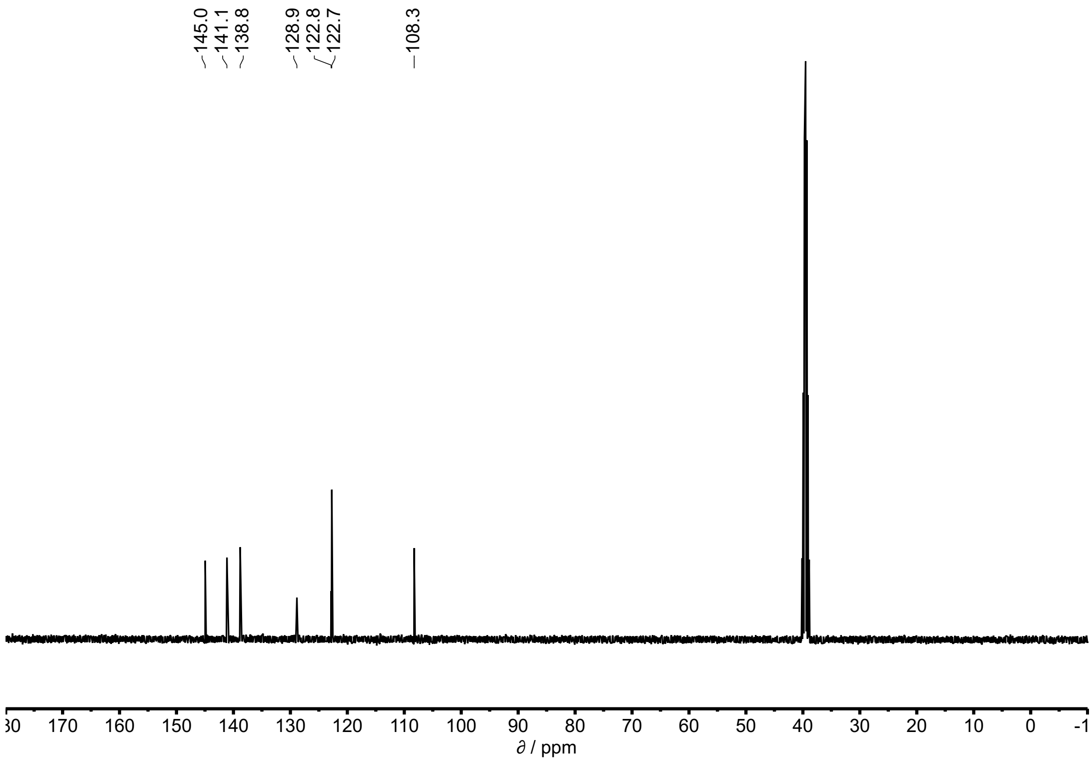

Figure S14. *¹³C NMR spectrum (101 MHz, DMSO-d₆) of compound* ***11****.*

4-(Thiophen-2-yl)pyridine 1-oxide (12)

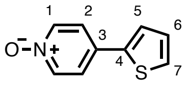

Off white solid (71 mg, 0.40 mmol, 69 %)

Mpt: 181.9 – 183.9 °C

^{1}H NMR (400 MHz, DMSO-d$_6$): δ_H 8.19 (d, *J* = 7.2 Hz, 2H, H-1), 7.75 – 7.63 (m, 4H, H-2, H-5, H-7), 7.19 (dd, *J* = 5.1, 3.7 Hz, 1H, H-6).

^{13}C NMR (101 MHz, DMSO-d$_6$): δ_C 139.3 (C-4), 139.0 (C-1), 130.2 (C-3), 129.0 (C-6), 127.8 (C-7), 125.9 (C-5), 122.3 (C-2).

HRMS (ES+): calculated for C_9H_7NOS 177.0248 [M+H$^+$], found 178.0322 [M+H$^+$].

FT-IR (ATR): ν_{max} 3068, 1526, 1480, 1264, 1248, 1174, 838, 733, 699, 586 cm^{-1}.

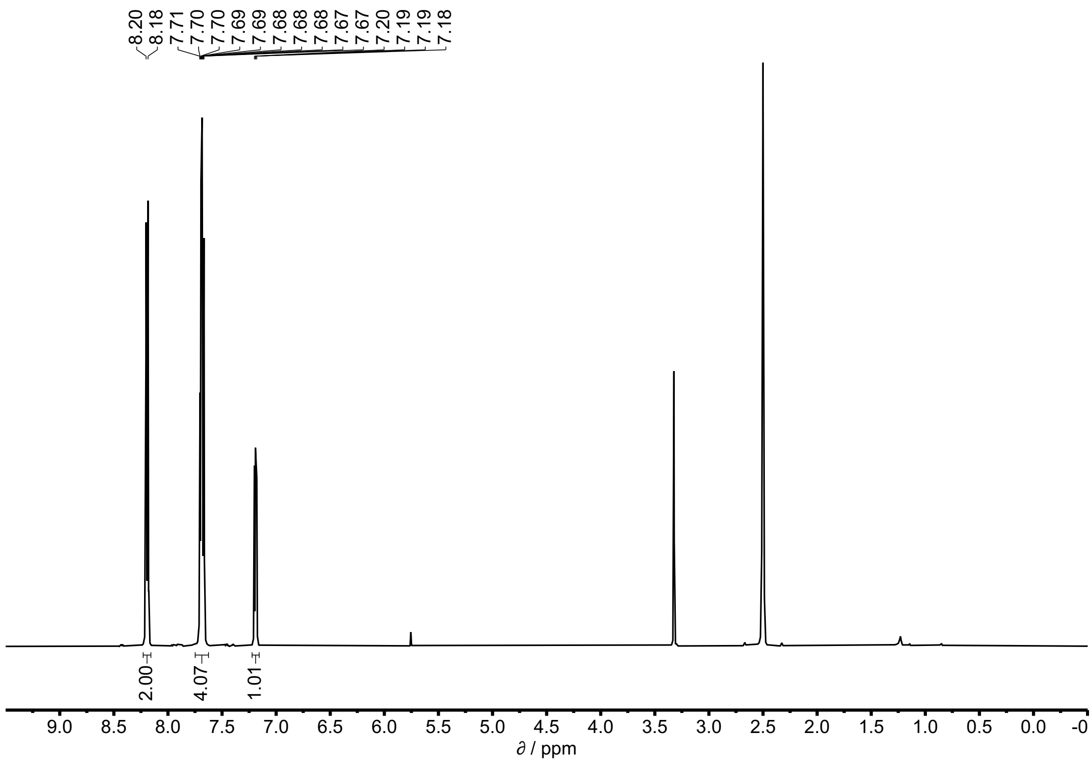

Figure S15. *^{1}H NMR spectrum (400 MHz, DMSO-d_6) of compound* ***12****.*

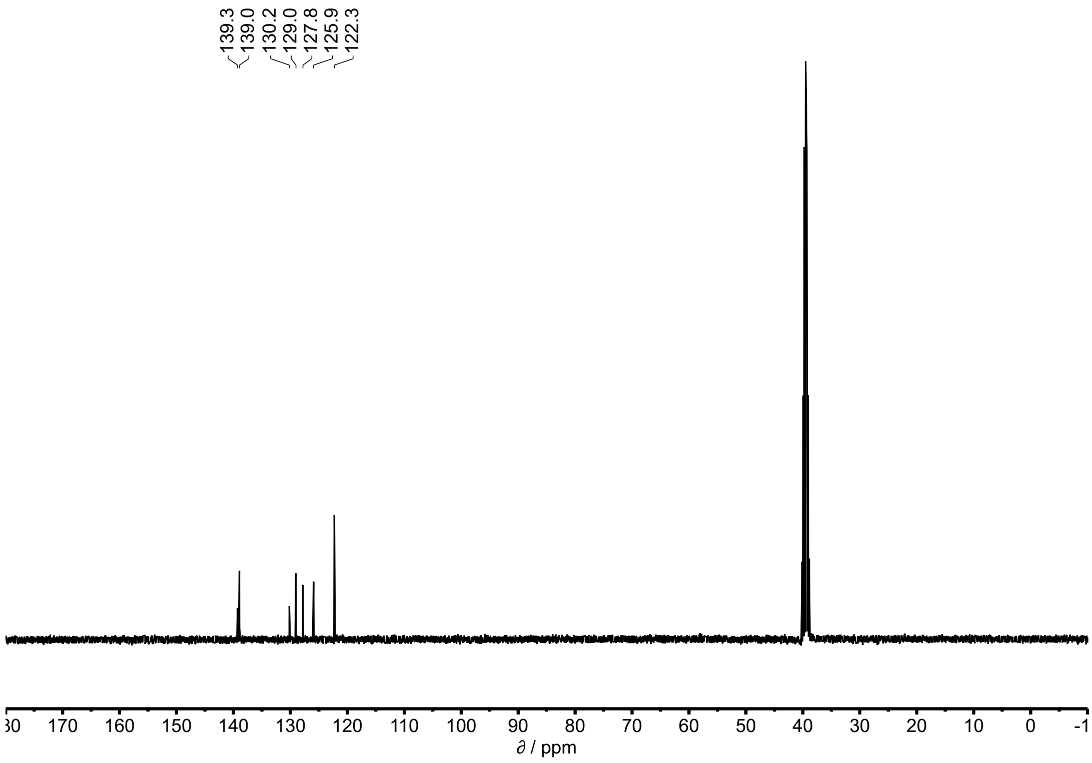

Figure S16. *^{13}C NMR spectrum (101 MHz, DMSO-d_6) of compound* ***12****.*

4-(Thiophen-3-yl)pyridine 1-oxide (13)

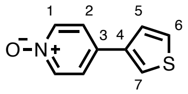

Brown solid (80 mg, 0.45 mmol, 72 %)

Mpt: 241.6 -244.6 °C

^{1}H NMR (400 MHz, DMSO-d$_6$): δ_H 8.22 (d, *J* = 7.3 Hz, 2H, H-1), 8.13 (dd, *J* = 2.9, 1.4 Hz, 1H, H-7), 7.78 (d, *J* = 7.2 Hz, 2H, H-2), 7.70 (dd, *J* = 5.0, 2.9 Hz, 1H, H-5), 7.65 (dd, *J* = 5.1, 1.4 Hz, 1H, H-6).

^{13}C NMR (101 MHz, DMSO-d$_6$): δ_C 138.8 (C-1), 137.5 (C-4), 131.5 (C-3), 127.9 (C-5), 125.8 (C-6), 123.3 (C-7), 123.1 (C-2).

HRMS (ES+): calculated for C_9H_7NOS 177.0248 [M+H$^+$], found 178.0318 [M+H$^+$].

FT-IR (ATR): ν_{max} 3054, 1742, 1484, 1450, 1249, 1229, 1197, 1177, 1032, 842, 810, 780 cm^{-1}.

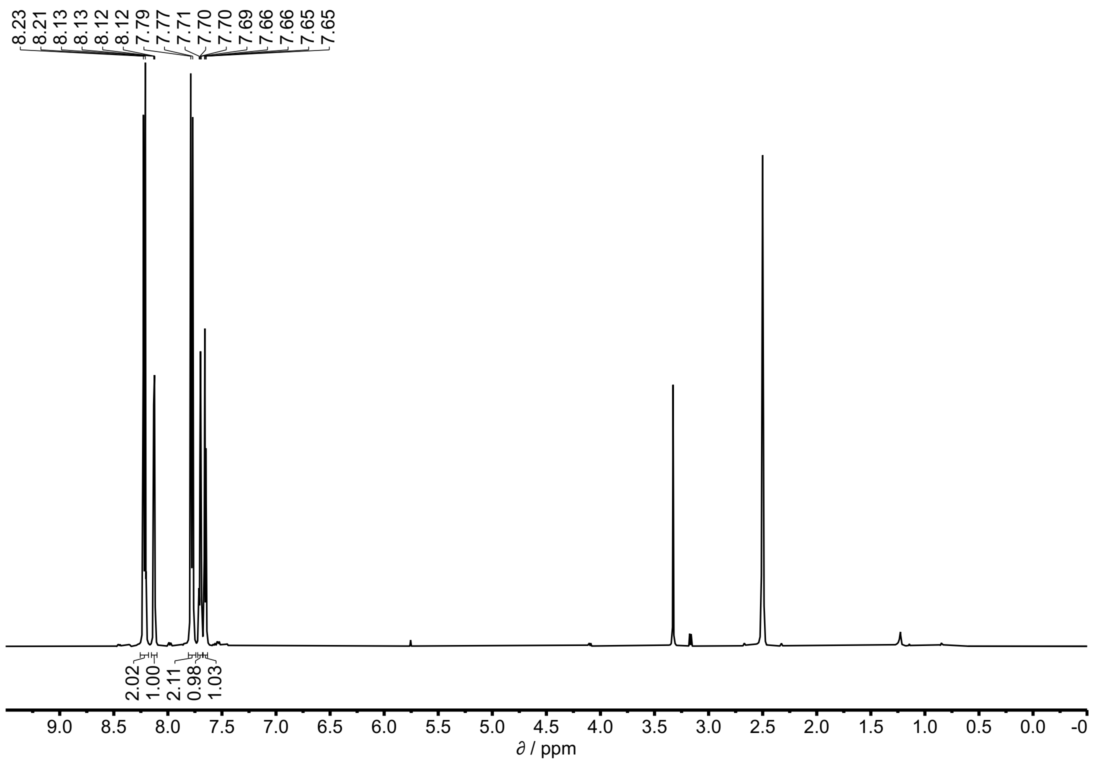

Figure S17. *^{1}H NMR spectrum (400 MHz, DMSO-d_6) of compound* ***13****.*

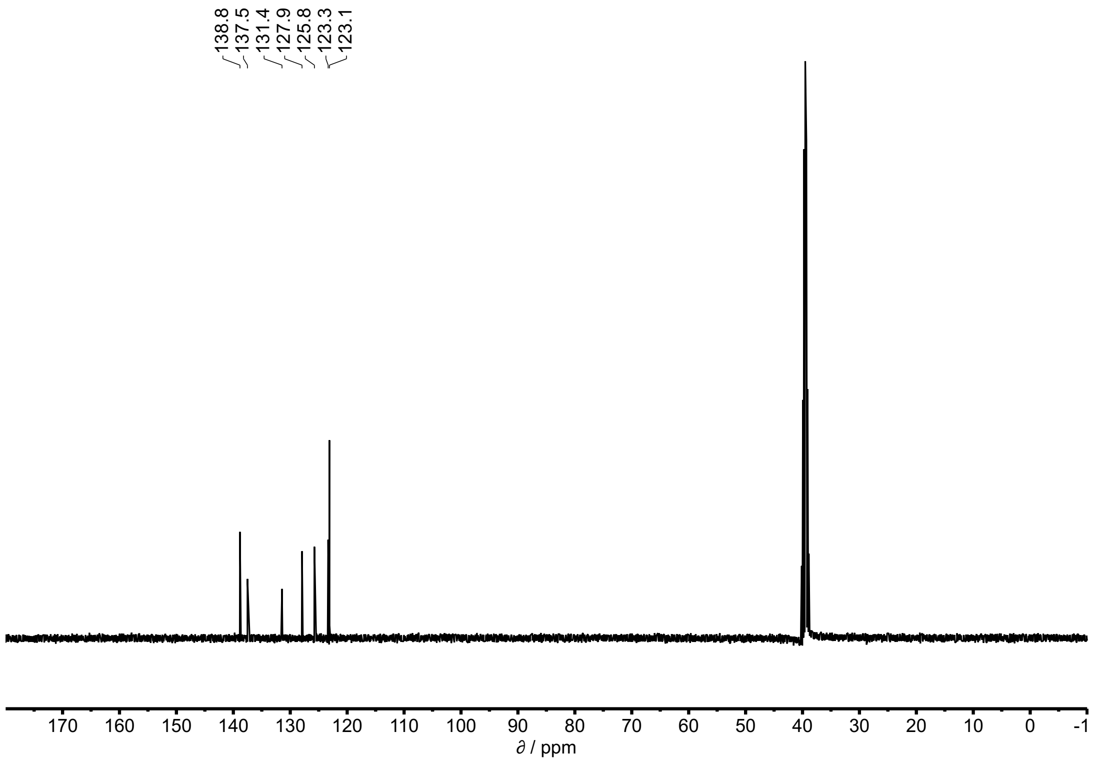

Figure S18. *^{13}C NMR spectrum (101 MHz, DMSO-d_6) of compound* ***13****.*

4-(Thiazol-5-yl)pyridine 1-oxide (14)

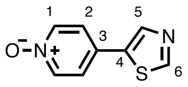

White solid (29 mg, 0.16 mmol, 35 %)

Mpt: 171.4 – 172.4 °C

^{1}H NMR (400 MHz, $CDCl_3$): δ_H 8.86 (s, 1H, H-6), 8.23 (d, *J* = 7.2 Hz, 2H, H-1), 8.17 (s, 1H, H-5), 7.47 (d, *J* = 7.2 Hz, 2H, H-2).

^{13}C NMR (101 MHz, $CDCl_3$): δ_C 154.0 (C-6), 141.1 (C-5), 139.8 (C-1), 135.18 (C-4), 129.2 (C-3), 123.6 (C-2).

HRMS (ES+): calculated for $C_8H_6N_2OS$ 179.0274 [M+H$^+$], found 179.0273 [M+H$^+$].

FT-IR (ATR): ν_{max} 3073, 1523, 1473, 1446, 1247, 1231, 1179, 842, 592 cm^{-1}.

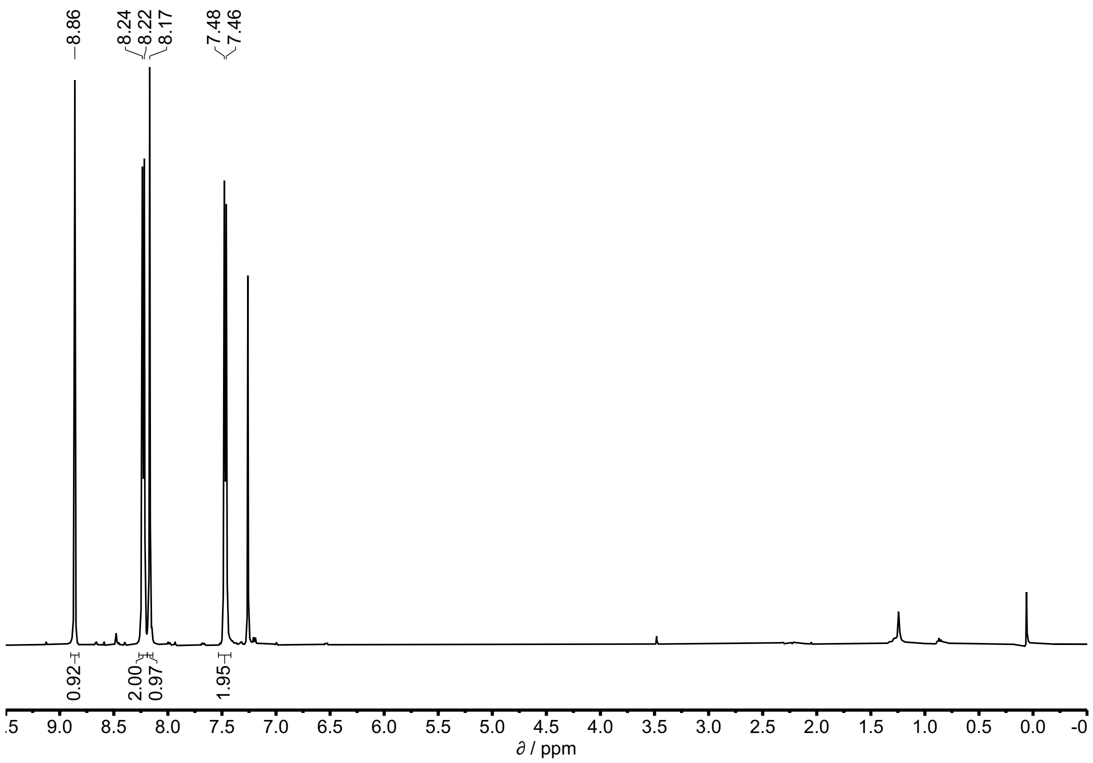

Figure S19. *¹H NMR spectrum (400 MHz, CDCl₃) of compound* ***14****.*

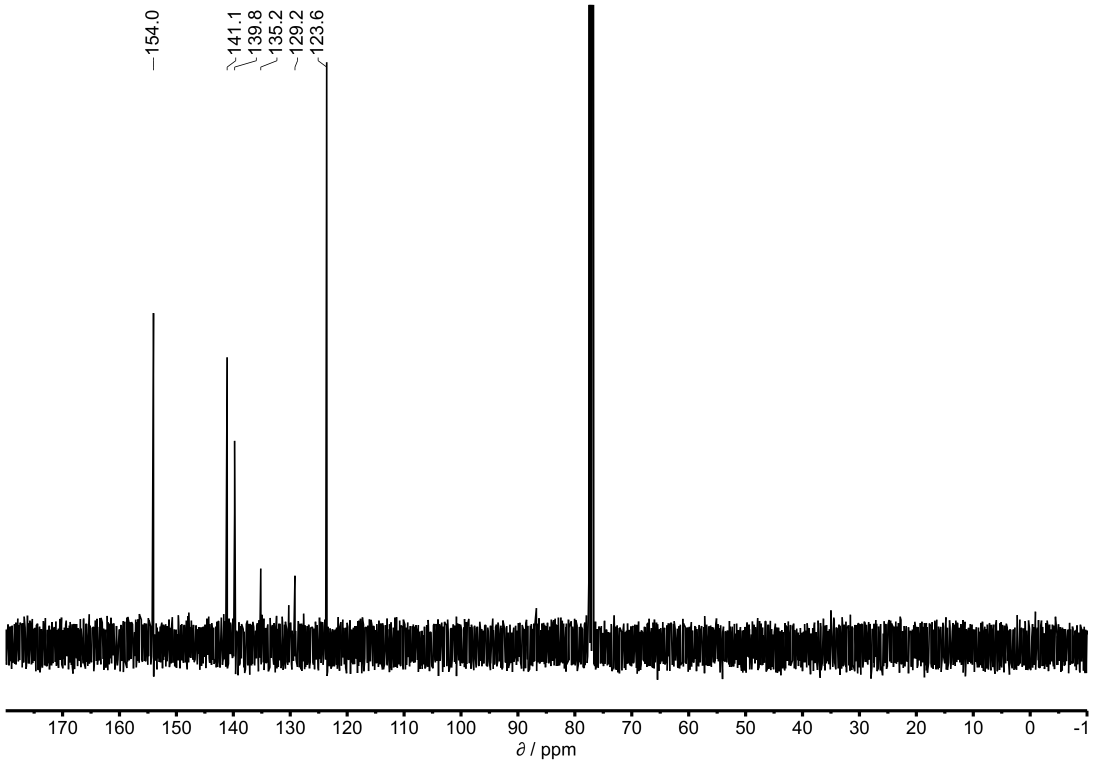

Figure S20. *¹³C NMR spectrum (101 MHz, CDCl₃) of compound* ***14****.*

4-(Thiazol-4-yl)pyridine 1-oxide (15)

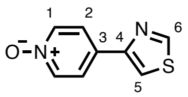

Off white solid (23 mg, 0.13 mmol, 52 %)

Mpt: 158.9 – 159.6 °C

^{1}H NMR (400 MHz, CDCl$_3$): δ_H 8.91 (d, J = 1.9 Hz, 1H, H-6), 8.27 (d, J = 7.2 Hz, 2H, H-1), 7.85 (d, J = 7.2 Hz, 2H, H-2), 7.69 (d, J = 1.9 Hz, 1H, H-5).

^{13}C NMR (101 MHz, CDCl$_3$): δ_C 154.1 (C-6), 152.1 (C-4), 139.6 (C-1), 123.3 (C-2), 115.8 (C-5).

HRMS (ES+): calculated for $C_8H_6N_2OS$ 179.0274 [M+H$^+$], found 179.0270 [M+H$^+$].

FT-IR (ATR): ν_{max} 3126, 1739, 1516, 1461, 1438, 1254, 1170, 719, 670, 651, 595 cm^{-1}.

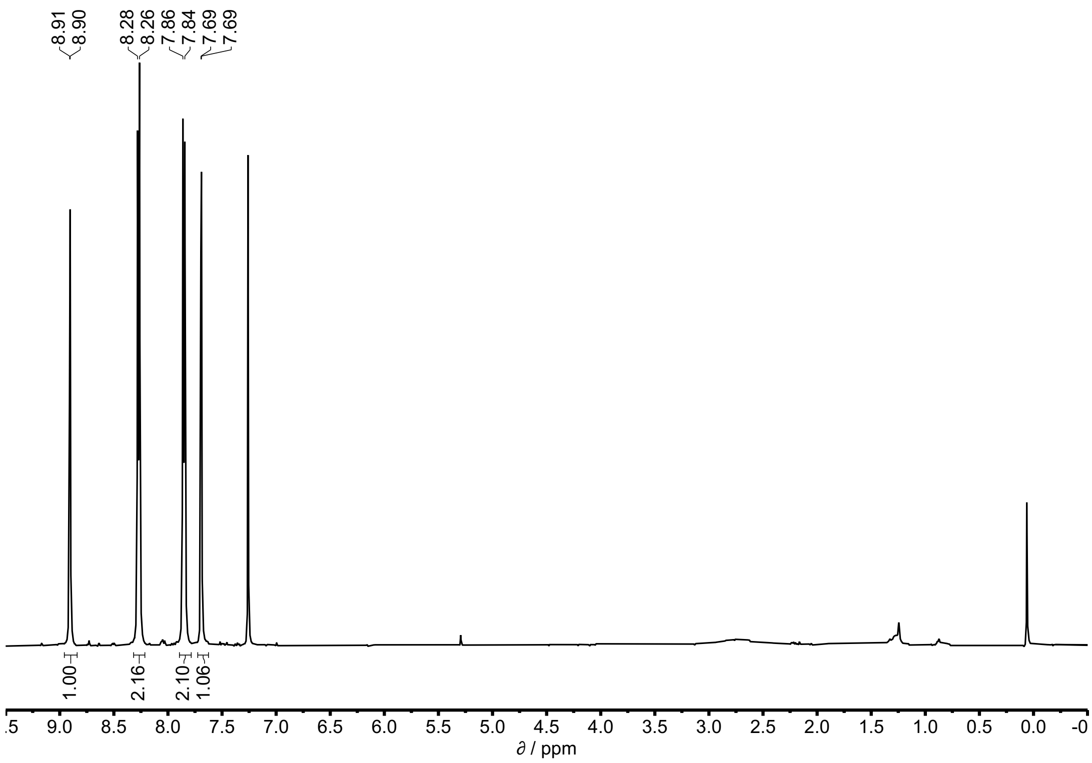

***Figure S21.** ^{1}H NMR spectrum (400 MHz, $CDCl_3$) of compound **15**.*

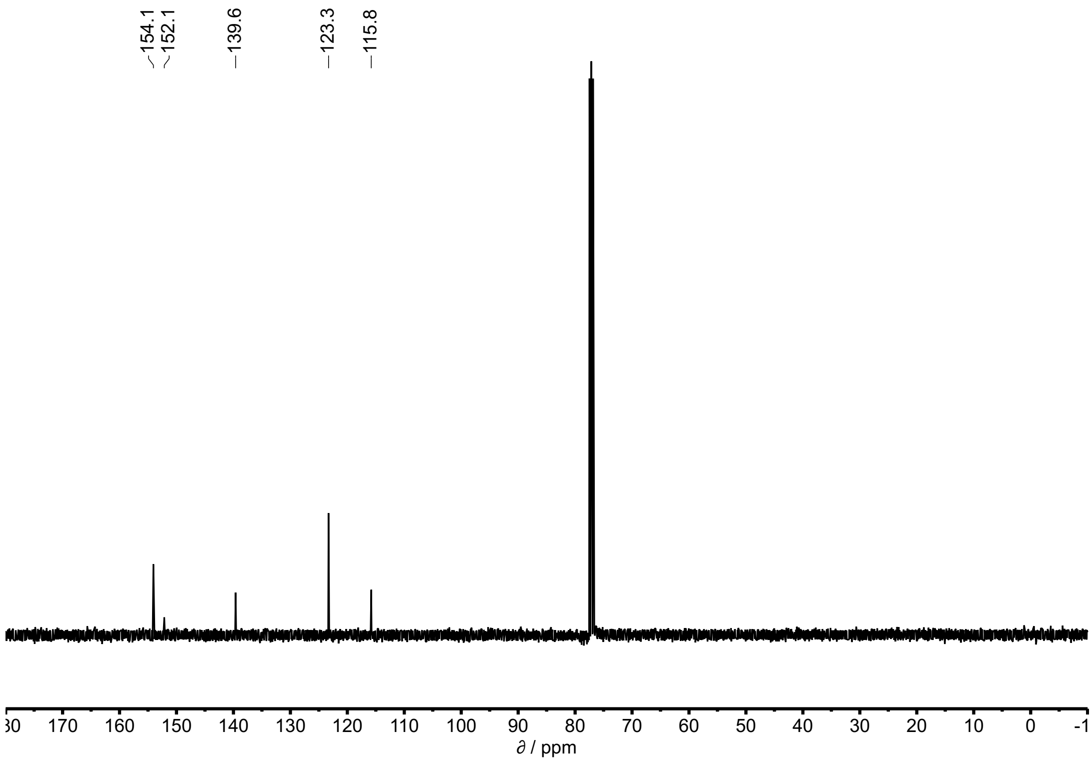

***Figure S22.** ^{13}C NMR spectrum (101 MHz, $CDCl_3$) of compound **15**.*

2,4'-Bipyridine (16)

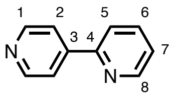

Off-white solid (34 mg, 0.22 mmol, 34 %)

^{1}H NMR (400 MHz, $CDCl_3$): δ_H 8.76 – 8.73 (m, 3H, H-1, H-8), 7.94 (d, *J* = 6.3 Hz, 2H, H-2), 7.85 – 7.80 (m, 2H, H-5, H-6), 7.38 – 7.33 (m, 1H, H-7).

^{13}C NMR (101 MHz, $CDCl_3$): δ_C 154.4 (C-4), 150.2 (C-8), 149.8 (C-1), 147.0 (C-3), 137.1 (C-6), 124.0 (C-7), 121.3 (C-2), 121.0 (C-5).

HRMS (ES+): calculated for $C_{10}H_8N_2$ 157.0760 [M+H$^+$], found 157.0760 [M+H$^+$].

FT-IR (ATR): ν_{max} 3027, 1739, 1584, 1549, 1499, 1462, 1431, 1219, 994, 769, 729, 613 cm^{-1}.

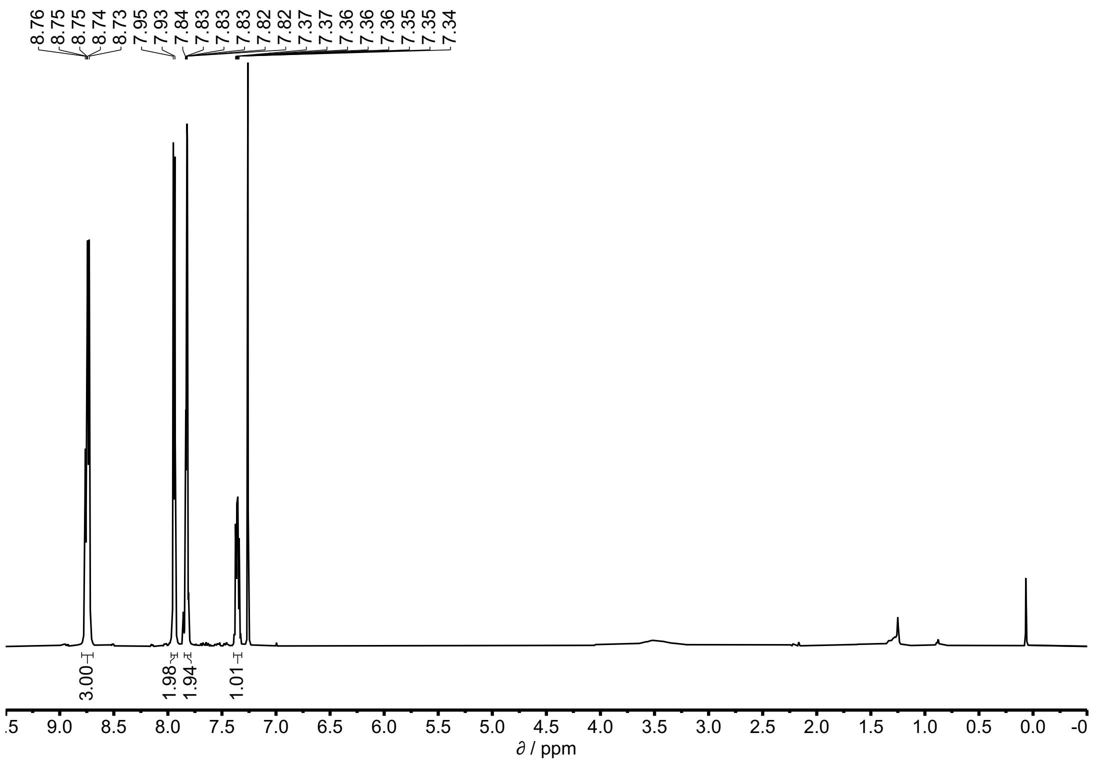

Figure S23. *¹H NMR spectrum (400 MHz, CDCl₃) of compound* ***16****.*

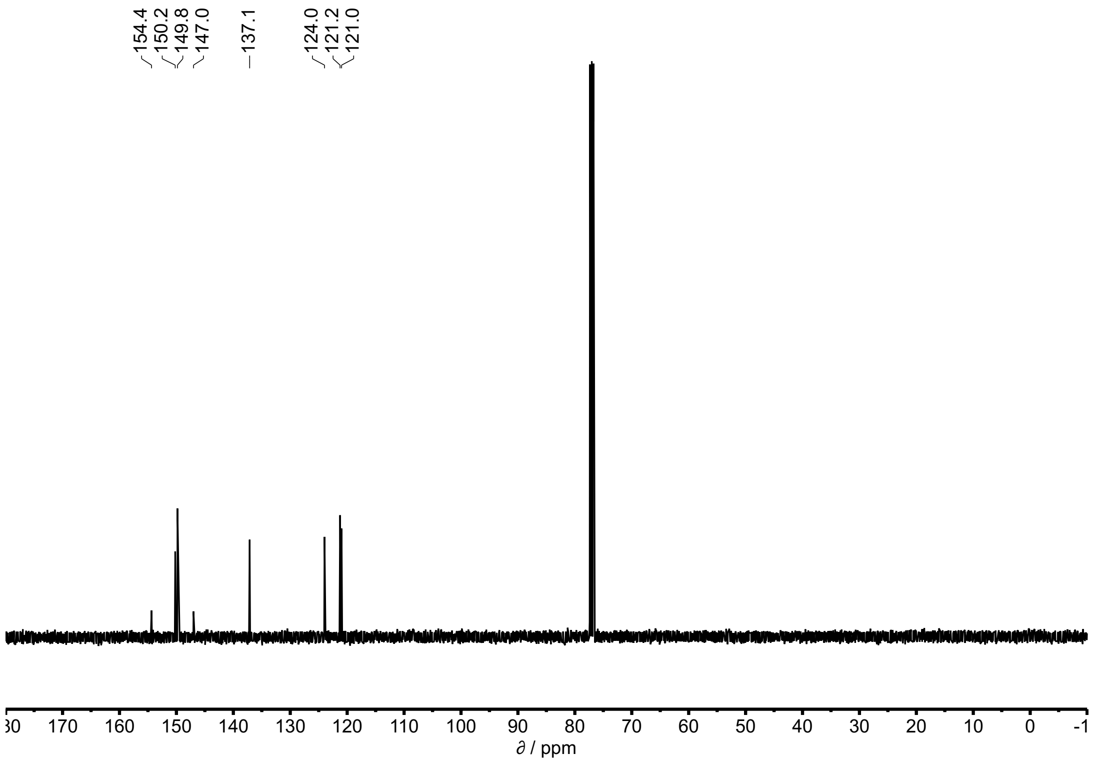

Figure S24. *¹³C NMR spectrum (101 MHz, CDCl₃) of compound* ***16****.*

2-(Pyridin-4-yl)pyrimidine (17)

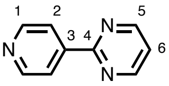

White solid (25 mg, 0.16 mmol, 17 %)

Mpt: 81. 6 – 83.6 °C

^{1}H NMR (400 MHz, CDCl$_3$): δ_H 8.89 (d, J = 4.8 Hz, 2H, H-5), 8.79 (d, J = 6.2 Hz, 2H, H-1), 8.37 (d, J = 6.3 Hz, 2H, H-2), 7.33 (t, J = 4.9 Hz, 1H, H-6).

^{13}C NMR (101 MHz, CDCl$_3$): δ_C 157.7 (C-5), 162.6 (C-4), 149.7 (C-1), 146.0 (C-3) 122.4 (C-2), 120.9 (C-6).

HRMS (ES+): calculated for $C_9H_7N_3$ 158.0713 [M+H$^+$], found 158.0713 [M+H$^+$].

FT-IR (ATR): v_{max} 2925, 1737, 1598, 1564, 1412, 1031, 804, 785, 758 cm^{-1}.

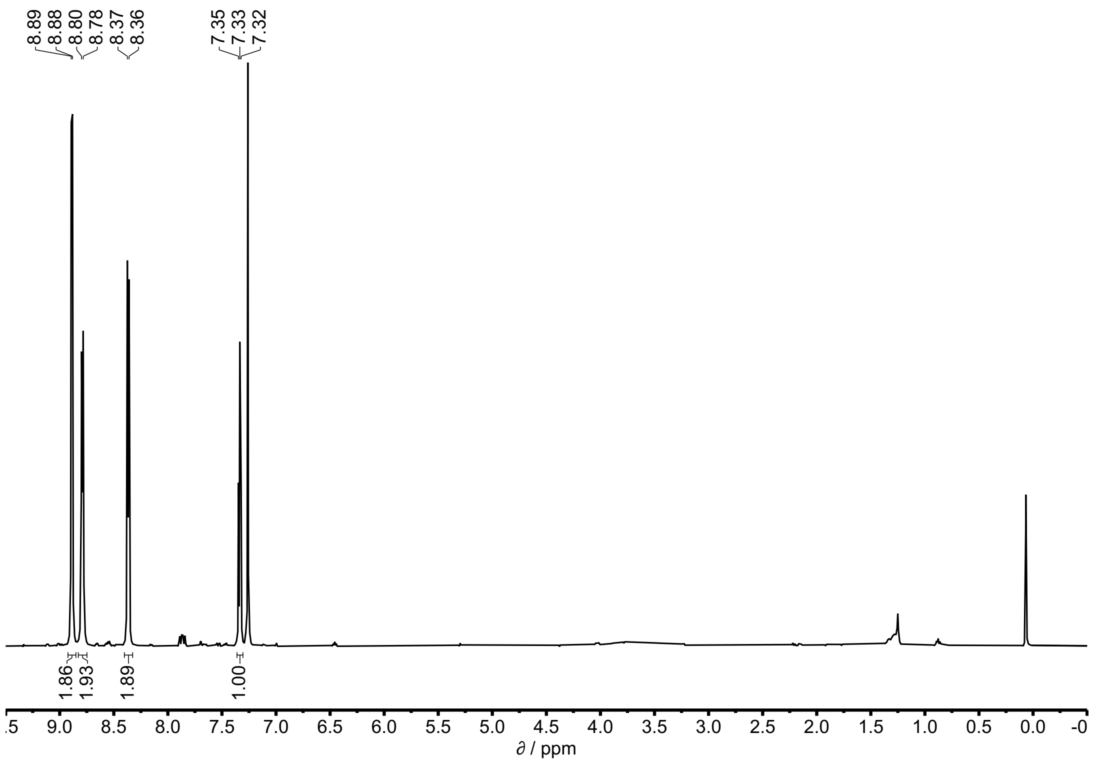

Figure S25. *^{1}H NMR spectrum (400 MHz, $CDCl_3$) of compound* ***17****.*

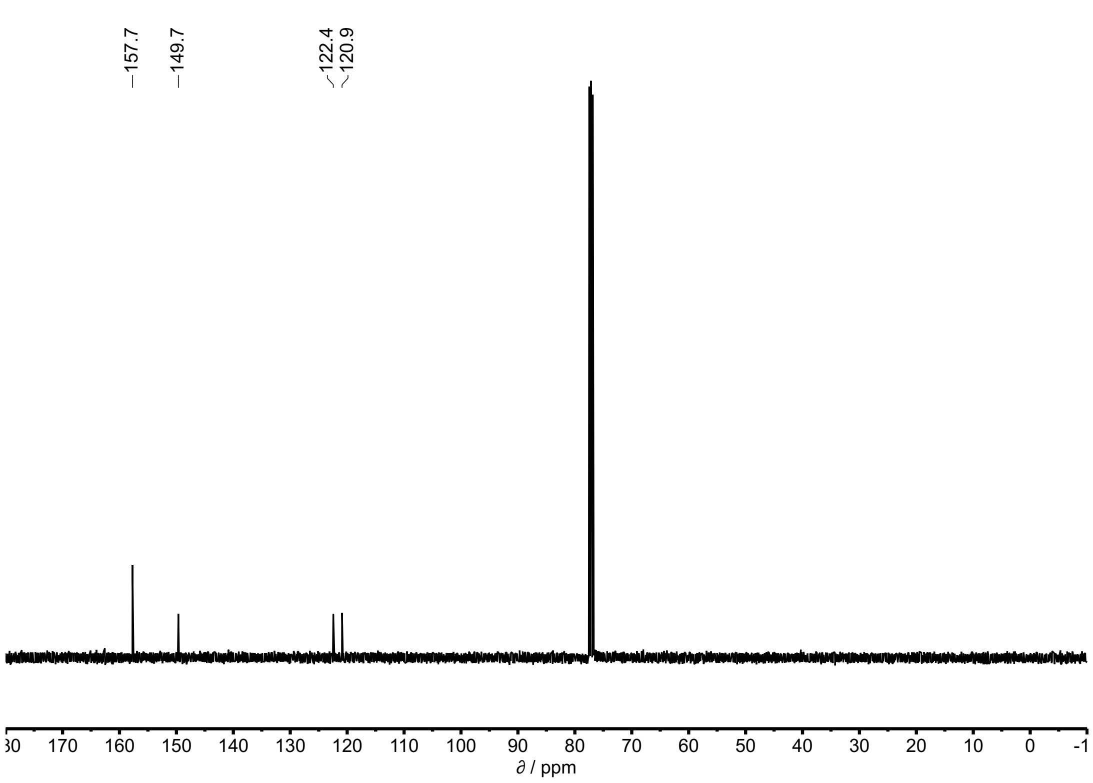

Figure S26. *^{13}C NMR spectrum (101 MHz, $CDCl_3$) of compound* ***17****.*

4-(Pyridin-4-yl)thiazole (18)

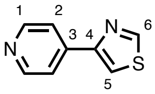

White solid (106 mg, 0.65 mmol, 59 %)

Mpt: 125.6 – 126.6 °C

^{1}H NMR (400 MHz, $CDCl_3$): δ_H 8.93 (d, *J* = 1.9 Hz, 1H, H-6), 8.69 (d, *J* = 6.2 Hz, 2H, H-1), 7.86 (d, *J* = 6.2 Hz, 2H, H-2), 7.82 (d, *J* = 1.9 Hz, 1H, H-5).

^{13}C NMR (101 MHz, $CDCl_3$): δ_C 153.8 (C-6), 153.7 (C-4), 149.8 (C-1), 141.9 (C-3), 121.0 (C-2), 116.8 (C-5).

HRMS (ES+): calculated for $C_8H_6N_2S$ 163.0324 [M+H$^+$], found 163.0323 [M+H$^+$].

FT-IR (ATR): ν_{max} 3033, 1598, 1471, 1422, 1289, 1219, 908, 885, 827, 754, 734, 680 cm^{-1}.

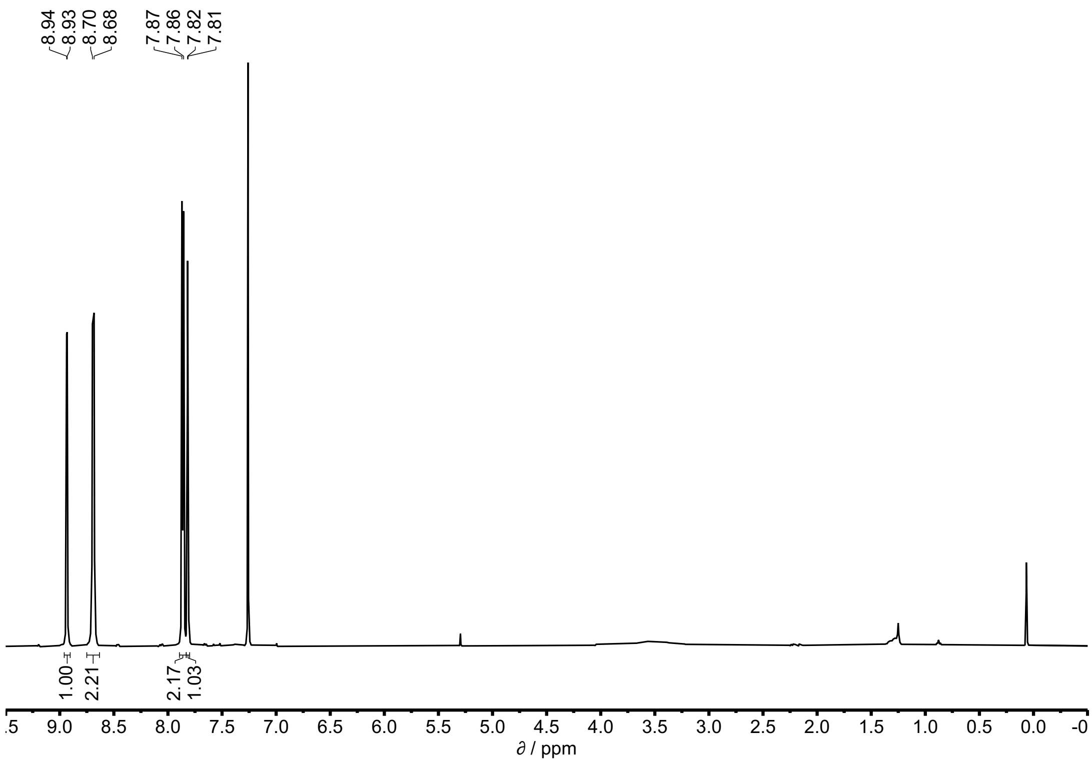

Figure S27. *^{1}H NMR spectrum (400 MHz, $CDCl_3$) of compound* ***18***.

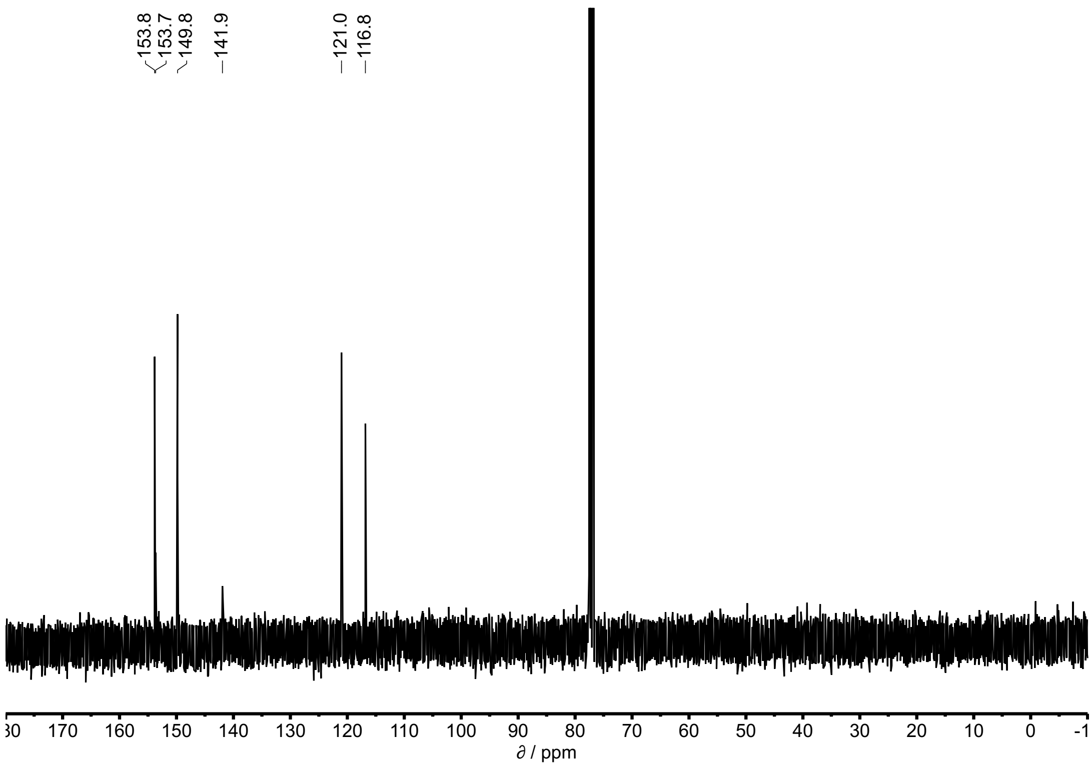

Figure S28. *^{13}C NMR spectrum (101 MHz, $CDCl_3$) of compound* ***18***.

3. Isothermal titration calorimetry (ITC) experiments

In a typical ITC experiment, the host (**1**-**4**) was dissolved in HPLC grade water or chloroform with a concentration 30-40 times the expected dissociation constant, and the solution was loaded into the sample cell of the microcalorimeter. A 7-10 times more concentrated solution of guest (**5**-**15** and **PNO**) was loaded into the injection syringe. The number of injections was 35, and the volume of the injections was 8 µL. The thermogram peaks were integrated and thermodynamic parameters were calculated using the MicroCal PEAQ-ITC Analysis Software which uses the least-squares minimisation to obtain globally minimised parameters. In all cases the data fitted well to a simple 1:1 binding model. Typically, the first injection is ignored in fitting ITC data, but for titrations with compound **4** in chloroform, the first few injections were not used, because there was an increase in the heat released, suggesting that the system was not completely equilibrated. For these experiments, the data points that were not used are indicated on a separate plot as open circles.

3.1. Octapyridinium-super-aryl-extended calix[4]pyrrole 1

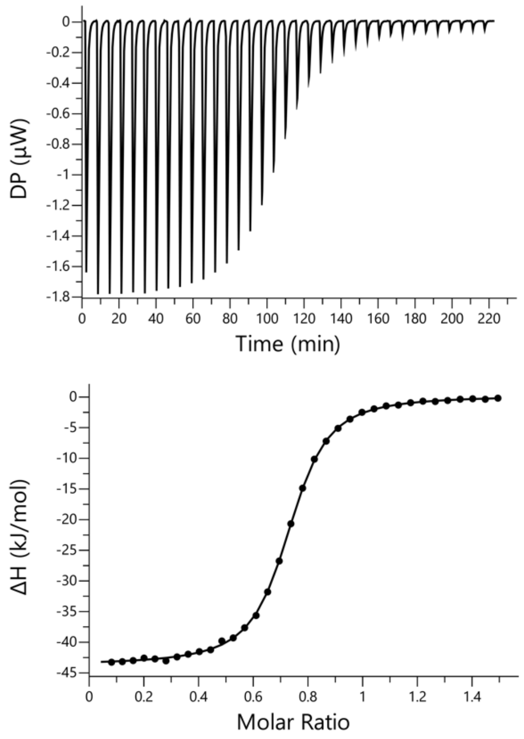

Figure S29. *ITC data for titration of **PNO** (0.28 mM) into **1** (0.04 mM) in water at 298 K. The raw data for each injection is shown (differential power, DP), along with the least-squares-fit of the enthalpy change per mole of guest (ΔH) to a 1:1 binding isotherm.*

3.2. Tetrapyridinium-aryl-extended calix[4]pyrrole 3

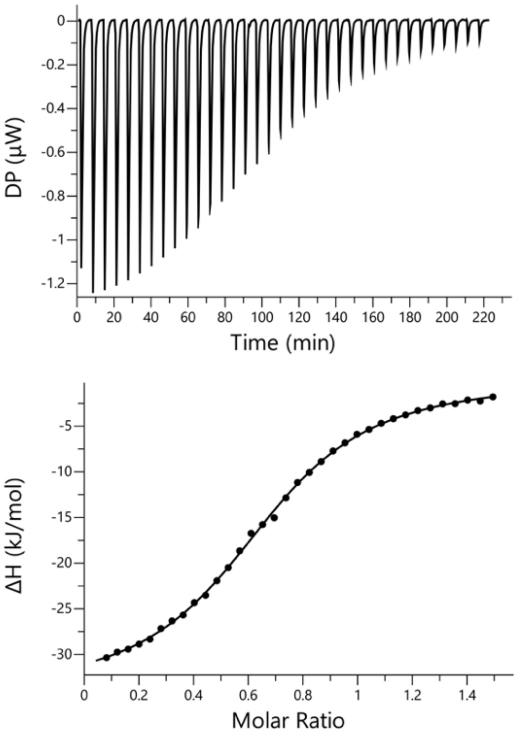

Figure S30. *ITC data for titration of* ***PNO*** *(0.28 mM) into* ***3*** *(0.04 mM) in water at 298 K. The raw data for each injection is shown (differential power, DP), along with the least-squares-fit of the enthalpy change per mole of guest (ΔH) to a 1:1 binding isotherm.*

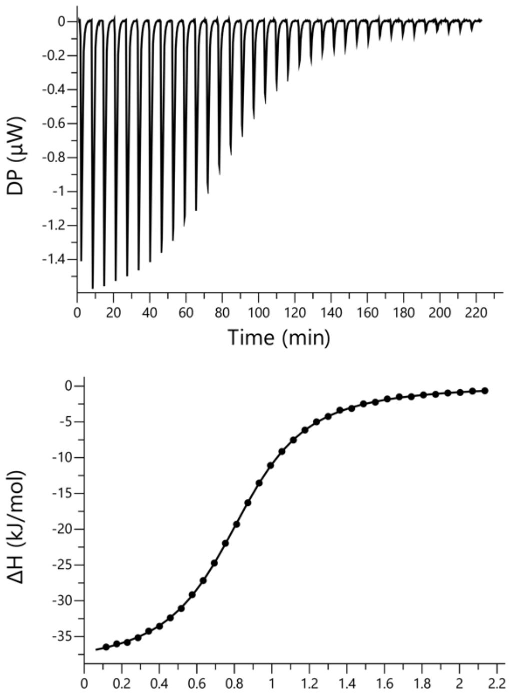

Figure S31. *ITC data for titration of* ***5*** *(0.30 mM) into* ***3*** *(0.03 mM) in water at 298 K. The raw data for each injection is shown (differential power, DP), along with the least-squares-fit of the enthalpy change per mole of guest (ΔH) to a 1:1 binding isotherm.*

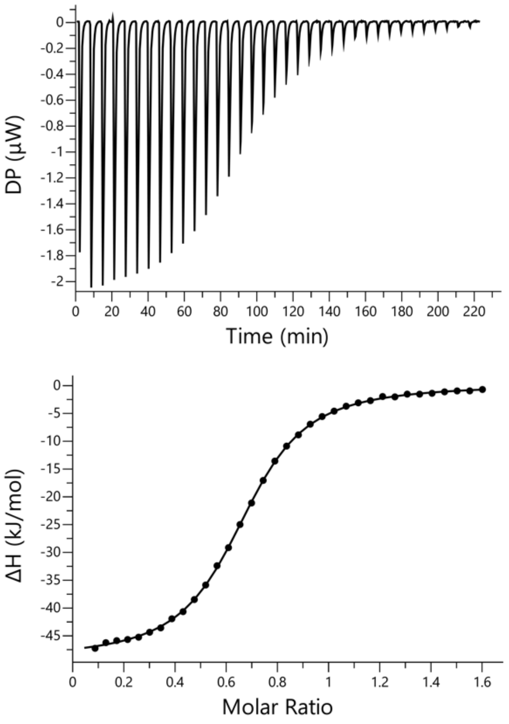

Figure S32. *ITC data for titration of **6** (0.30 mM) into **3** (0.04 mM) in water at 298 K. The raw data for each injection is shown (differential power, DP), along with the least-squares-fit of the enthalpy change per mole of guest (ΔH) to a 1:1 binding isotherm.*

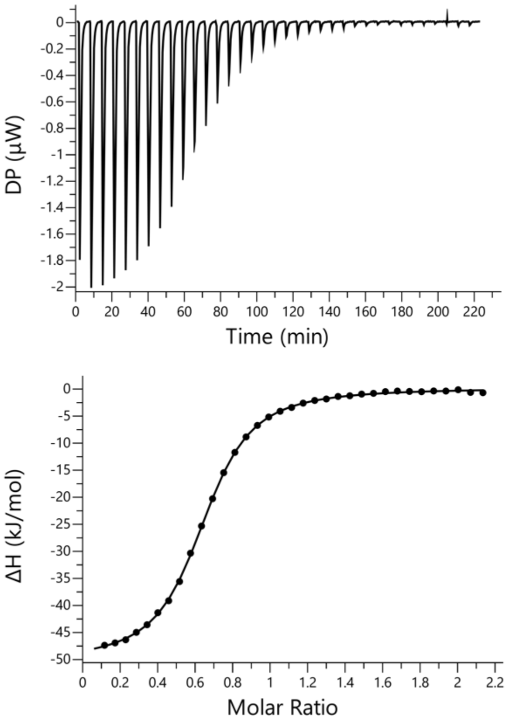

Figure S33. *ITC data for titration of **7** (0.30 mM) into **3** (0.03 mM) in water at 298 K. The raw data for each injection is shown (differential power, DP), along with the least-squares-fit of the enthalpy change per mole of guest (ΔH) to a 1:1 binding isotherm.*

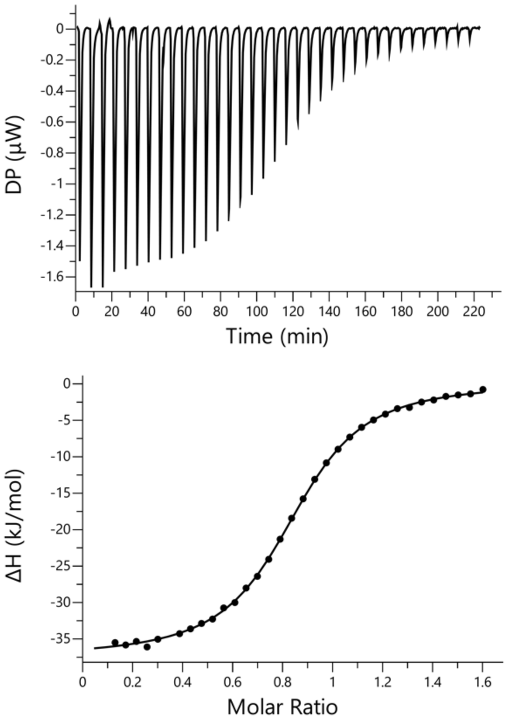

Figure S34. *ITC data for titration of* ***8*** *(0.30 mM) into* ***3*** *(0.04 mM) in water at 298 K. The raw data for each injection is shown (differential power, DP), along with the least-squares-fit of the enthalpy change per mole of guest (ΔH) to a 1:1 binding isotherm.*

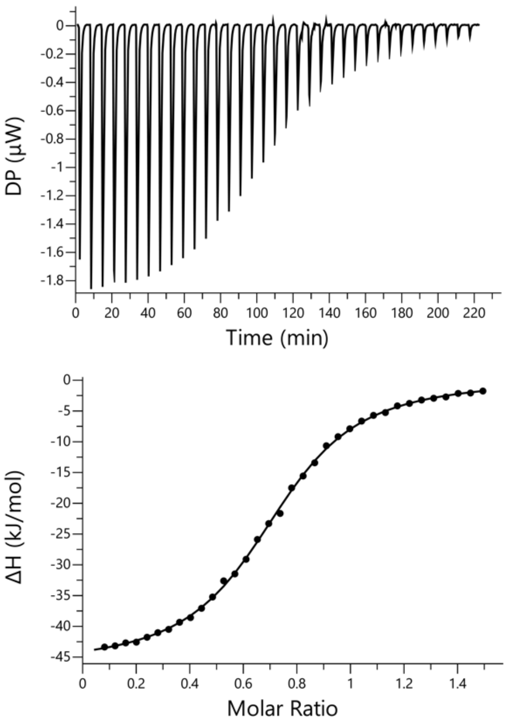

Figure S35. *ITC data for titration of* ***9*** *(0.28 mM) into* ***3*** *(0.04 mM) in water at 298 K. The raw data for each injection is shown (differential power, DP), along with the least-squares-fit of the enthalpy change per mole of guest (ΔH) to a 1:1 binding isotherm.*

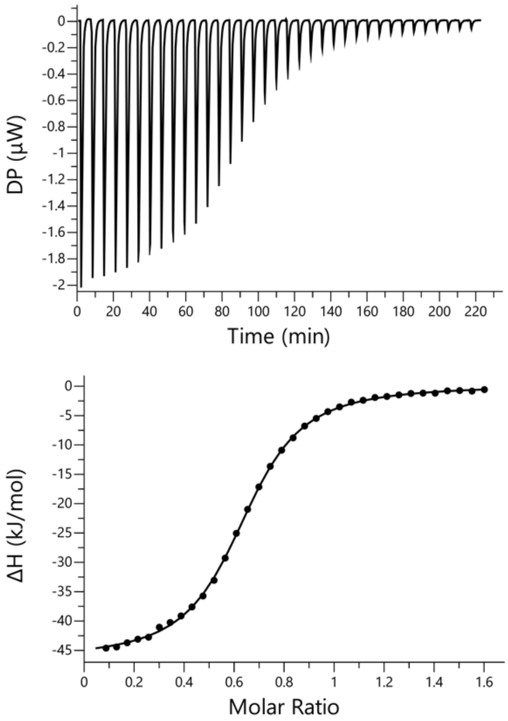

Figure S36. *ITC data for titration of* ***10*** *(0.30 mM) into* ***3*** *(0.04 mM) in water at 298 K. The raw data for each injection is shown (differential power, DP), along with the least-squares-fit of the enthalpy change per mole of guest (ΔH) to a 1:1 binding isotherm.*

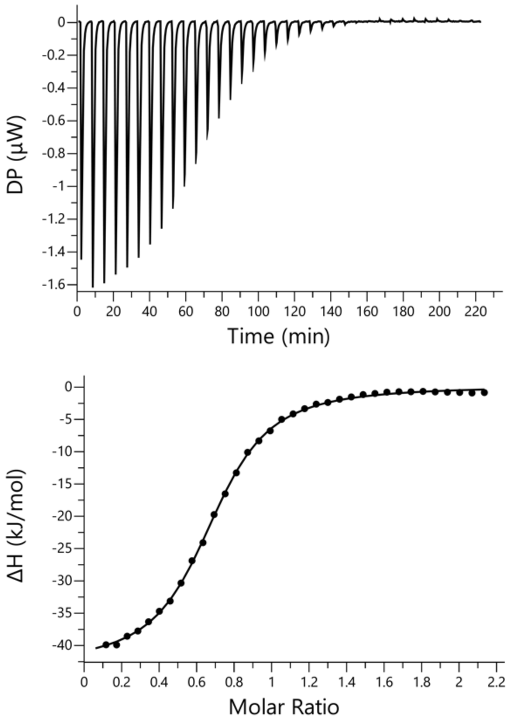

Figure S37. *ITC data for titration of* ***11*** *(0.30 mM) into* ***3*** *(0.03 mM) in water at 298 K. The raw data for each injection is shown (differential power, DP), along with the least-squares-fit of the enthalpy change per mole of guest (ΔH) to a 1:1 binding isotherm.*

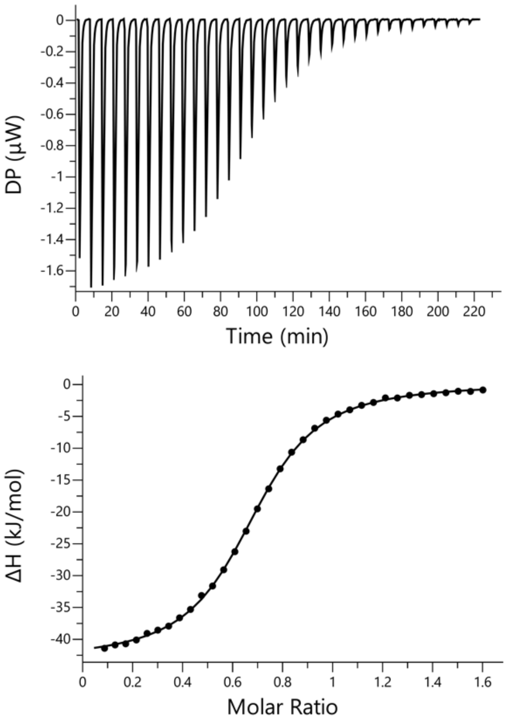

Figure S38. *ITC data for titration of* ***12*** *(0.30 mM) into* ***3*** *(0.04 mM) in water at 298 K. The raw data for each injection is shown (differential power, DP), along with the least-squares-fit of the enthalpy change per mole of guest (ΔH) to a 1:1 binding isotherm.*

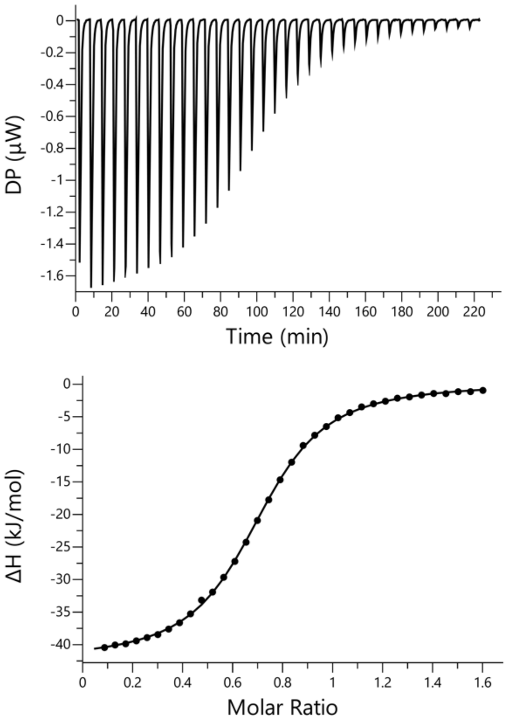

Figure S39. *ITC data for titration of* ***13*** *(0.30 mM) into* ***3*** *(0.04 mM) in water at 298 K. The raw data for each injection is shown (differential power, DP), along with the least-squares-fit of the enthalpy change per mole of guest (ΔH) to a 1:1 binding isotherm.*

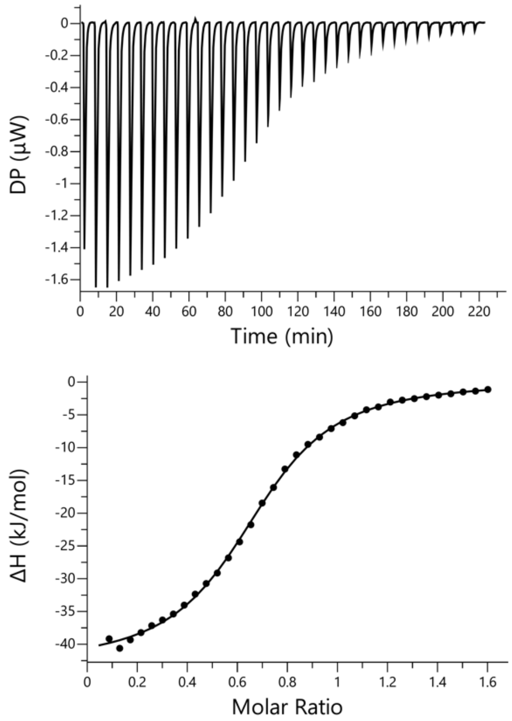

Figure S40. *ITC data for titration of **14** (0.30 mM) into **3** (0.04 mM) in water at 298 K. The raw data for each injection is shown (differential power, DP), along with the least-squares-fit of the enthalpy change per mole of guest (ΔH) to a 1:1 binding isotherm.*

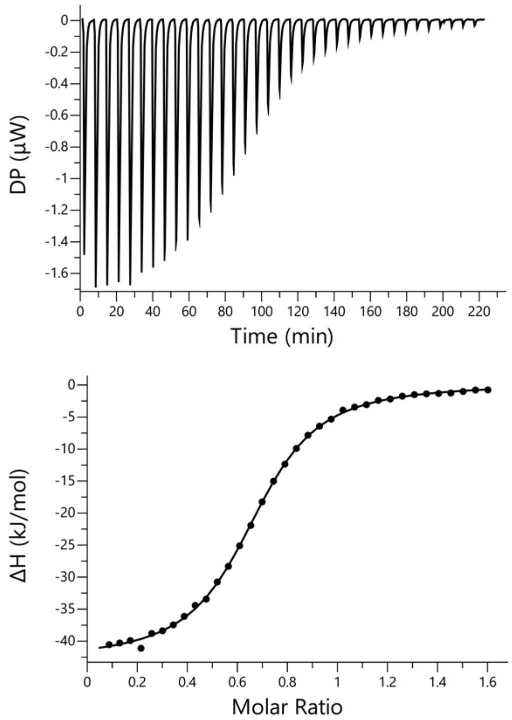

Figure S41. *ITC data for titration of **15** (0.30 mM) into **3** (0.04 mM) in water at 298 K. The raw data for each injection is shown (differential power, DP), along with the least-squares-fit of the enthalpy change per mole of guest (ΔH) to a 1:1 binding isotherm.*

3.3. Octachloro-super-aryl-extended calix[4]pyrrole 2

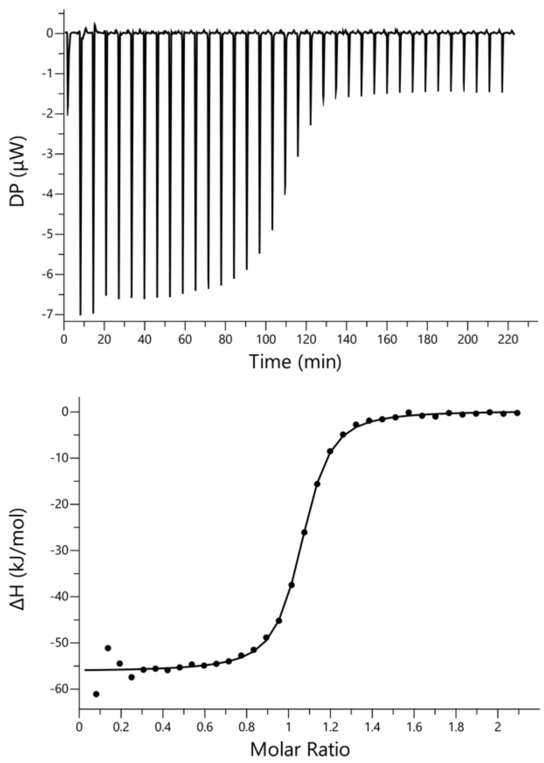

__Figure S42.__ ITC data for titration of ***PNO*** *(0.30 mM) into* ***2*** *(0.03 mM) in chloroform at 298 K. The raw data for each injection is shown (differential power, DP), along with the least-squares-fit of the enthalpy change per mole of guest (ΔH) to a 1:1 binding isotherm.*

3.4. Tetrachloro-aryl-extended calix[4]pyrrole 4

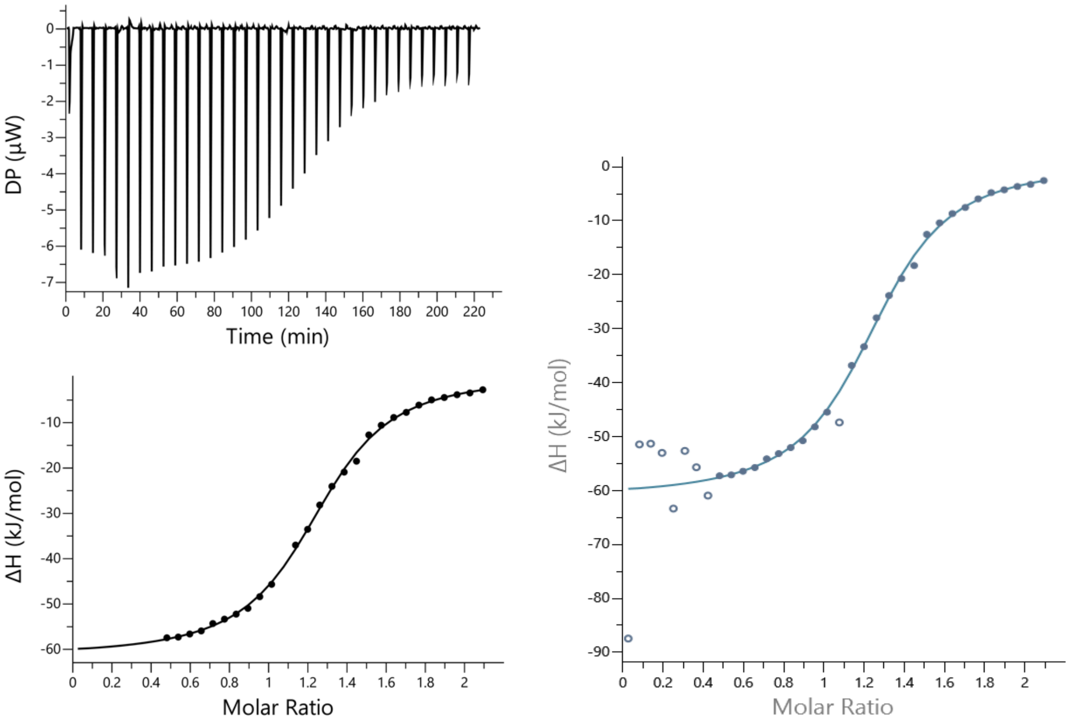

Figure S43. *ITC data for titration of* ***PNO*** *(0.30 mM) into* ***4*** *(0.03 mM) in chloroform at 298 K. The raw data for each injection is shown (differential power, DP), along with the least-squares-fit of the enthalpy change per mole of guest (ΔH) to a 1:1 binding isotherm. Open circles (right) indicate injections that were removed to fit the data.*

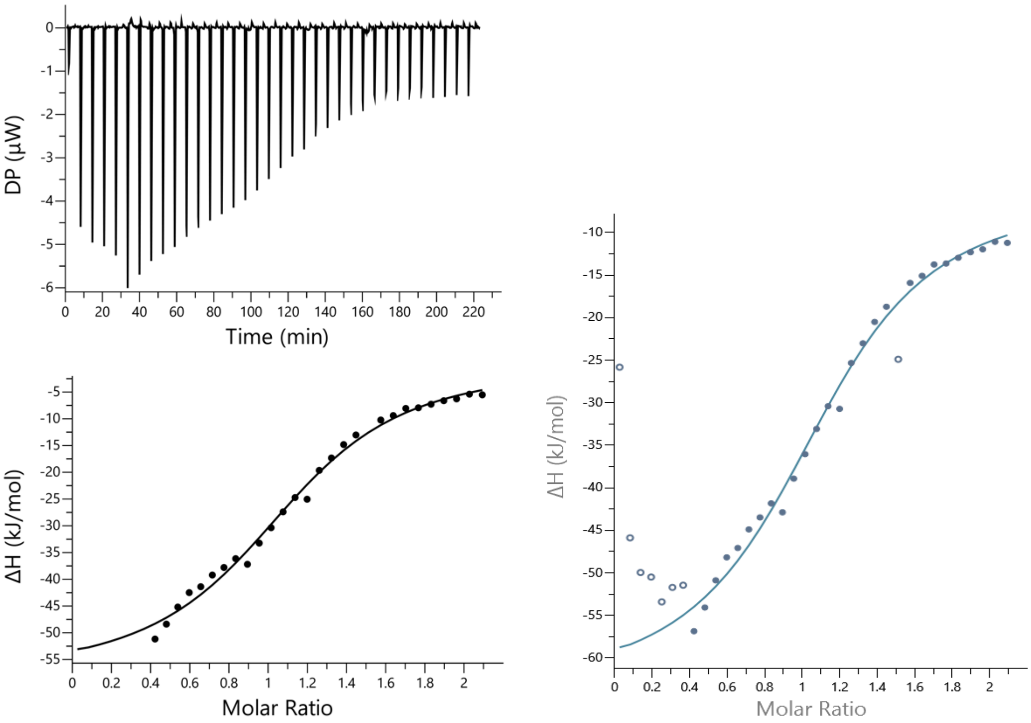

Figure S44. *ITC data for titration of* ***5*** *(0.30 mM) into* ***4*** *(0.03 mM) in chloroform at 298 K. The raw data for each injection is shown (differential power, DP), along with the least-squares-fit of the enthalpy change per mole of guest (ΔH) to a 1:1 binding isotherm. Open circles (right) indicate injections that were removed to fit the data.*

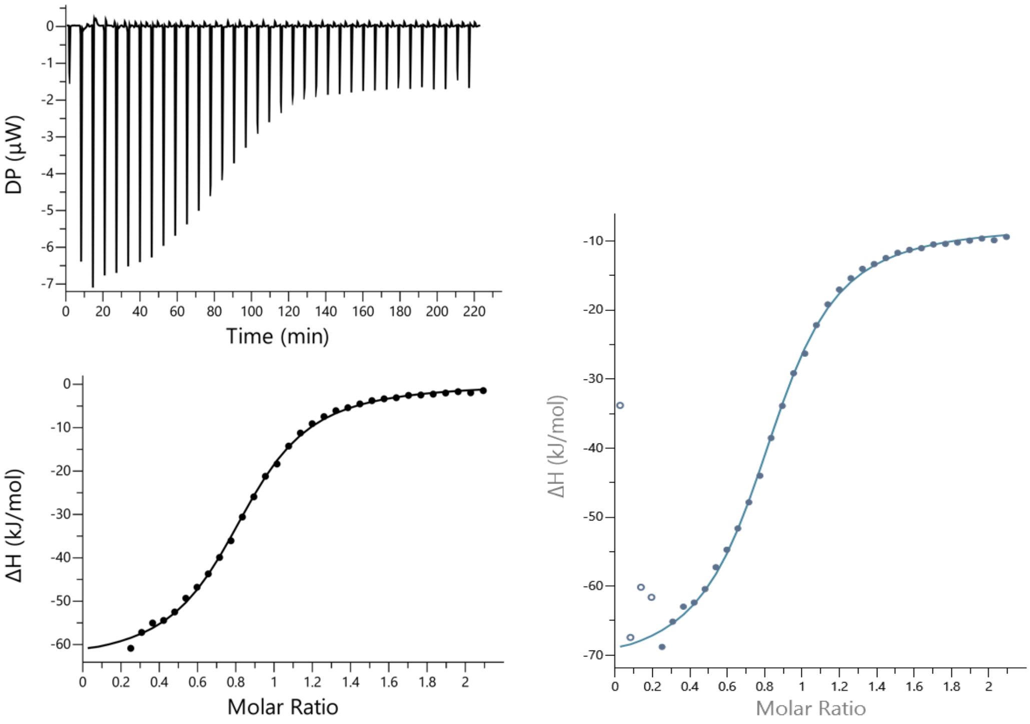

Figure S45. *ITC data for titration of **6** (0.30 mM) into **4** (0.03 mM) in chloroform at 298 K. The raw data for each injection is shown (differential power, DP), along with the least-squares-fit of the enthalpy change per mole of guest (ΔH) to a 1:1 binding isotherm. Open circles (right) indicate injections that were removed to fit the data.*

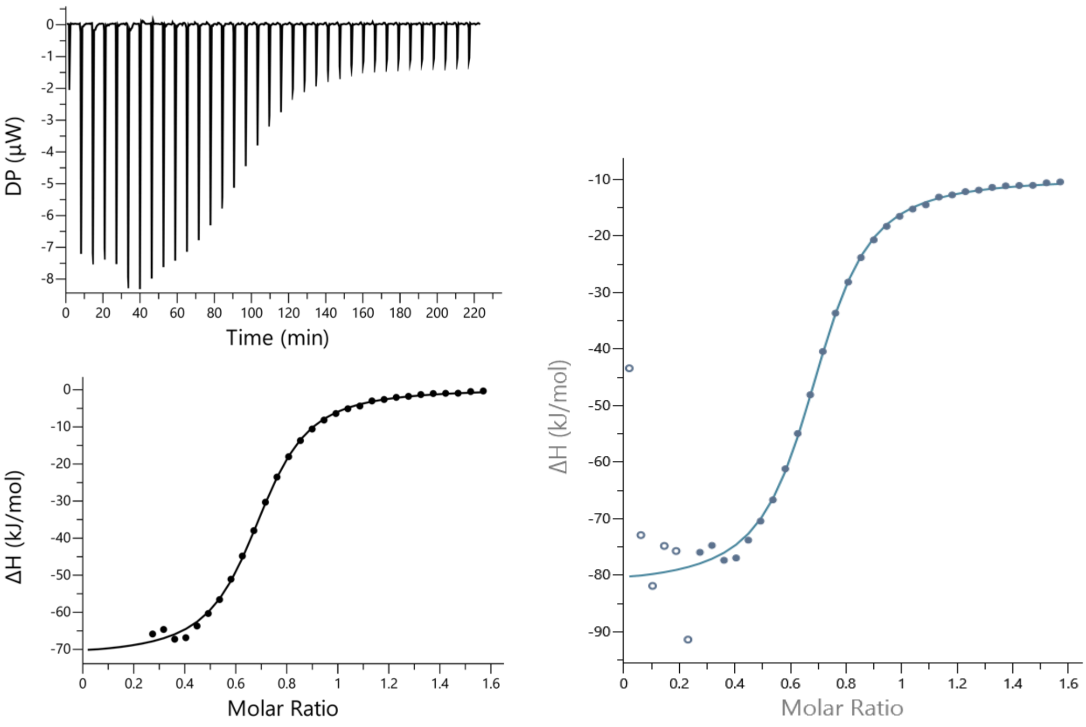

Figure S46. *ITC data for titration of **7** (0.30 mM) into **4** (0.04 mM) in chloroform at 298 K. The raw data for each injection is shown (differential power, DP), along with the least-squares-fit of the enthalpy change per mole of guest (ΔH) to a 1:1 binding isotherm. Open circles (right) indicate injections that were removed to fit the data.*

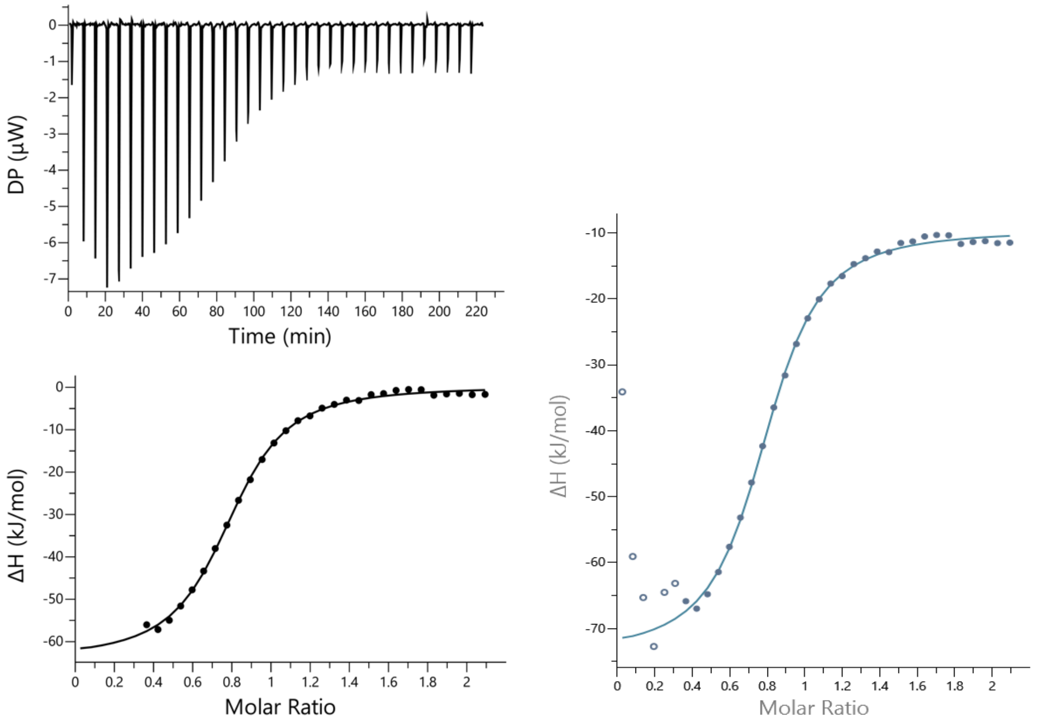

Figure S47. *ITC data for titration of* ***8*** *(0.30 mM) into* ***4*** *(0.03 mM) in chloroform at 298 K. The raw data for each injection is shown (differential power, DP), along with the least-squares-fit of the enthalpy change per mole of guest (ΔH) to a 1:1 binding isotherm. Open circles (right) indicate injections that were removed to fit the data.*

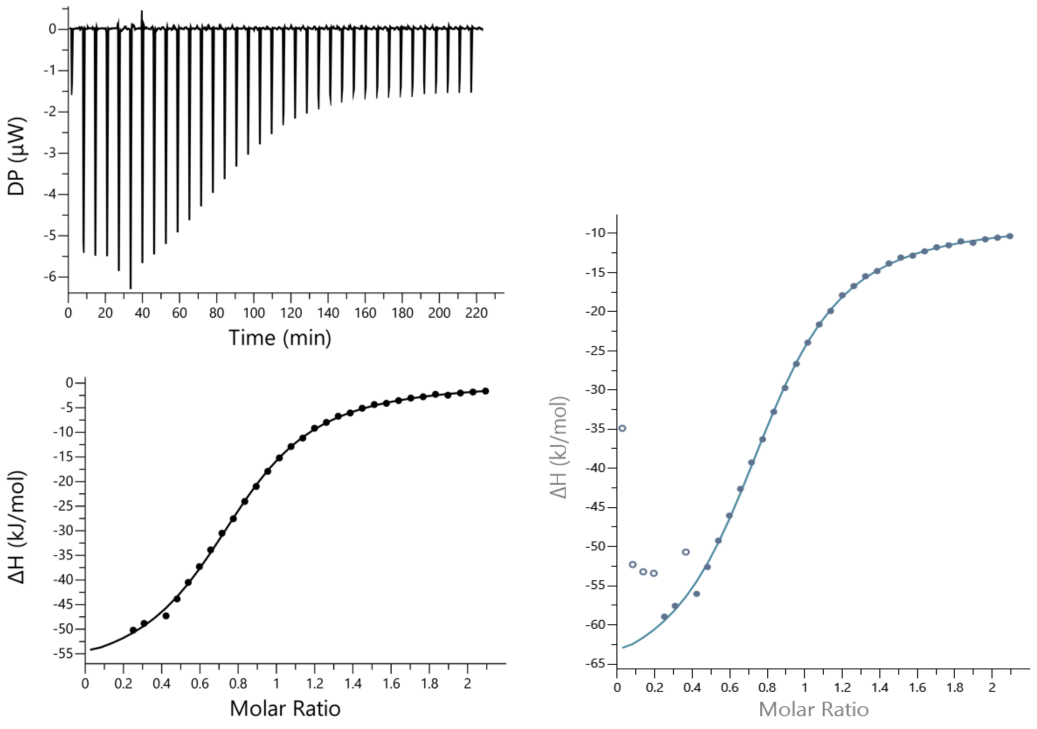

Figure S48. *ITC data for titration of* ***9*** *(0.30 mM) into* ***4*** *(0.03 mM) in chloroform at 298 K. The raw data for each injection is shown (differential power, DP), along with the least-squares-fit of the enthalpy change per mole of guest (ΔH) to a 1:1 binding isotherm. Open circles (right) indicate injections that were removed to fit the data.*

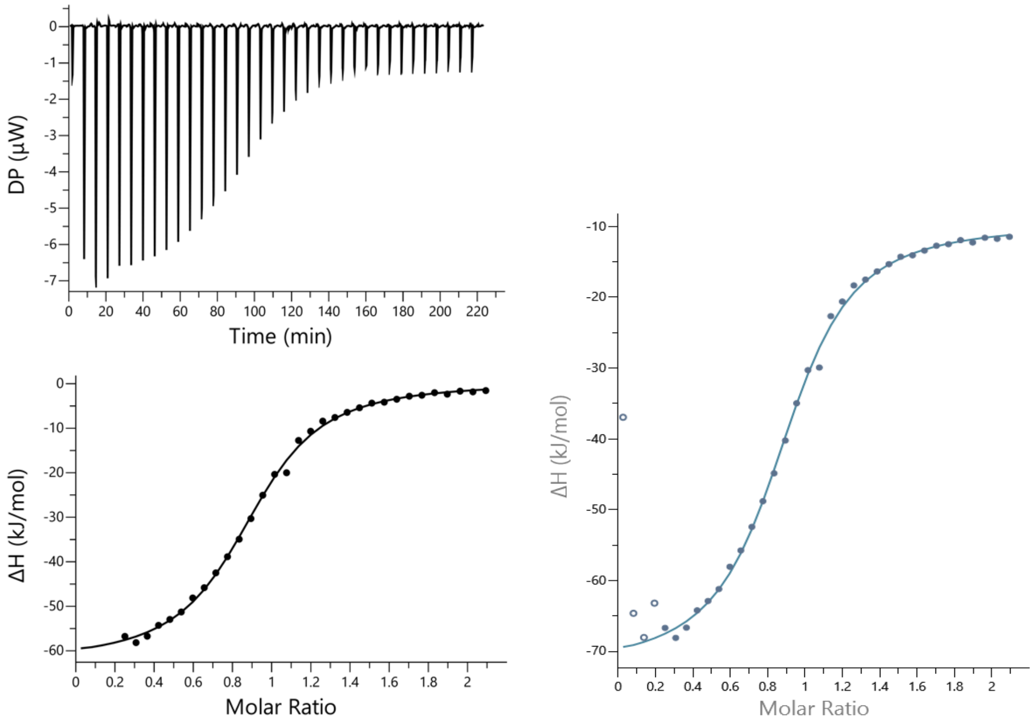

Figure S49. *ITC data for titration of* ***10*** *(0.30 mM) into* ***4*** *(0.03 mM) in chloroform at 298 K. The raw data for each injection is shown (differential power, DP), along with the least-squares-fit of the enthalpy change per mole of guest (ΔH) to a 1:1 binding isotherm. Open circles (right) indicate injections that were removed to fit the data.*

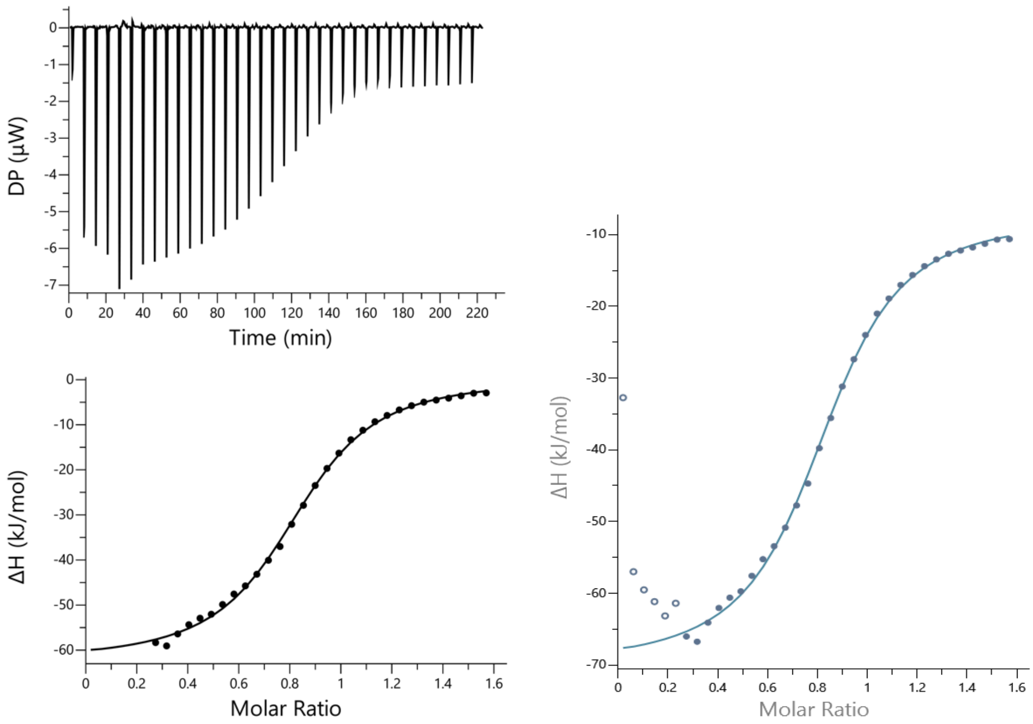

Figure S50. *ITC data for titration of* ***11*** *(0.30 mM) into* ***4*** *(0.04 mM) in chloroform at 298 K. The raw data for each injection is shown (differential power, DP), along with the least-squares-fit of the enthalpy change per mole of guest (ΔH) to a 1:1 binding isotherm. Open circles (right) indicate injections that were removed to fit the data.*

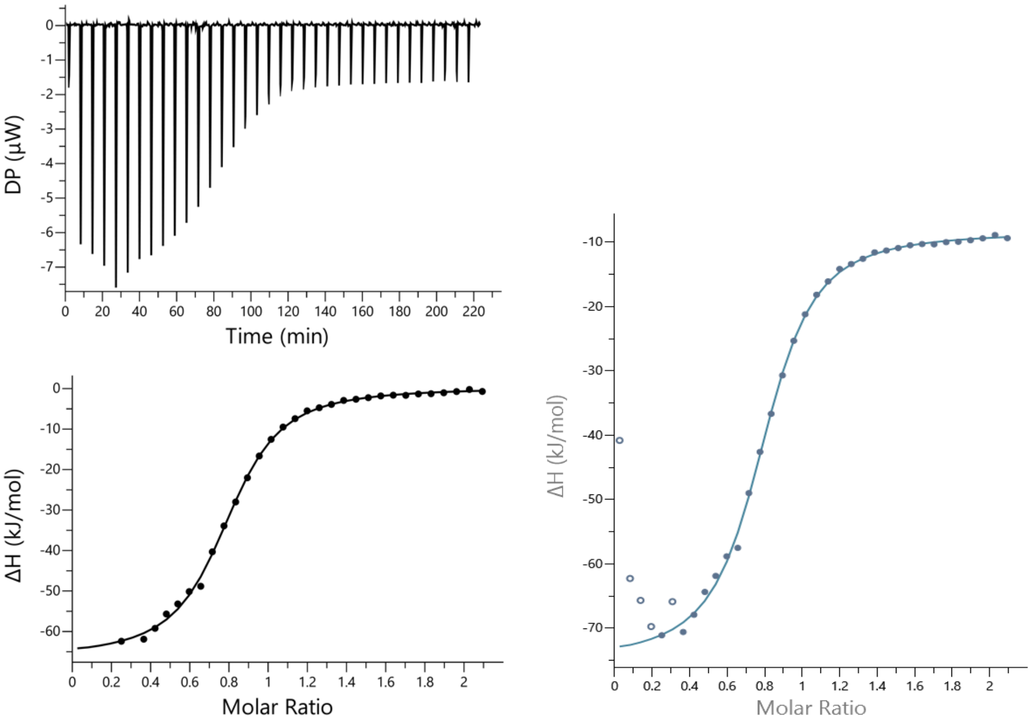

Figure S51. *ITC data for titration of **12** (0.30 mM) into **4** (0.03 mM) in chloroform at 298 K. The raw data for each injection is shown (differential power, DP), along with the least-squares-fit of the enthalpy change per mole of guest (ΔH) to a 1:1 binding isotherm. Open circles (right) indicate injections that were removed to fit the data.*

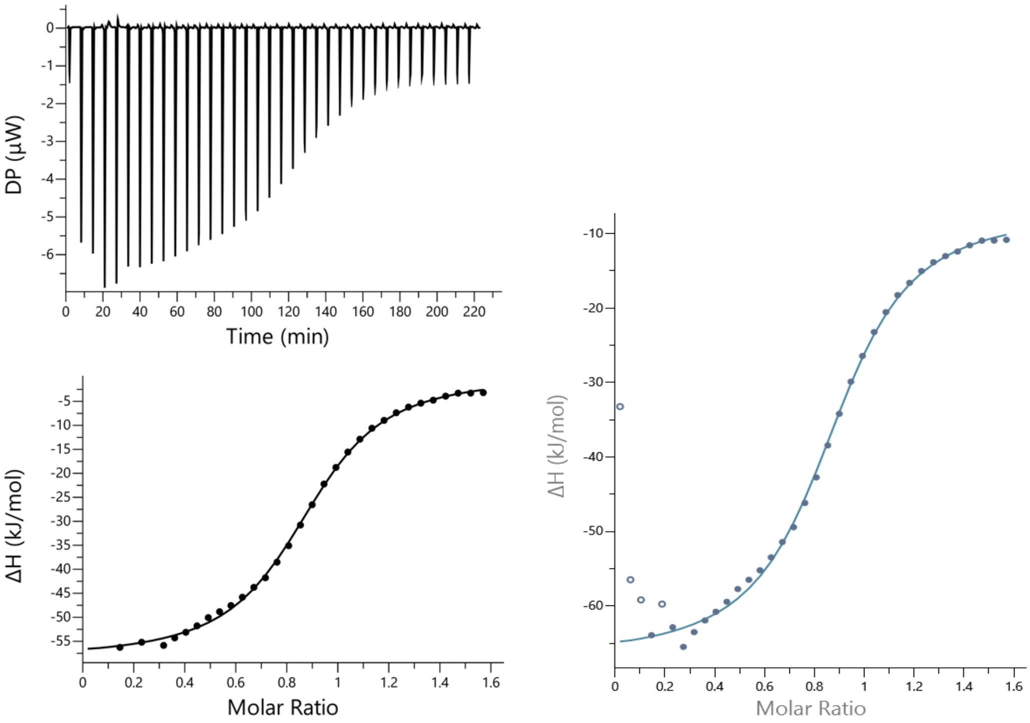

Figure S52. *ITC data for titration of **13** (0.30 mM) into **4** (0.04 mM) in chloroform at 298 K. The raw data for each injection is shown (differential power, DP), along with the least-squares-fit of the enthalpy change per mole of guest (ΔH) to a 1:1 binding isotherm. Open circles (right) indicate injections that were removed to fit the data.*

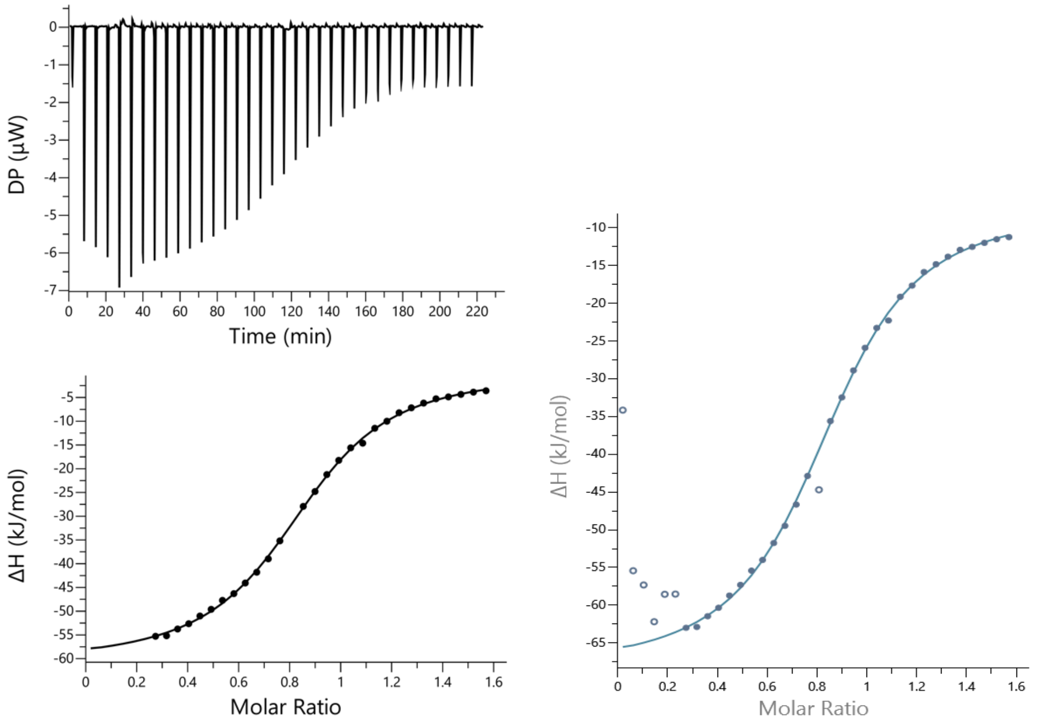

Figure S53. *ITC data for titration of **14** (0.30 mM) into **4** (0.04 mM) in chloroform at 298 K. The raw data for each injection is shown (differential power, DP), along with the least-squares-fit of the enthalpy change per mole of guest (ΔH) to a 1:1 binding isotherm. Open circles (right) indicate injections that were removed to fit the data.*

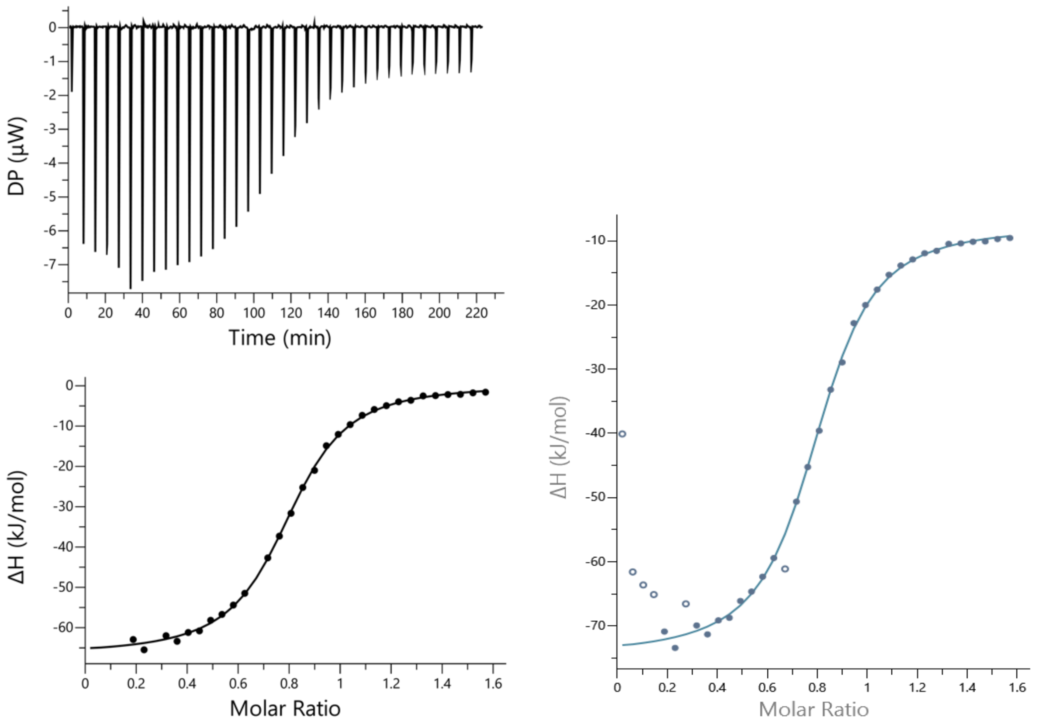

Figure S54. *ITC data for titration of **15** (0.30 mM) into **4** (0.04 mM) in chloroform at 298 K. The raw data for each injection is shown (differential power, DP), along with the least-squares-fit of the enthalpy change per mole of guest (ΔH) to a 1:1 binding isotherm. Open circles (right) indicate injections that were removed to fit the data.*

4. Pairwise ¹H NMR competitive titrations

Competitive titration experiments were performed using calix[4]pyrroles **1** and **2**, and pyridine *N*-oxides **5-15** in non-buffered deuterium oxide and deuterochloroform solutions. The association constant ratios between two competing complexes were determined by integrating selected proton signals in the acquired ¹H NMR spectra.

4.1. Octapyridinium-super-aryl-extended calix[4]pyrrole 1

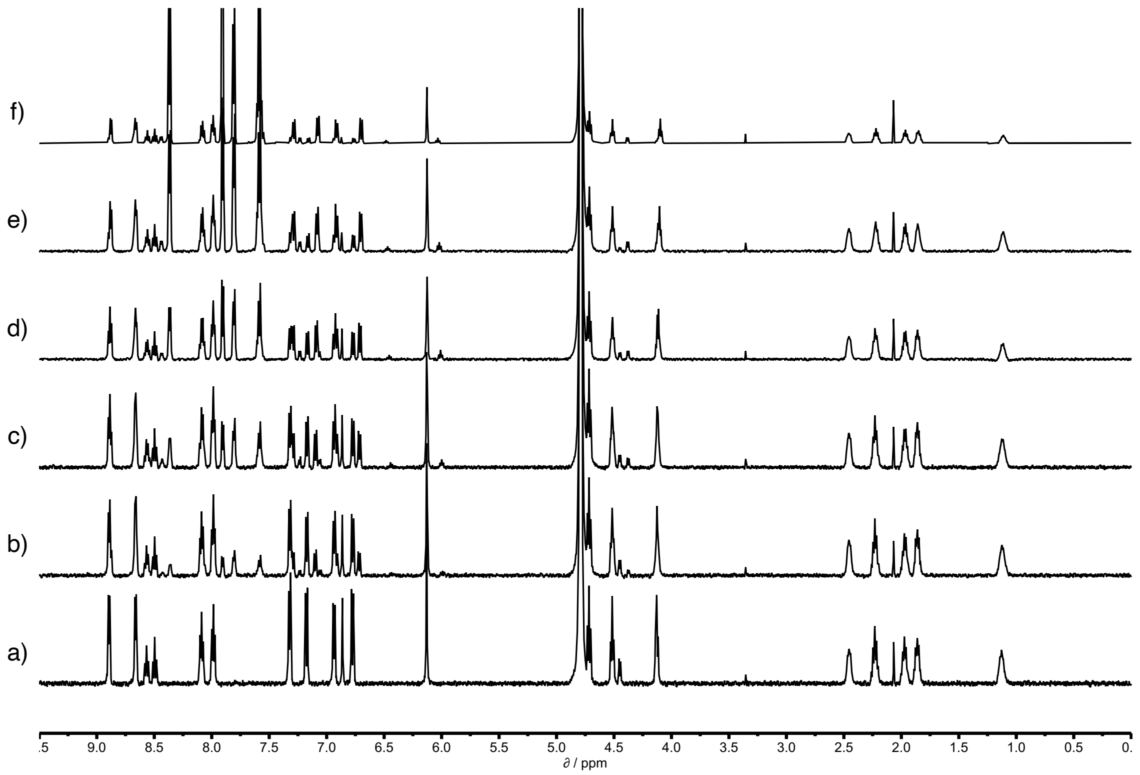

Figure S55. *500MHz ¹H NMR for titration of **PPNO** into a mixture of **1** and **5** in D_2O at 298K. Concentrations are: a) **1**: 0.36 mM; **PPNO**: 0 mM; **5**: 0.41 mM; b) **1**: 0.32 mM; **PPNO**: 0.34 mM; **5**: 0.36 mM; c) **1**: 0.28 mM; **PPNO**: 0.60 mM; **5**: 0.33 mM; d) **1**: 0.23 mM; **PPNO**: 1.00 mM; **5**: 0.27 mM; e) **1**: 0.17 mM; **PPNO**: 1.48 mM; **5**: 0.20 mM; f) **1**: 0.11 mM; **PPNO**: 1.95 mM; **5**: 0.13 mM. Integration of selected proton signals indicated that K(**1•5**) = 4.0±0.8 x K(**1•PPNO**).*

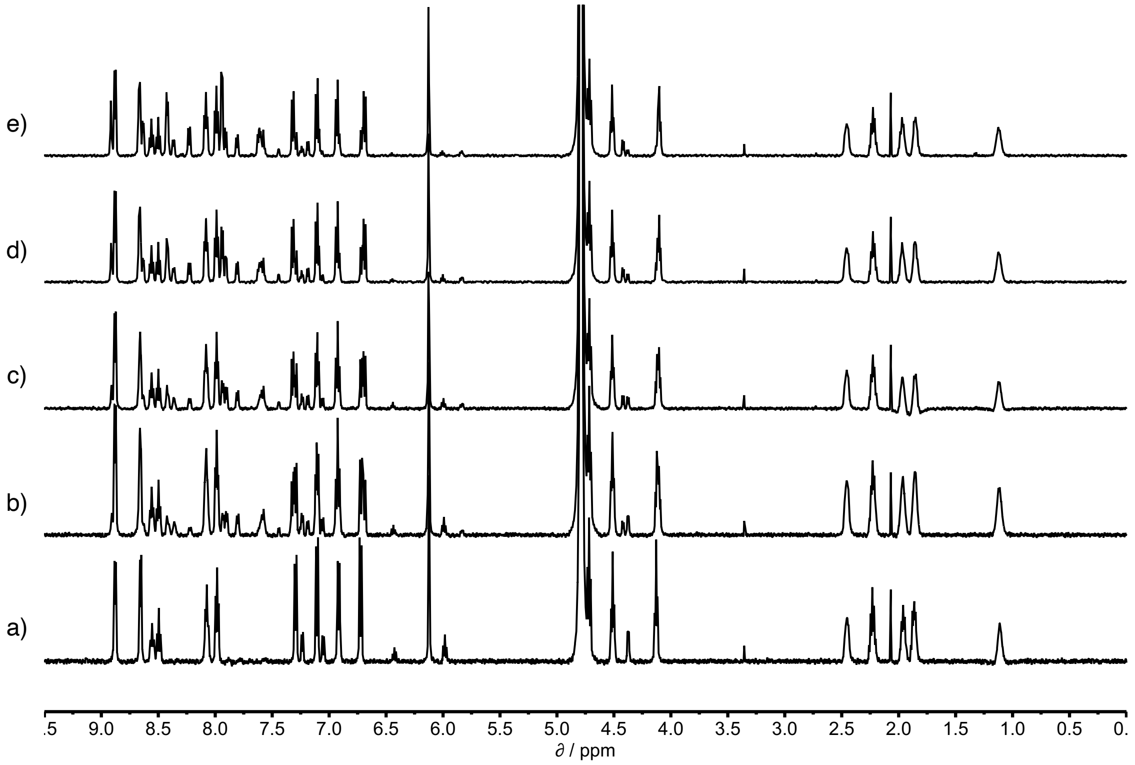

Figure S56. *500MHz ^1H NMR for titration of **6** into a mixture of **1** and **PPNO** in D_2O at 298K. Concentrations are: a) **1**: 0.38 mM; **PPNO**: 0.44 mM; **6**: 0 mM; b) **1**: 0.33 mM; **PPNO**: 0.38 mM; **6**: 0.33 mM; c) **1**: 0.31 mM; **PPNO**: 0.36 mM; **6**: 0.47 mM; d) **1**: 0.27 mM; **PPNO**: 0.32 mM; **6**: 0.69 mM; e) **1**: 0.25 mM; **PPNO**: 0.29 mM; **6**: 0.87 mM. Integration of selected proton signals indicated that K(**1•6**) = 0.8±0.1 x K(**1•PPNO**).*

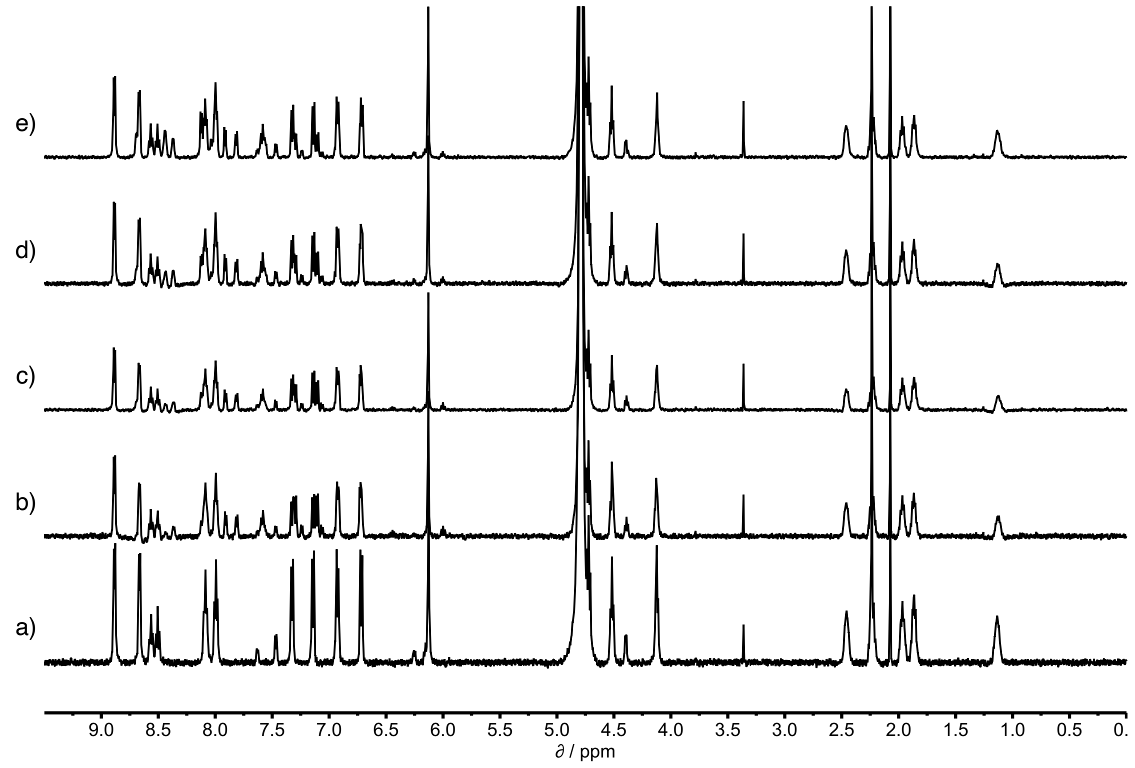

Figure S57. *500MHz ^1H NMR for titration of **7** into a mixture of **1, PPNO** and **7** in D_2O at 298K. Concentrations are: a) **1**: 0.36 mM; **PPNO**: 0 mM; **7**: 0.39 mM; b) **1**: 0.30 mM; **PPNO**: 0.39 mM; **7**: 0.32 mM; c) **1**: 0.28 mM; **PPNO**: 0.37 mM; **7**: 0.40 mM; d) **1**: 0.26 mM; **PPNO**: 0.35 mM; **7**: 0.47 mM; e) **1**: 0.24 mM; **PPNO**: 0.31 mM; **7**: 0.60 mM. Integration of selected proton signals indicated that K(**1•7**) = 1.2±0.2 x K(**1•PPNO**).*

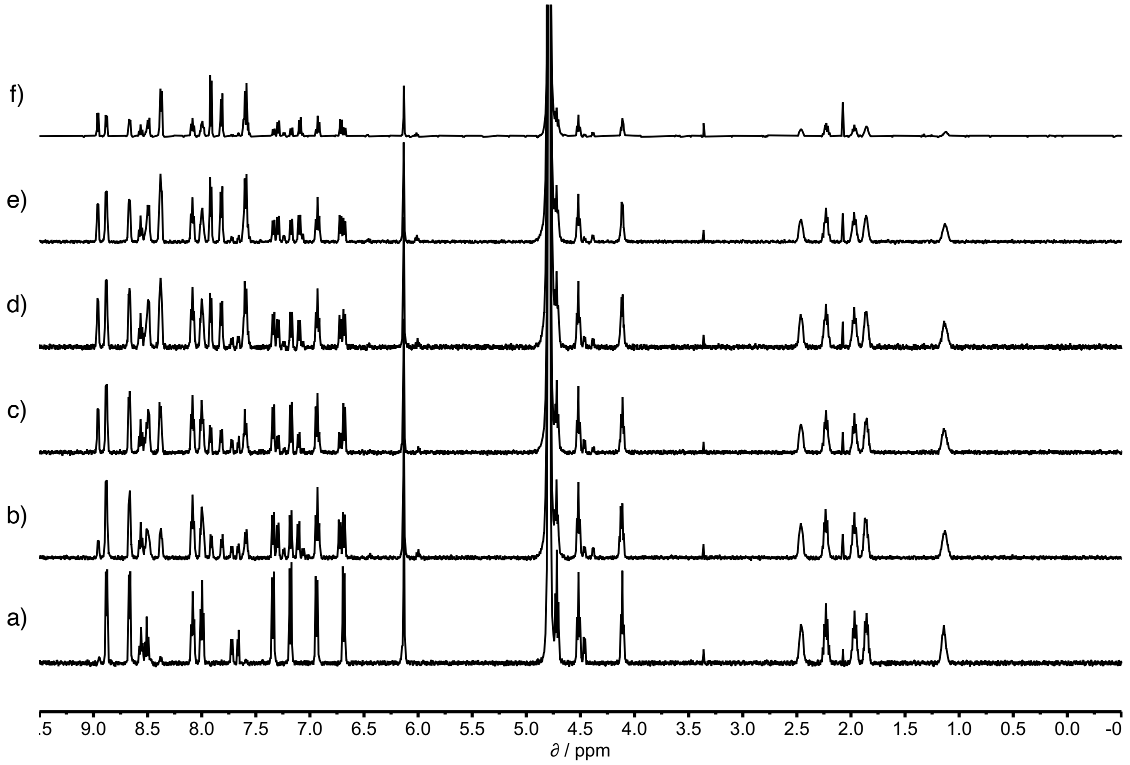

Figure S58. *500MHz ^{1}H NMR for titration of **PPNO** into a mixture of **1** and **8** in D_2O at 298K. Concentrations are: a) **1**: 0.40 mM; **PPNO**: 0 mM; **8**: 0.48 mM; b) **1**: 0.32 mM; **PPNO**: 0.39 mM; **8**: 0.39 mM; c) **1**: 0.25 mM; **PPNO**: 0.31 mM; **8**: 0.61 mM; d) **1**: 0.22 mM; **PPNO**: 0.45 mM; **8**: 0.53 mM; e) **1**: 0.20 mM; **PPNO**: 0.71 mM; **8**: 0.47 mM; f) **1**: 0.16 mM; **PPNO**: 0.97 mM; **8**: 0.38 mM. Integration of selected proton signals indicated that K(**1•8**) = 1.5±0.2 x K(**1•PPNO**).*

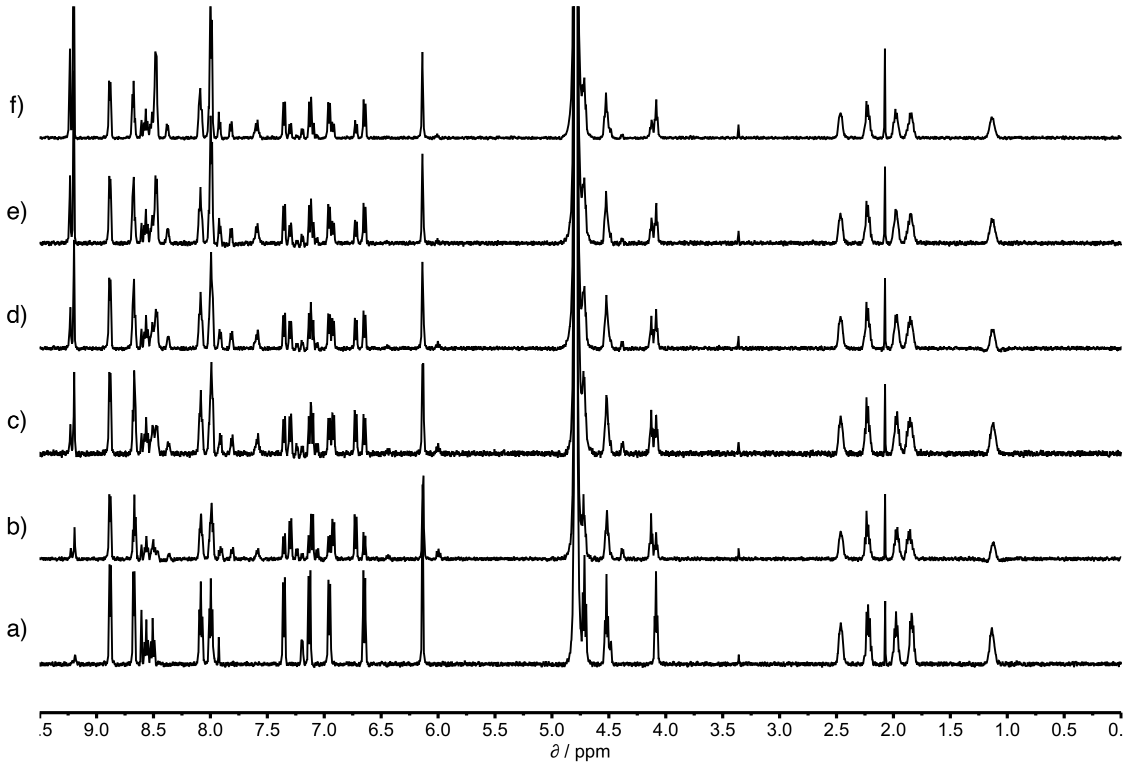

Figure S59. *500MHz ^{1}H NMR for titration of **9** into a mixture of **1**, **PPNO** and **9** in D_2O at 298K. Concentrations are: a) **1**: 0.37 mM; **PPNO**: 0 mM; **9**: 0.46 mM; b) **1**: 0.31 mM; **PPNO**: 0.37 mM; **9**: 0.38 mM; c) **1**: 0.27 mM; **PPNO**: 0.33 mM; **9**: 0.56 mM; d) **1**: 0.25 mM; **PPNO**: 0.30 mM; **9**: 0.71 mM; e) **1**: 0.21 mM; **PPNO**: 0.25 mM; **9**: 0.94 mM; f) **1**: 0.18 mM; **PPNO**: 0.22 mM; **9**: 1.11 mM. Integration of selected proton signals indicated that K(**1•PPNO**) = 2.5±0.2 x K(**1•9**).*

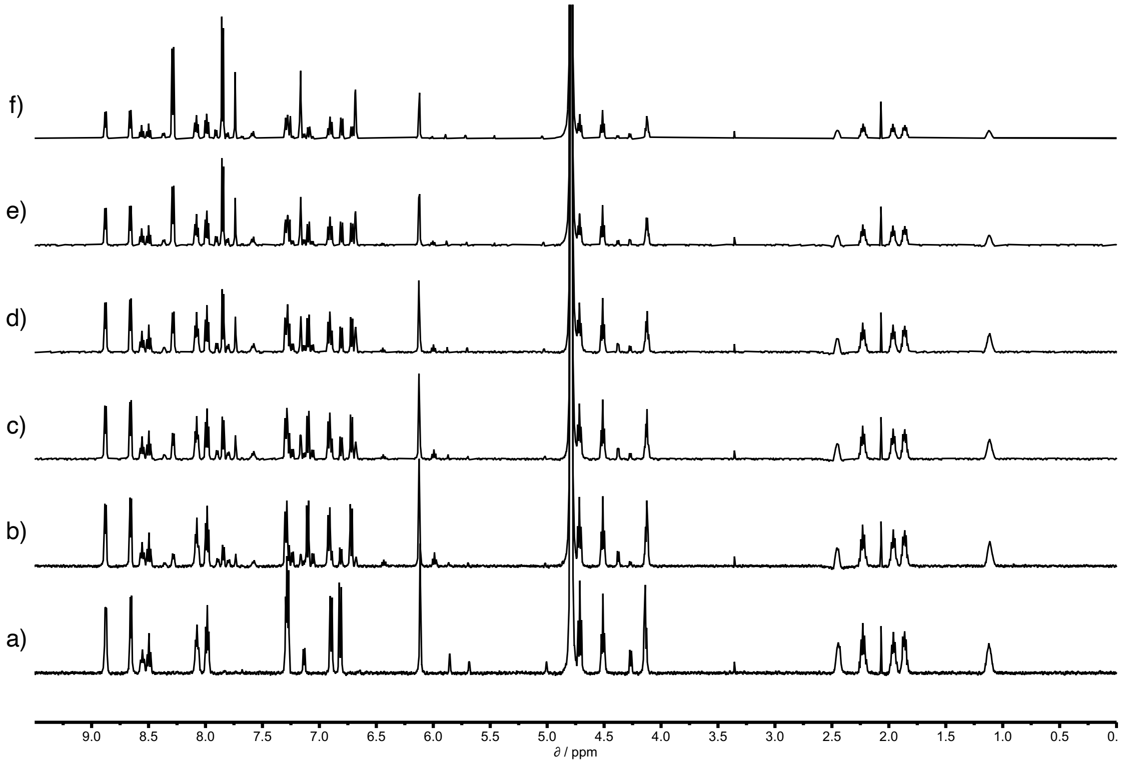

Figure S60. *500MHz* ^{1}H *NMR for titration of* ***10*** *into a mixture of* ***1***, ***PPNO*** *and* ***10*** *in* D_2O *at 298K. Concentrations are: a)* ***1****: 0.38 mM;* ***PPNO****: 0 mM;* ***10****: 0.44 mM; b)* ***1****: 0.33 mM;* ***PPNO****: 0.38 mM;* ***10****: 0.38 mM; c)* ***1****: 0.29 mM;* ***PPNO****: 0.34 mM;* ***10****: 0.67 mM; d)* ***1****: 0.26 mM;* ***PPNO****: 0.30 mM;* ***10****: 0.89 mM; e)* ***1****: 0.21 mM;* ***PPNO****: 0.25 mM;* ***10****: 1.23 mM; f)* ***1****: 0.16 mM;* ***PPNO****: 0.19 mM;* ***10****: 1.64 mM. Integration of selected proton signals indicated that K(****1•PPNO****) = 7.8±0.9 x K(****1•10****).*

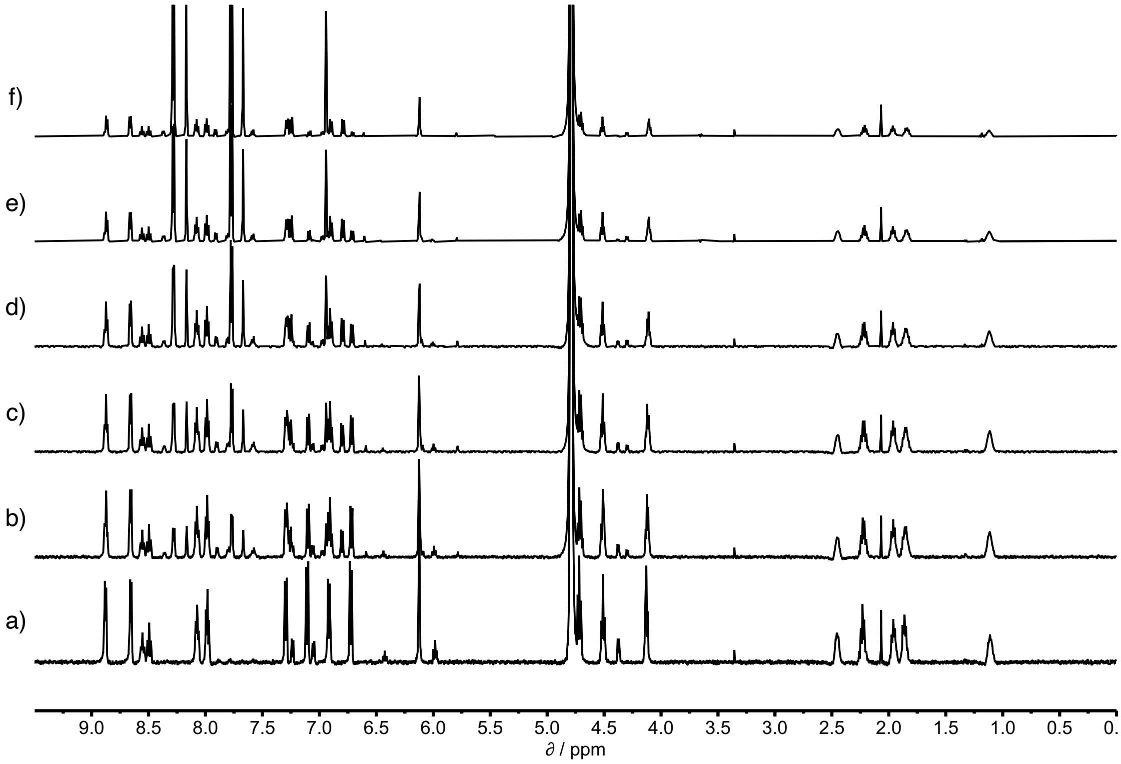

Figure S61. *500MHz* ^{1}H *NMR for titration of* ***11*** *into a mixture of* ***1*** *and* ***PPNO*** *in* D_2O *at 298K. Concentrations are: a)* ***1****: 0.38 mM;* ***PPNO****: 0.44 mM;* ***11****: 0 mM; b)* ***1****: 0.31 mM;* ***PPNO****: 0.36 mM;* ***11****: 0.60 mM; c)* ***1****: 0.27 mM;* ***PPNO****: 0.32 mM;* ***11****: 0.89 mM; d)* ***1****: 0.22 mM;* ***PPNO****: 0.26 mM;* ***11****: 1.31 mM; e)* ***1****: 0.16 mM;* ***PPNO****: 0.19 mM;* ***11****: 1.81 mM; f)* ***1****: 0.11 mM;* ***PPNO****: 0.12 mM;* ***11****: 2.30 mM. Integration of selected proton signals indicated that K(****1•PPNO****) = 5.9±0.5 x K(****1•11****).*

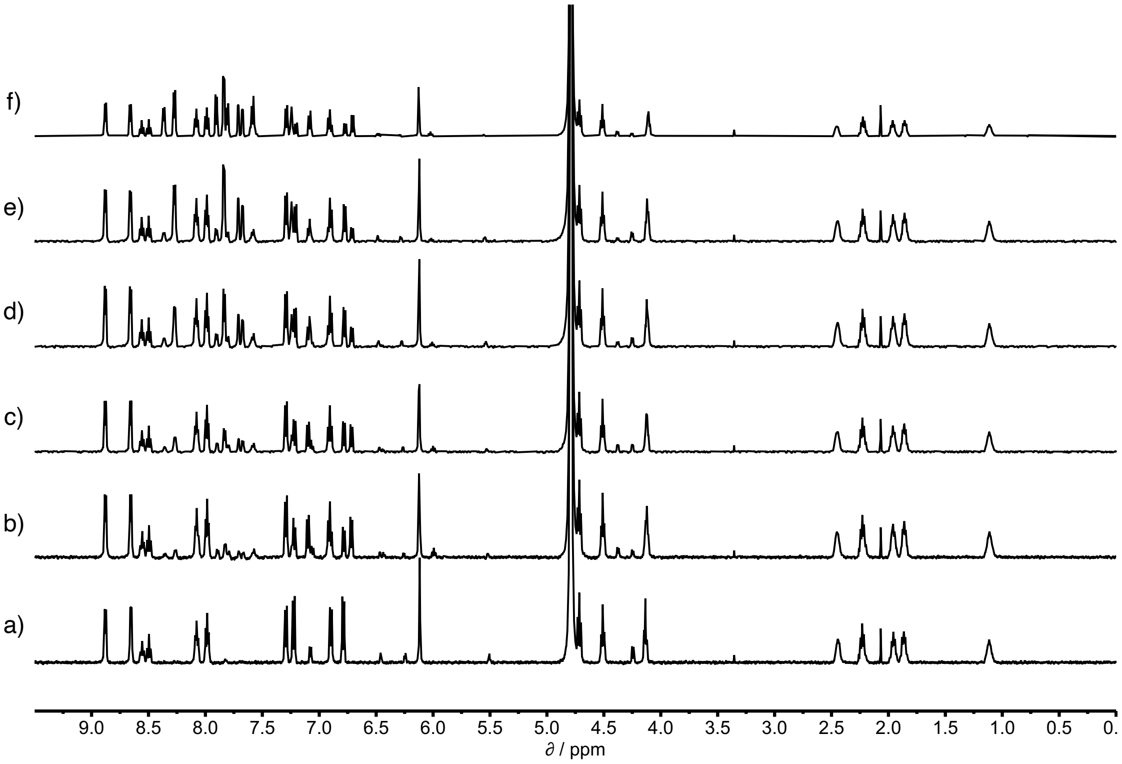

Figure S62. *500MHz ^{1}H NMR for titration of* ***PPNO*** *into a mixture of* ***1*** *and* ***12*** *in D_2O at 298K. Concentrations are: a)* ***1****: 0.36 mM;* ***PPNO****: 0 mM;* ***12****: 0.48 mM; b)* ***1****: 0.31 mM;* ***PPNO****: 0.34 mM;* ***12****: 0.37 mM; c)* ***1****: 0.28 mM;* ***PPNO****: 0.30 mM;* ***12****: 0.55 mM; d)* ***1****: 0.23 mM;* ***PPNO****: 0.25 mM;* ***12****: 0.82 mM; e)* ***1****: 0.29 mM;* ***PPNO****: 0.21 mM;* ***12****: 1.01 mM; f)* ***1****: 0.16 mM;* ***PPNO****: 0.69 mM;* ***12****: 0.82 mM. Integration of selected proton signals indicated that K(****1•PPNO****) = 2.0±0.2 x K(****1•12****).*

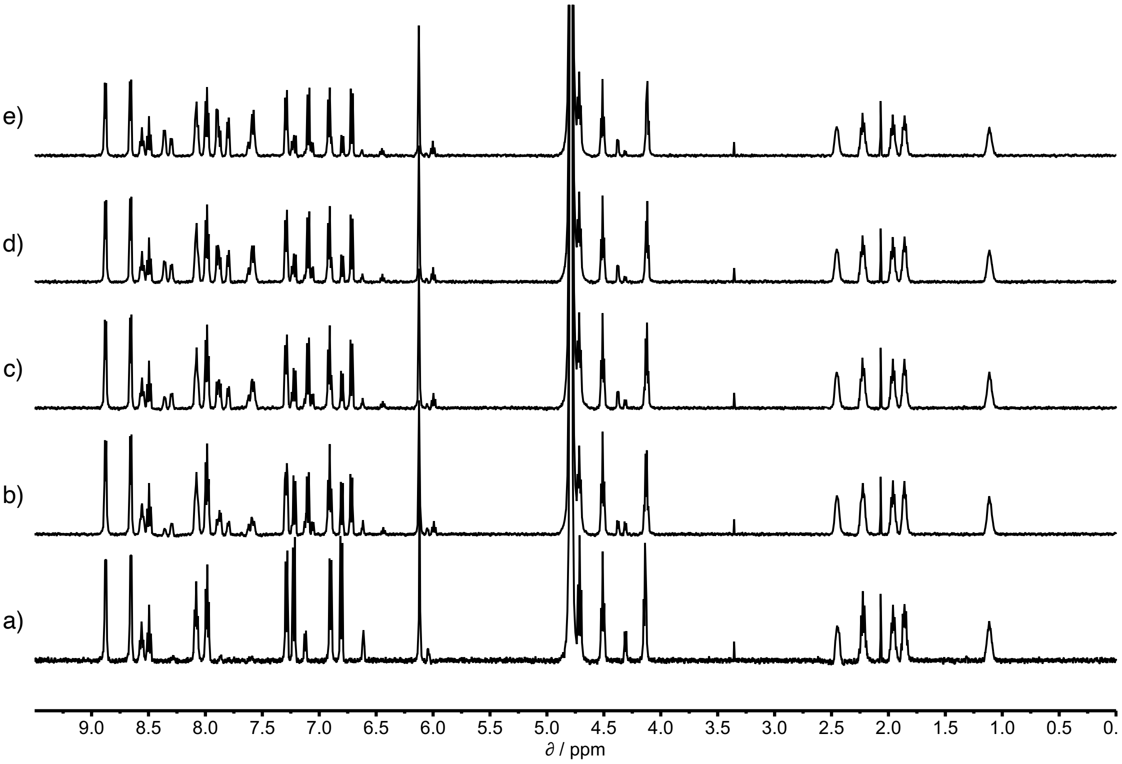

Figure S63. *500MHz ^{1}H NMR for titration of* ***PPNO*** *into a mixture of* ***1*** *and* ***13*** *in D_2O at 298K. Concentrations are: a)* ***1****: 0.36 mM;* ***PPNO****: 0 mM;* ***13****: 0.48 mM; b)* ***1****: 0.32 mM;* ***PPNO****: 0.34 mM;* ***13****: 0.42 mM; c)* ***1****: 0.30 mM;* ***PPNO****: 0.48 mM;* ***13****: 0.40 mM; d)* ***1****: 0.28 mM;* ***PPNO****: 0.60 mM;* ***13****: 0.38 mM; e)* ***1****: 0.27 mM;* ***PPNO****: 0.72 mM;* ***13****: 0.36 mM. Integration of selected proton signals indicated that K(****1•PPNO****) = 1.9±0.1 x K(****1•13****).*

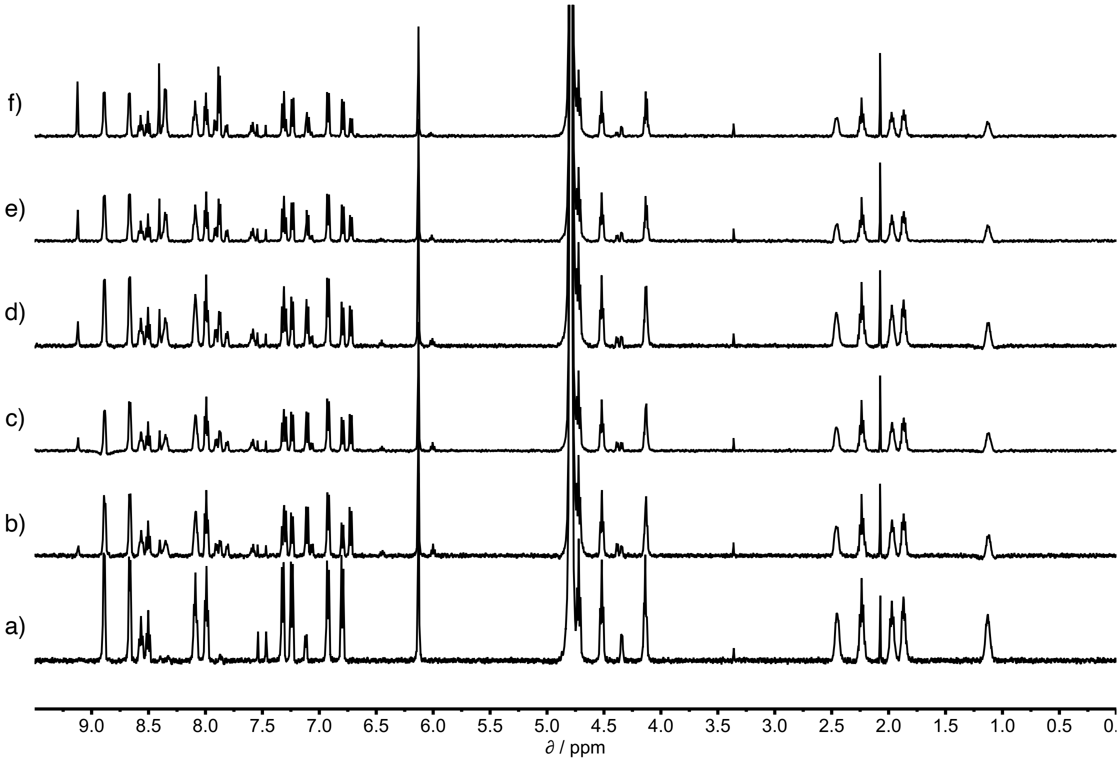

Figure S64. *500MHz ^{1}H NMR for titration of **14** into a mixture of **1**, **PPNO** and **14** in D_2O at 298K. Concentrations are: a) **1**: 0.38 mM; **PPNO**: 0 mM; **14**: 0.46 mM; b) **1**: 0.32 mM; **PPNO**: 0.35 mM; **14**: 0.38 mM; c) **1**: 0.30 mM; **PPNO**: 0.33 mM; **14**: 0.48 mM; d) **1**: 0.28 mM; **PPNO**: 0.31 mM; **14**: 0.57 mM; e) **1**: 0.26 mM; **PPNO**: 0.28 mM; **14**: 0.73 mM; f) **1**: 0.22 mM; **PPNO**: 0.24 mM; **14**: 0.96 mM. Integration of selected proton signals indicated that K(**1•PPNO**) = 2.3±0.1 x K(**1•14**).*

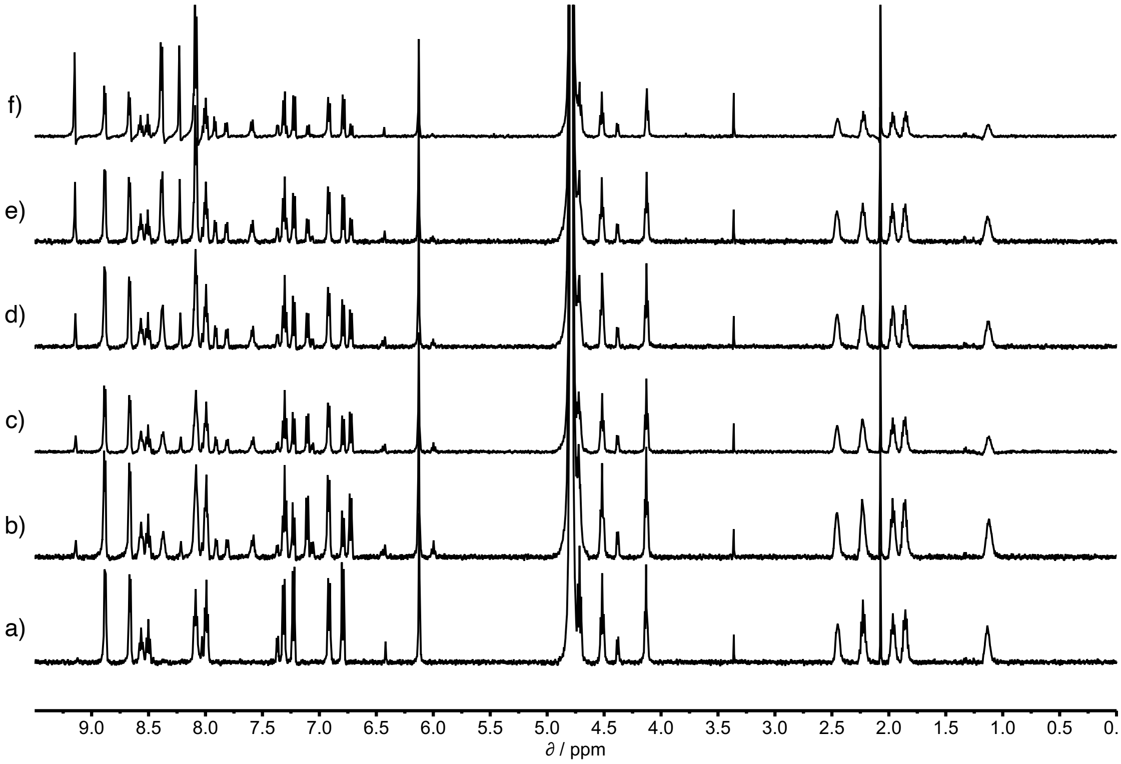

Figure S65. *500MHz ^{1}H NMR for titration of **15** into a mixture of **1**, **PPNO** and **15** in D_2O at 298K. Concentrations are: a) **1**: 0.38 mM; **PPNO**: 0 mM; **15**: 0.44 mM; b) **1**: 0.32 mM; **PPNO**: 0.35 mM; **15**: 0.37 mM; c) **1**: 0.30 mM; **PPNO**: 0.33 mM; **15**: 0.47 mM; d) **1**: 0.27 mM; **PPNO**: 0.30 mM; **15**: 0.64 mM; e) **1**: 0.23 mM; **PPNO**: 0.25 mM; **15**: 0.89 mM; f) **1**: 0.17 mM; **PPNO**: 0.19 mM; **15**: 1.21 mM. Integration of selected proton signals indicated that K(**1•PPNO**) = 2.2±0.2 x K(**1•15**).*

4.2. Octachloro-super-aryl-extended calix[4]pyrrole 2

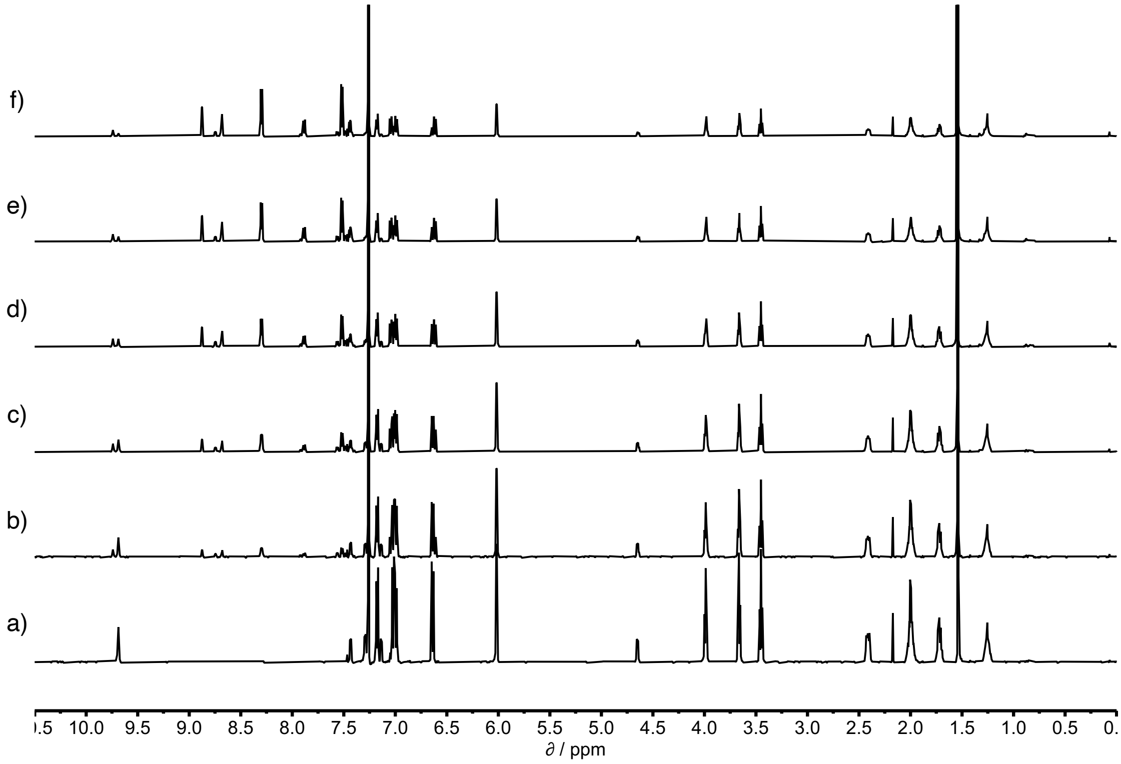

Figure S66. *500MHz ¹H NMR for titration of **6** into a mixture of **1** and **5** in $CDCl_3$ at 298K. Concentrations are: a) **1**: 0.63 mM; **5**: 0.69 mM; **6**: 0 mM; b) **1**: 0.49 mM; **5**: 0.54 mM; **6**: 0.52 mM; c) **1**: 0.40 mM; **5**: 0.44 mM; **6**: 0.85 mM; d) **1**: 0.31 mM; **5**: 0.34 mM; **6**: 1.17 mM; e) **1**: 0.24 mM; **5**: 0.27 mM; **6**: 1.43 mM; f) **1**: 0.19 mM; **5**: 0.21 mM; **6**: 1.62 mM. Integration of selected proton signals indicated that K(**1**•**5**) = 5.0±0.9 x K(**1**• **6**)*

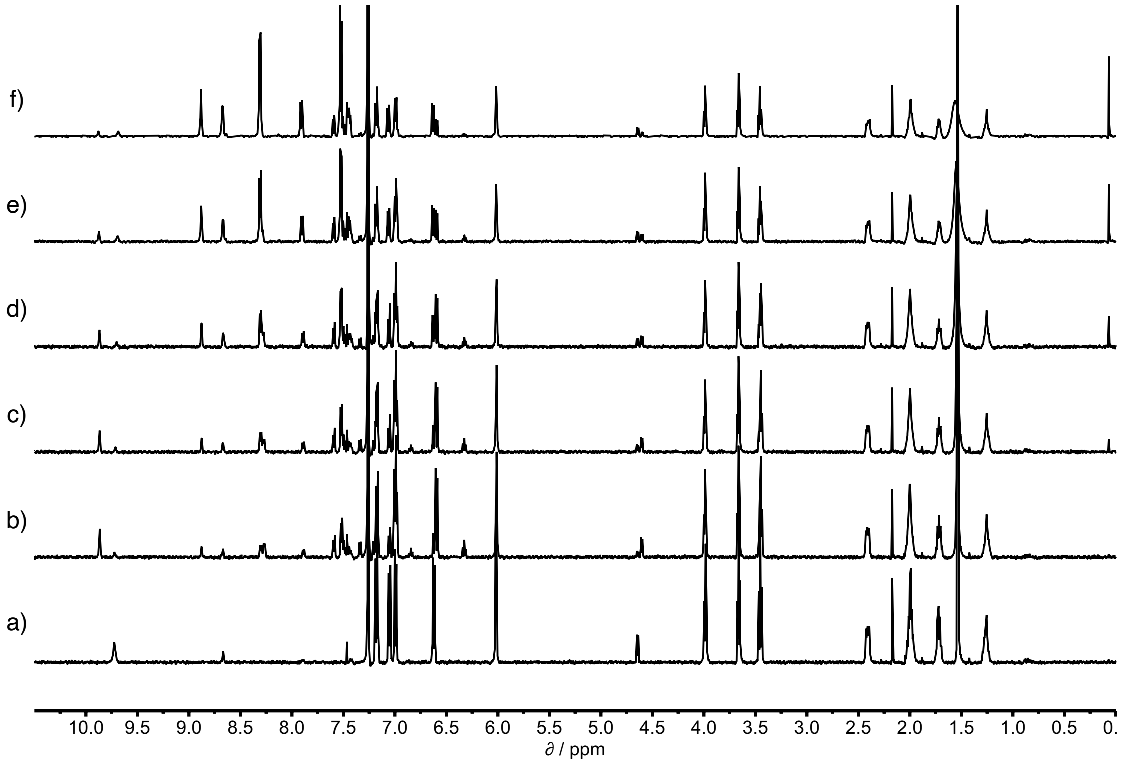

Figure S67. *500MHz ¹H NMR for titration of **6** into a mixture of **2**, **PPNO** and **6** in $CDCl_3$ at 298K. Concentrations are: a) **2**: 0.37 mM; **PPNO**: 0 mM; **6**: 0.43 mM; b) **2**: 0.30 mM; **PPNO**: 0.49 mM; **6**: 0.35 mM; c) **2**: 0.28 mM; **PPNO**: 0.46 mM; **6**: 0.49 mM; d) **2**: 0.25 mM; **PPNO**: 0.41 mM; **6**: 0.72 mM; e) **2**: 0.20 mM; **PPNO**: 0.33 mM; **6**: 1.06 mM; f) **2**: 0.16 mM; **PPNO**: 0.26 mM; **6**: 1.39 mM. Integration of selected proton signals indicated that K(**2•PPNO**) = 2.9±0.6 x K(**2•6**).*

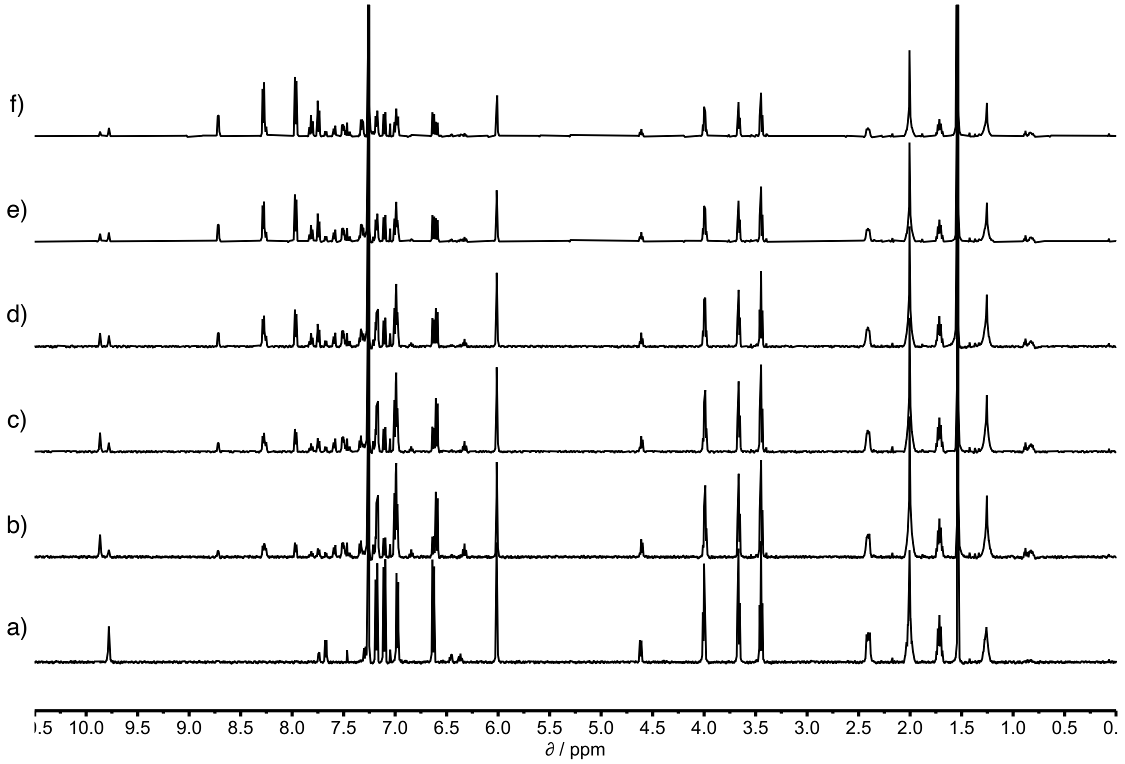

Figure S68. *500MHz ¹H NMR for titration of **7** into a mixture of **2**, **PPNO** and **7** in $CDCl_3$ at 298K. Concentrations are: a) **2**: 0.41 mM; **PPNO**: 0 mM; **7**: 0.44 mM; b) **2**: 0.34 mM; **PPNO**: 0.43 mM; **7**: 0.36 mM; c) **2**: 0.30 mM; **PPNO**: 0.38 mM; **7**: 0.48 mM; d) **2**: 0.25 mM; **PPNO**: 0.32 mM; **7**: 0.66 mM; e) **2**: 0.20 mM; **PPNO**: 0.25 mM; **7**: 0.84 mM; f) **2**: 0.15 mM; **PPNO**: 0.20 mM; **7**: 0.99 mM. Integration of selected proton signals indicated that K(**2•PPNO**) = 3.7±0.5 x K(**2•7**).*

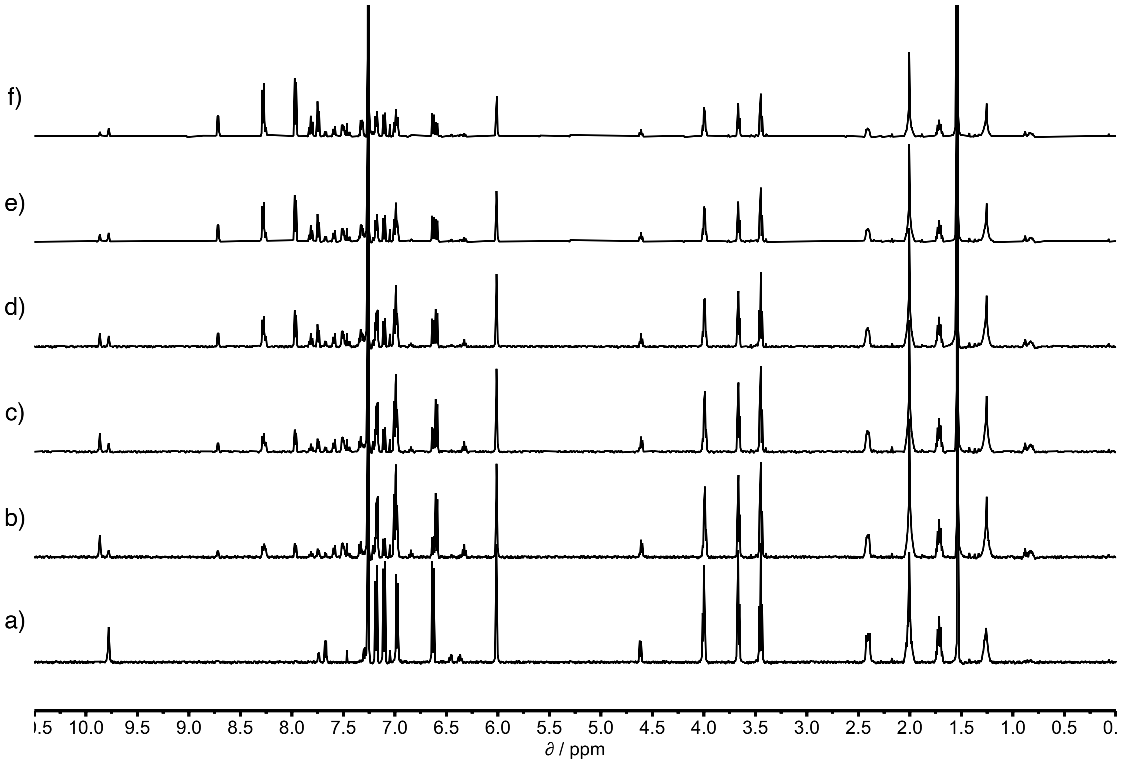

Figure S69. *500MHz ^{1}H NMR for titration of **8** into a mixture of **2**, **PPNO** and **8** in $CDCl_3$ at 298K. Concentrations are: a) **2**: 0.41 mM; **PPNO**: 0 mM; **8**: 0.44 mM; b) **2**: 0.324mM; **PPNO**: 0.43 mM; **8**: 0.36 mM; c) **2**: 0.30 mM; **PPNO**: 0.38 mM; **8**: 0.48 mM; d) **2**: 0.25 mM; **PPNO**: 0.32 mM; **8**: 0.66 mM; e) **2**: 0.20 mM; **PPNO**: 0.25 mM; **8**: 0.84 mM; f) **2**: 0.15 mM; **PPNO**: 0.20 mM; **8**: 0.99 mM. Integration of selected proton signals indicated that K(**2•PPNO**) = 8.1±1.3 x K(**2•8**).*

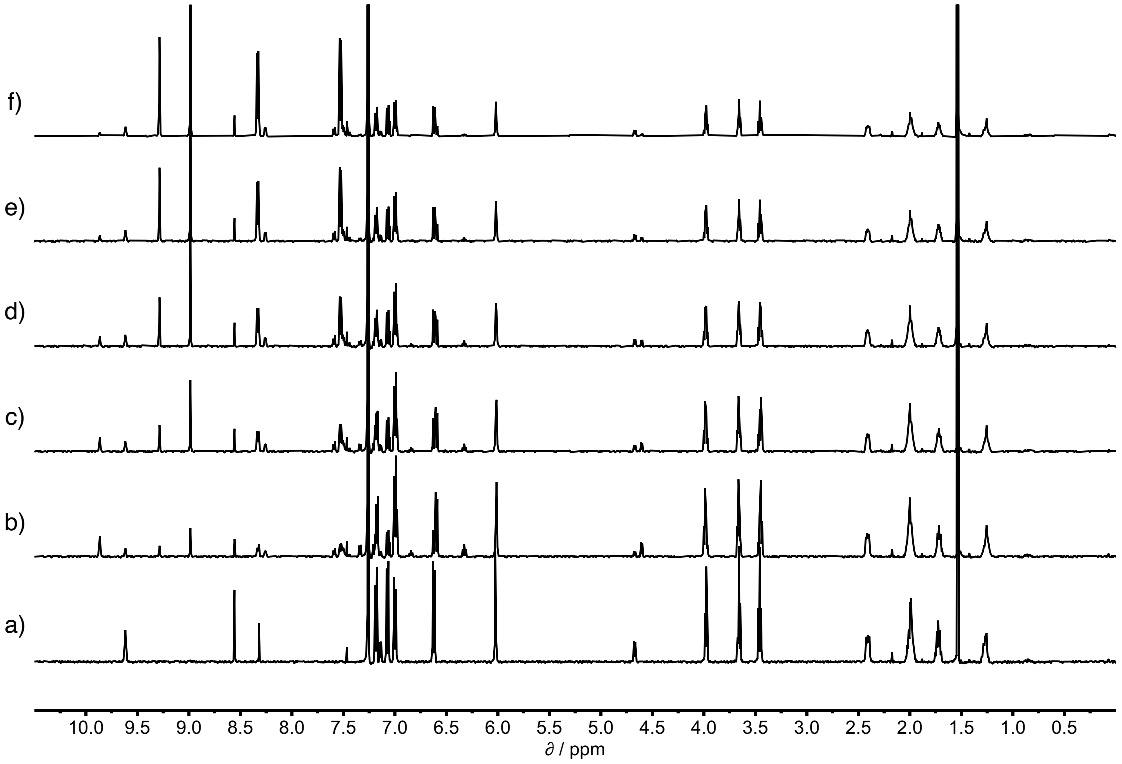

Figure S70. *500MHz ^{1}H NMR for titration of **9** into a mixture of **2**, **PPNO** and **9** in $CDCl_3$ at 298K. Concentrations are: a) **2**: 0.37 mM; **PPNO**: 0 mM; **9**: 0.42 mM; b) **2**: 0.32 mM; **PPNO**: 0.35 mM; **9**: 0.36 mM; c) **2**: 0.28 mM; **PPNO**: 0.30 mM; **9**: 0.63 mM; d) **2**: 0.23 mM; **PPNO**: 0.26 mM; **9**: 0.92 mM; e) **2**: 0.19 mM; **PPNO**: 0.21 mM; **9**: 1.20 mM; f) **2**: 0.15 mM; **PPNO**: 0.17 mM; **9**: 1.47 mM. Integration of selected proton signals indicated that K(**2•PPNO**) = 4.0±0.7 x K(**2•9**).*

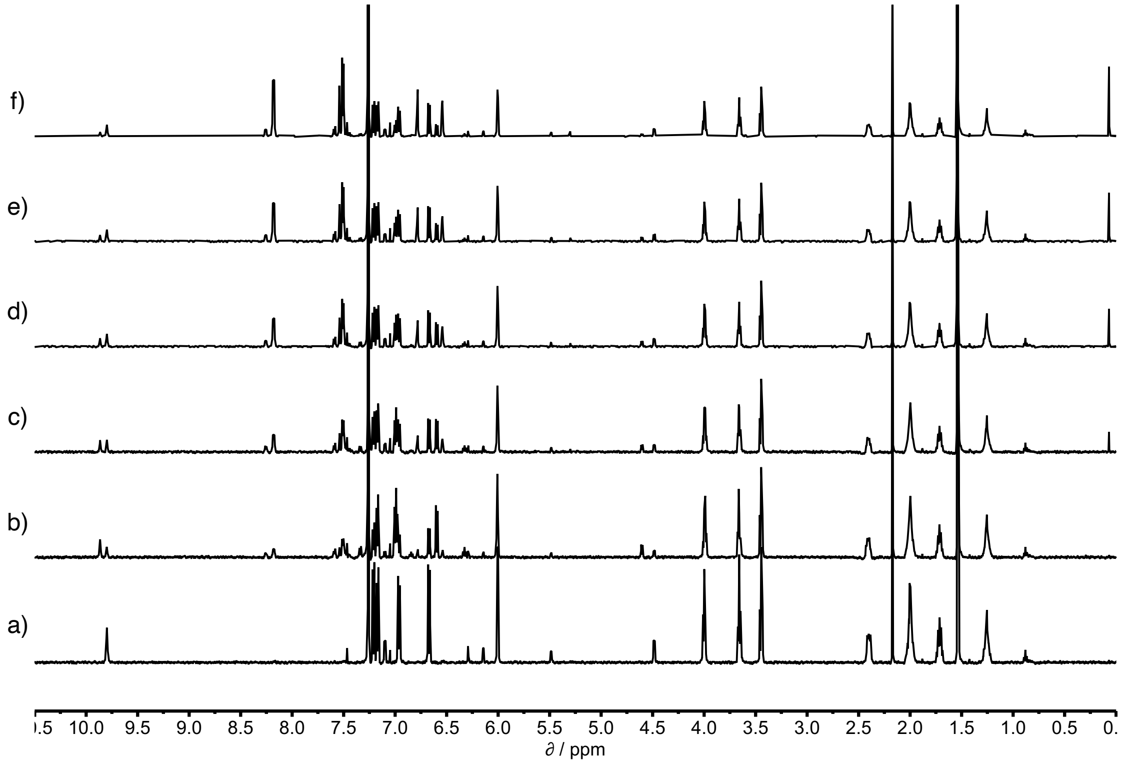

Figure S71. *500MHz ^1H NMR for titration of **10** into a mixture of **2**, **PPNO** and **10** in $CDCl_3$ at 298K. Concentrations are: a) **2**: 0.37 mM; **PPNO**: 0 mM; **10**: 0.43 mM; b) **2**: 0.32 mM; **PPNO**: 0.35 mM; **10**: 0.37 mM; c) **2**: 0.28 mM; **PPNO**: 0.30 mM; **10**: 0.64 mM; d) **2**: 0.25 mM; **PPNO**: 0.27 mM; **10**: 0.85 mM; e) **2**: 0.22 mM; **PPNO**: 0.24 mM; **10**: 1.03 mM; f) **2**: 0.19 mM; **PPNO**: 0.20 mM; **10**: 1.28 mM. Integration of selected proton signals indicated that K(**2•PPNO**) = 2.8±0.5 x K(**2•10**).*

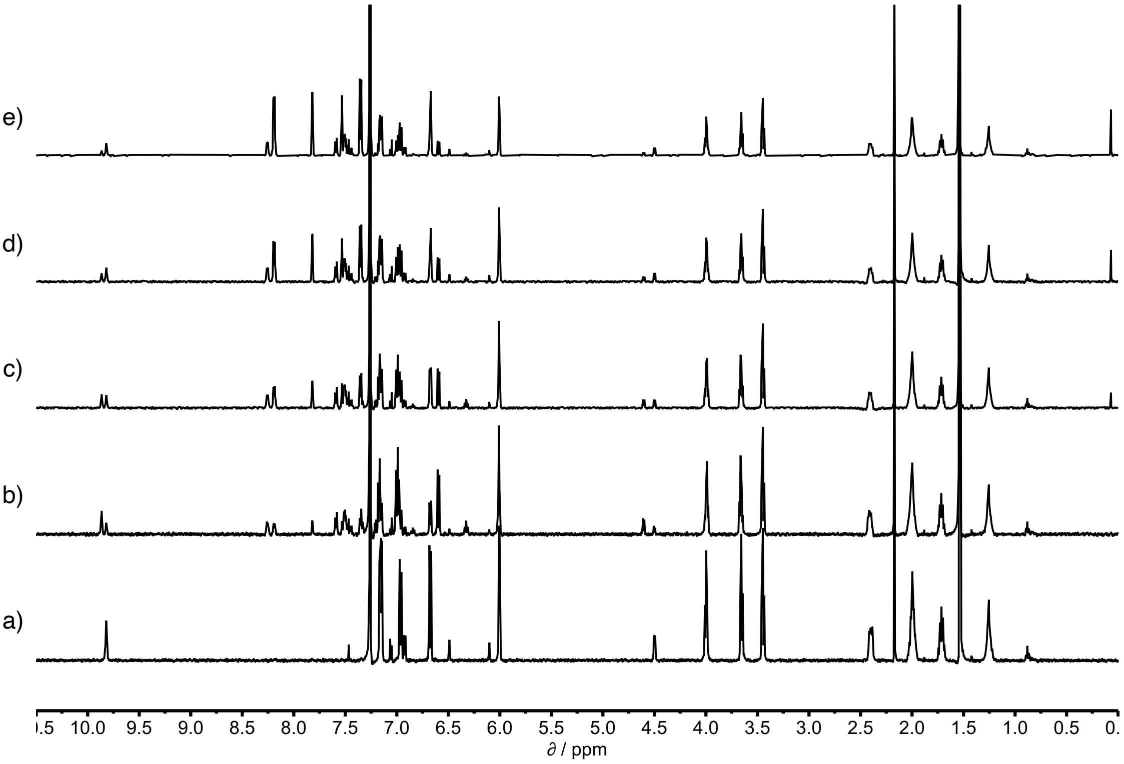

Figure S72. *500MHz ^1H NMR for titration of **11** into a mixture of **2**, **PPNO** and **11** in $CDCl_3$ at 298K. Concentrations are: a) **2**: 0.37 mM; **PPNO**: 0 mM; **11**: 0.43 mM; b) **2**: 0.30 mM; **PPNO**: 0.49 mM; **11**: 0.34 mM; c) **2**: 0.26 mM; **PPNO**: 0.43 mM; **11**: 0.61 mM; d) **2**: 0.22 mM; **PPNO**: 0.36 mM; **11**: 0.90 mM; e) **2**: 0.19 mM; **PPNO**: 0.30 mM; **11**: 1.18 mM. Integration of selected proton signals indicated that K(**2•PPNO**) = 1.8±0.2 x K(**2•11**).*

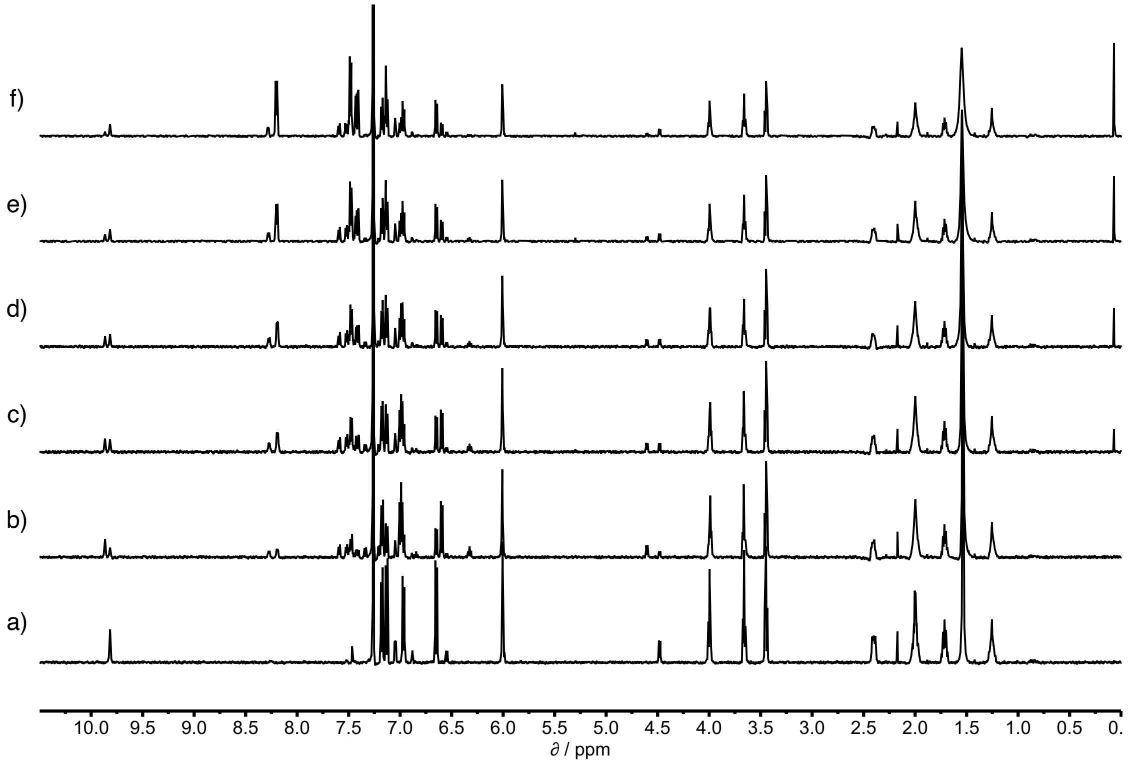

Figure S73. *500MHz* [1]*H NMR for titration of* ***12*** *into a mixture of* ***2****,* ***PPNO*** *and* ***12*** *in* $CDCl_3$ *at 298K. Concentrations are: a)* ***2****: 0.36 mM;* ***PPNO****: 0 mM;* ***12****: 0.45 mM; b)* ***2****: 0.30 mM;* ***PPNO****: 0.41 mM;* ***12****: 0.37 mM; c)* ***2****: 0.27 mM;* ***PPNO****: 0.36 mM;* ***12****: 0.56 mM; d)* ***2****: 0.24 mM;* ***PPNO****: 0.33 mM;* ***12****: 0.72 mM; e)* ***2****: 0.21 mM;* ***PPNO****: 0.28 mM;* ***12****: 0.95 mM; f)* ***2****: 0.17 mM;* ***PPNO****: 0.23 mM;* ***12****: 1.19 mM. Integration of selected proton signals indicated that K(****2•PPNO****) = 2.3±0.2 x K(****2•12****).*

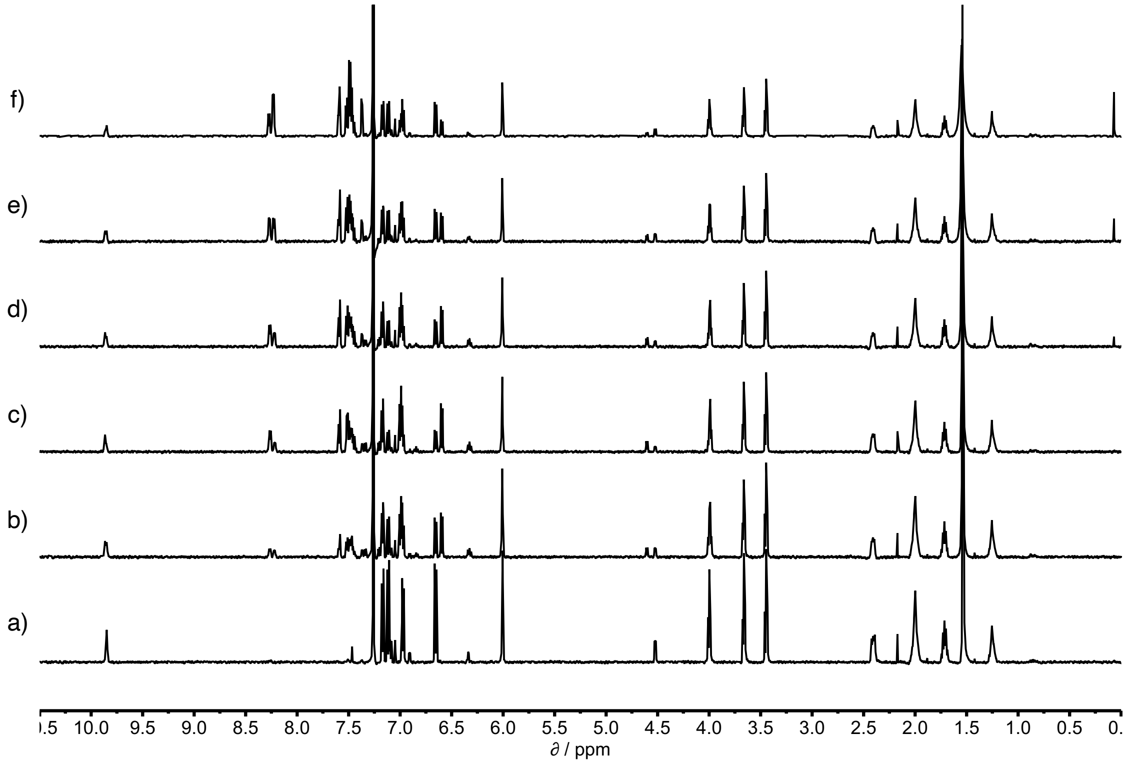

Figure S74. *500MHz* [1]*H NMR for titration of* ***13*** *into a mixture of* ***2****,* ***PPNO*** *and* ***13*** *in* $CDCl_3$ *at 298K. Concentrations are: a)* ***2****: 0.38 mM;* ***PPNO****: 0 mM;* ***13****: 0.51 mM; b)* ***2****: 0.31 mM;* ***PPNO****: 0.43 mM;* ***13****: 0.42 mM; c)* ***2****: 0.27 mM;* ***PPNO****: 0.72 mM;* ***13****: 0.36 mM; d)* ***2****: 0.25 mM;* ***PPNO****: 0.69 mM;* ***13****: 0.51 mM; e)* ***2****: 0.23 mM;* ***PPNO****: 0.63 mM;* ***13****: 0.78 mM; f)* ***2****: 0.20 mM;* ***PPNO****: 0.54 mM;* ***13****: 1.19 mM. Integration of selected proton signals indicated that K(****2•PPNO****) = 1.1±0.1 x K(****2•13****).*

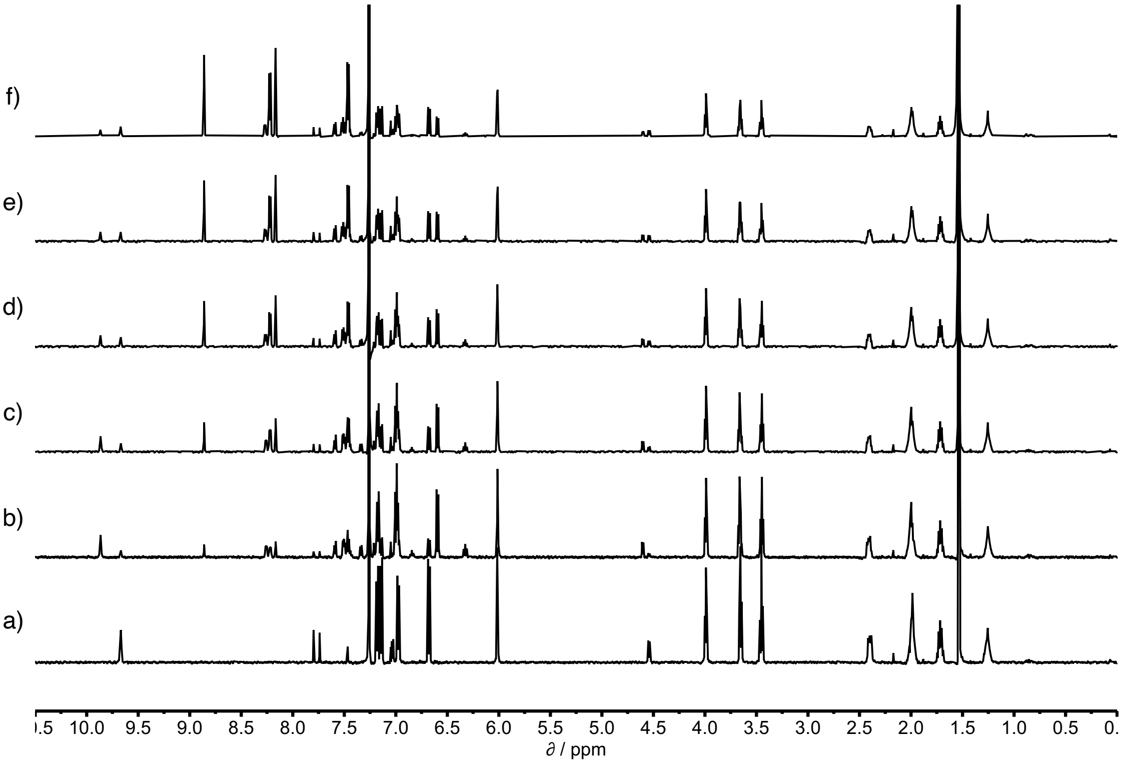

Figure S75. *500MHz ¹H NMR for titration of* ***14*** *into a mixture of* ***2***, ***PPNO*** *and* ***14*** *in* $CDCl_3$ *at 298K. Concentrations are: a)* ***2***: *0.37 mM;* ***PPNO***: *0 mM;* ***14***: *0.39 mM; b)* ***2***: *0.30 mM;* ***PPNO***: *0.49 mM;* ***14***: *0.32 mM; c)* ***2***: *0.26 mM;* ***PPNO***: *0.43 mM;* ***14***: *0.56 mM; d)* ***2***: *0.23 mM;* ***PPNO***: *0.38 mM;* ***14***: *0.75 mM; e)* ***2***: *0.21 mM;* ***PPNO***: *0.35 mM;* ***14***: *0.90 mM; f)* ***2***: *0.18 mM;* ***PPNO***: *0.29 mM;* ***14***: *1.14 mM. Integration of selected proton signals indicated that K(**2•PPNO**) = 3.2±0.4 x K(**2•14**).*

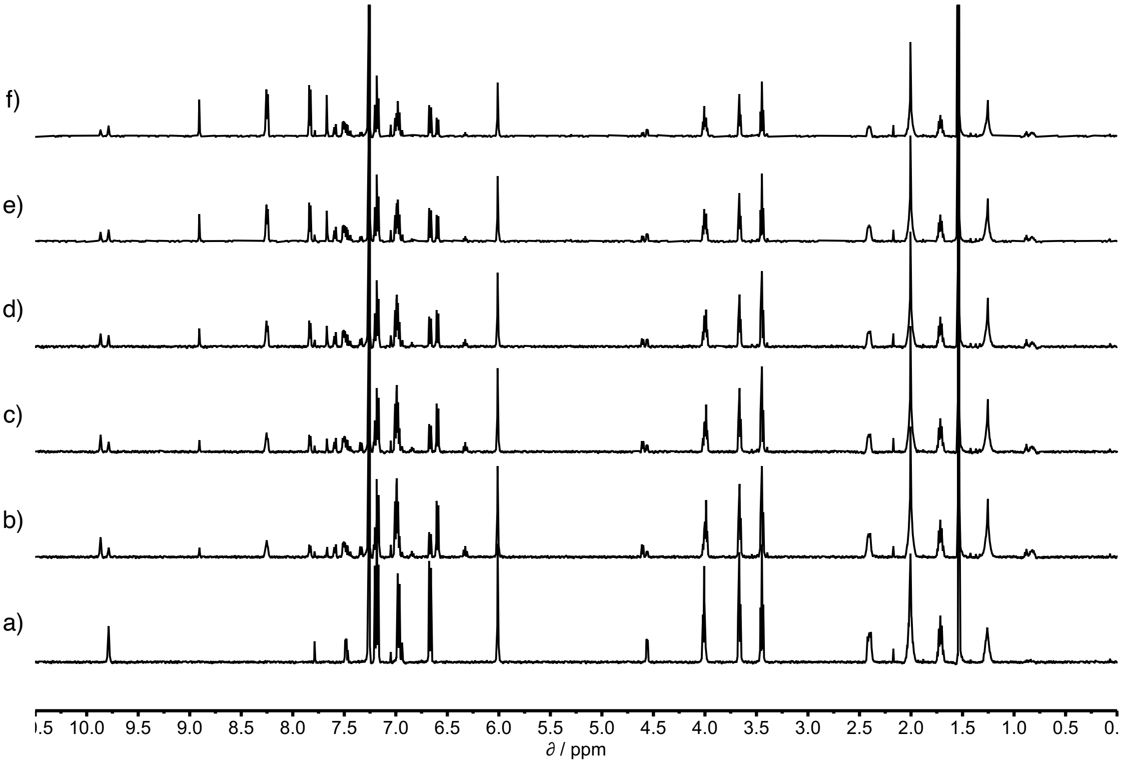

Figure S76. *500MHz ¹H NMR for titration of* ***15*** *into a mixture of* ***2***, ***PPNO*** *and* ***15*** *in* $CDCl_3$ *at 298K. Concentrations are: a)* ***2***: *0.44 mM;* ***PPNO***: *0 mM;* ***15***: *0.47 mM; b)* ***2***: *0.36 mM;* ***PPNO***: *0.46 mM;* ***15***: *0.38 mM; c)* ***2***: *0.34 mM;* ***PPNO***: *0.43 mM;* ***15***: *0.48 mM; d)* ***2***: *0.30 mM;* ***PPNO***: *0.38 mM;* ***15***: *0.64 mM; e)* ***2***: *0.26 mM;* ***PPNO***: *0.33 mM;* ***15***: *0.83 mM; f)* ***2***: *0.22 mM;* ***PPNO***: *0.28 mM;* ***15***: *1.01 mM. Integration of selected proton signals indicated that K(**2•PPNO**) = 2.7±0.4 x K(**2•15**).*

5. Analysis of binding

Table S1. *Thermodynamic parameters for formation of 1:1 complexes in water at 298 K determined by ^{1}H NMR competition (host **1**) and ITC (host **3**) experiments.*[a]

	Host						
	1		3				
**Guest**	K / M^{-1}[b]	$\Delta G°$ / kJ mol^{-1}[b]	K / M^{-1}[c]	$\Delta G°$ / kJ mol^{-1}[c]	$\Delta H°$ / kJ mol^{-1}[c]	$-T\Delta S°$ / kJ mol^{-1}[c]	N[c]
**PNO**	$3.5 \pm 0.1 \times 10^{6}$	-37.4 ± 0.0	$3.3 \pm 0.6 \times 10^{5}$	-31.5 ± 0.4	-34.4 ± 0.3	2.8 ± 0.6	0.7 ± 0.0
**5**	$1.0 \pm 0.3 \times 10^{10}$	-57.1 ± 0.8	$7.4 \pm 0.0 \times 10^{5}$	-33.5 ± 0.0	-38.8 ± 1.3	5.2 ± 1.2	0.8 ± 0.1
**6**	$2.0 \pm 0.6 \times 10^{9}$	-53.0 ± 0.7	$1.0 \pm 0.0 \times 10^{6}$	-34.4 ± 0.1	-49.0 ± 0.7	14.6 ± 0.8	0.7 ± 0.0
**7**	$3.2 \pm 0.9 \times 10^{9}$	-54.2 ± 0.7	$1.2 \pm 0.1 \times 10^{6}$	-34.6 ± 0.1	-50.3 ± 0.6	15.7 ± 0.4	0.6 ± 0.1
**8**	$3.8 \pm 1.1 \times 10^{9}$	-54.7 ± 0.7	$9.8 \pm 0.1 \times 10^{5}$	-34.2 ± 0.0	-38.9 ± 3.5	4.6 ± 3.6	0.8 ± 0.1
**9**	$1.0 \pm 0.3 \times 10^{9}$	-51.4 ± 0.6	$6.9 \pm 1.3 \times 10^{5}$	-33.3 ± 0.5	-46.5 ± 0.1	13.1 ± 0.6	0.7 ± 0.0
**10**	$3.3 \pm 0.9 \times 10^{8}$	-48. 6 ± 0.6	$9.3 \pm 3.9 \times 10^{5}$	-34.1 ± 1.0	-43.5 ± 8.9	9.3 ± 7.8	0.7 ± 0.1
**11**	$4.3 \pm 1.1 \times 10^{8}$	-49.3 ± 0.6	$8.5 \pm 0.1 \times 10^{5}$	-33.8 ± 0.0	-42.8 ± 1.0	8.9 ± 1.1	0.7 ± 0.0
**12**	$1.3 \pm 0.3 \times 10^{9}$	-51.9 ± 0.6	$1.0 \pm 0.3 \times 10^{6}$	-34.2 ± 0.8	-42.8 ± 1.8	8.5 ± 2.6	0.7 ± 0.0
**13**	$1.4 \pm 0.3 \times 10^{9}$	-52.1 ± 0.6	$9.1 \pm 0.1 \times 10^{5}$	-34.0 ± 0.2	-41.8 ± 2.1	7.7 ± 2.4	0.7 ± 0.0
**14**	$1.1 \pm 0.3 \times 10^{9}$	-51.7± 0.6	$7.2 \pm 3.5 \times 10^{5}$	-33.4 ± 1.2	-42.5 ± 0.0	9.1 ± 1.2	0.7 ± 0.0
**15**	$1.2 \pm 0.3 \times 10^{9}$	-51.7± 0.6	$9.7 \pm 1.6 \times 10^{5}$	-34.2 ± 0.4	-43.6 ± 1.6	9.4 ± 1.1	0.7 ± 0.0

[a] Every measurement was repeated at least twice, and errors are quoted as twice the standard deviation. [b] Determined by pair-wise ^{1}H NMR competitive experiments. [c] The N parameter is a variable fitted in the ITC experiment, which is usually taken as an indication of stoichiometry. The values are close to one, which suggests that there is a 1:1 complex. The fact that the values are consistently low (0.7) could be due to a discrepancy in the concentration of host or due to host aggregation.

Table S2. *Thermodynamic parameters for formation of 1:1 complexes in chloroform at 298 K determined by* ^{1}*H NMR competition (host* ***2****) and ITC (host* ***4****) experiments.*[a]

	**Host**						
	**2**		**4**				
**Guest**	K / M^{-1b}	ΔG° / kJ mol^{-1b}	K / M^{-1c}	ΔG° / kJ mol^{-1c}	ΔH° / kJ mol^{-1c}	-TΔS° / kJ mol^{-1c}	N^{c}
**PNO**	7.5 ± 0.7 x 10^{6}	-39.2 ± 0.2	1.3 ± 0.0 x 10^{6}	-34.9 ± 0.1	-61.0 ± 2.7	26.0 ± 2.5	1.1 ± 0.5
**5**	2.3 ± 0.4 x 10^{8}	-47.7 ± 0.4	4.8 ± 1.5 x 10^{5}	-32.2 ± 0.3	-54.4 ± 17.3	22.0 ± 18.1	1.1 ± 0.2
**6**	4.5 ± 1.2 x 10^{7}	-43.7 ± 0.7	6.9 ± 3.6 x 10^{5}	-33.3 ± 1.3	-60.9 ± 4.9	27.5 ± 3.7	0.9 ± 0.2
**7**	3.2 ± 0.5 x 10^{7}	-42.8 ± 0.4	1.7 ± 0.1 x 10^{6}	-35.8 ± 0.0	-71.6 ± 1.1	36.1 ± 1.1	0.7 ± 0-0
**8**	1.6 ± 0.4 x 10^{7}	-41.1 ± 0.6	1.2 ± 0.3 x 10^{6}	-35.0 ± 0.3	-62.2 ± 6.1	27.5 ± 6.6	0.8 ± 0.0
**9**	3.2 ± 0.8 x 10^{7}	-42.8 ± 0.6	4.0 ± 1.3 x 10^{5}	-31.9 ± 0.9	-60.0 ± 0.8	28.0 ± 1.7	0.8 ± 0.1
**10**	4.6 ± 1.1 x 10^{7}	-43.7 ± 0.6	8.2 ± 1.6 x 10^{5}	-33.6 ± 0.7	-61.7 ± 0.1	27.9 ± 0.6	0.9 ± 0.1
**11**	7.4 ± 1.5 x 10^{7}	-44.9 ± 0.5	1.1 ± 0.3 x 10^{6}	-33.6 ± 1.0	-56.0 ± 5.8	21.5 ± 6.2	0.9 ± 0.1
**12**	5.6 ± 1.0 x 10^{7}	-44.2 ± 0.4	1.0 ± 0.7 x 10^{6}	-34.4 ± 2.1	-65.8 ± 1.6	31.6 ± 0.1	0.8 ± 0.0
**13**	1.2 ± 0.2 x 10^{8}	-46.1 ± 0.5	7.5 ± 3.5 x 10^{5}	-33.2 ± 1.5	-58.4 ± 0.8	24.9 ± 0.4	0.9 ± 0.1
**14**	4.1 ± 0.8 x 10^{7}	-43.4 ± 0.5	5.6 ± 0.8 x 10^{5}	-33.9 ± 0.7	-62.0 ± 3.1	29.2 ± 3.3	0.8 ± 0-1
**15**	5.3 ± 1.2 x 10^{7}	-43.8 ± 0.6	1.3 ± 0.2 x 10^{6}	-35.0 ± 2.5	-59.5 ± 20.6	24.6 ± 20.2	0.8 ± 0.1

[a] Every measurement was repeated at least twice, and errors are quoted as twice the standard deviation. [b] Determined by pair-wise ^{1}*H NMR competitive experiments. [c] The N parameter is a variable fitted in the ITC experiment, which is usually taken as an indication of stoichiometry. The values are close to one, which suggests that there is a 1:1 complex.*

6. Analysis of structures

Table S3. *Limiting complexation-induced changes in ^{1}H NMR chemical shift (Δδ in ppm) for the guest signals in complex A of the DMC.*[a]

		Solvent													
		D_2O							$CDCl_3$						
**Guest**	**R**	H_1	H_2	H_{3a}	H_{3b}	H_{4a}	H_{4b}	H_5	H_1	H_2	H_{3a}	H_{3b}	H_{4a}	H_{4b}	H_5
**Phenyl**	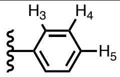	-4.0	-0.7	-0.8	-	-1.6	-	-1.2	-3.7	-0.3	-0.3	-	-1.2	-	-0.6
**5**	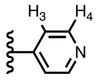	-4.0	-0.7	-1.4	-	-1.8	-	-	-3.7	-0.4	-0.5	-	n.d.	-	-
**6**	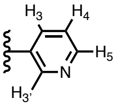	-4.0	-0.8	-1.0	n.d.	-1.8	-	-1.2	-3.7	-0.4	n.d.	-0.2	n.d.	n.d.	n.d.
**7**	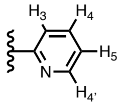	-4.1	-0.7	-1.9	-	-1.9	-1.1	-1.3	-3.7	-0.4	-0.5	-	-1.5	-1.0	-0.9
**8**	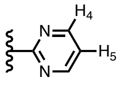	-4.0	-0.7	-	-	-1.3	-	-1.5	-3.7	-0.3	-	-	-1.2	-	-1.1
**9**	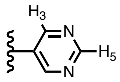	-4.0	-0.8	-0.6	-	-	-	-1.3	-3.7	-0.4	-0.4	-	-	-	-1.0
**10**	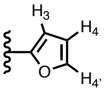	-4.0	-0.7	-1.5	-	-1.7	-1.9	-	-3.7	-0.4	-0.6	-	-1.1	-1.3	-
**11**	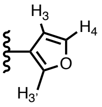	-4.0	-0.8	-1.1	n.d.	-1.6	-	-	-3.7	-0.4	-0.6	-0.8	-1.0	-	-
**12**	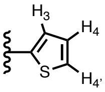	-4.0	-0.8	-1.4	-	-1.7	-1.3	-	-3.7	-0.5	-0.5	-	-1.2	-0.9	-
**13**	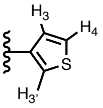	-4.0	-0.8	-1.0	-2.0	-1.0	-	-	-3.7	-0.4	-0.5	-0.5	-1.1	-	-
**14**	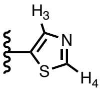	-4.0	-0.8	-1.0	-	-1.6	-	-	-3.7	-0.4	-0.4	-	-1.1	-	-
**15**	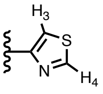	-4.0	-0.7	-1.8	-	-1.1	-	-	-3.7	-0.4	-0.8		-1.1	-	-

[a] *n.d. values could not be determined.*

Table S4. *Limiting complexation-induced changes in ^{1}H NMR chemical shift (Δδ in ppm) in deuterochloroform for the host signals in complex A of the DMC.*

		2						
**Guest**	**R**	**H_a**	**H_b**	**H_c**	**H_d**	**H_e**	**H_f**	**H_g**
**Phenyl**	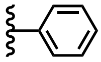	+2.5	+0.1	-0.1	-0.2	-0.4	-0.2	0.0
**5**	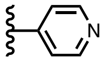	+2.3	+0.1	-0.1	-0.2	-0.4	-0.2	0.0
**6**	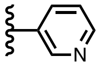	+2.3	+0.1	-0.1	-0.2	-0.4	-0.2	0.0
**7**	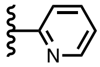	+2.4	+0.1	-0.1	-0.2	-0.3	-0.2	0.0
**8**	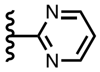	+2.3	+0.1	-0.1	-0.2	-0.2	-0.1	0.0
**9**	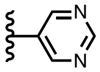	+2.2	+0.1	-0.1	-0.2	-0.4	-0.2	0.0
**10**	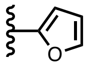	+2.4	+0.1	-0.1	-0.2	-0.2	-0.1	0.0
**11**	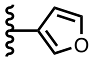	+2.4	+0.1	-0.1	-0.2	-0.3	-0.1	0.0
**12**	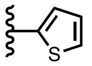	+2.4	+0.1	-0.1	-0.2	-0.3	-0.2	0.0
**13**	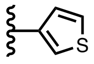	+2.5	+0.1	-0.1	-0.2	-0.3	-0.2	0.0
**14**	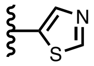	+2.3	+0.1	-0.1	-0.2	-0.3	-0.1	0.0
**15**	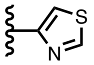	+2.4	+0.1	-0.1	-0.2	-0.3	-0.1	0.0

Table S5. *Differences between the ^{1}H chemical shift of the host signal in host•guest complexes compared with the chemical shift in the corresponding host•**Phenyl** complex (Δδ in ppm) at 298 K.*

		Solvent													
		D_2O							$CDCl_3$						
**Guest**	**R**	**H_a**	**H_b**	**H_c**	**H_d**	**H_e**	**H_f**	**H_g**	**H_a**	**H_b**	**H_c**	**H_d**	**H_e**	**H_f**	**H_g**
**5**	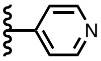	n.d.	0.0	0.0	0.0	+0.1	+0.1	0.0	-0.2	0.0	0.0	0.0	0.0	+0.1	0.0
**6**	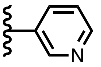	n.d.	0.0	0.0	0.0	0.0	0.0	0.0	-0.1	0.0	0.0	0.0	+0.1	0.0	0.0
**7**	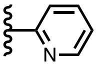	n.d.	0.0	0.0	0.0	0.0	0.0	0.0	-0.1	0.0	0.0	0.0	+0.1	0.0	0.0
**8**	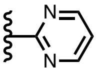	n.d.	0.0	0.0	0.0	+0.1	0.0	0.0	-0.2	0.0	0.0	0.0	+0.2	+0.1	0.0
**9**	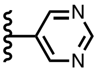	n.d.	0.0	0.0	+0.1	0.0	-0.1	0.0	-0.2	0.0	0.0	0.0	+0.1	0.0	0.0
**10**	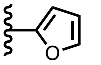	n.d.	0.0	0.0	0.0	+0.2	+0.1	0.0	-0.1	0.0	0.0	0.0	+0.2	+0.1	0.0
**11**	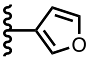	n.d.	0.0	0.0	0.0	+0.1	+0.1	0.0	0.0	0.0	0.0	0.0	+0.2	+0.1	0.0
**12**	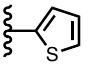	n.d.	0.0	0.0	0.0	+0.1	+0.1	0.0	0.0	0.0	0.0	0.0	+0.1	0.0	0.0
**13**	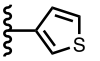	n.d.	0.0	0.0	0.0	+0.1	+0.1	0.0	0.0	0.0	0.0	0.0	+0.1	+0.1	0.0
**14**	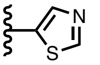	n.d.	0.0	0.0	0.0	+0.1	+0.1	0.0	-0.2	0.0	0.0	0.0	+0.1	+0.1	0.0
**15**	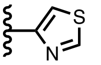	n.d.	0.0	0.0	0.0	+0.1	+0.1	0.0	-0.1	0.0	0.0	0.0	+0.2	+0.1	0.0

[a] *n.d. values could not be determined.*

7. References

1 L. Escobar and P. Ballester, *Org. Chem. Front.*, 2019, **6**, 1738–1748.

2 A. Díaz-Moscoso, D. Hernández-Alonso, L. Escobar, F. A. Arroyave and P. Ballester, *Org. Lett.*, 2017, **19**, 226–229.

3 L. Escobar, A. Díaz-Moscoso and P. Ballester, *Chem. Sci.*, 2018, **9**, 7186–7192.
